# Uterine Extracellular Vesicles Can Emulate the Long‐Term Effects of Post‐Partum Negative Energy Balance in Dairy Cows

**DOI:** 10.1002/mrd.70062

**Published:** 2025-10-23

**Authors:** Juliana Germano Ferst, Matheus Andrade Chaves, Amanda Nespolo Silva, Schaienni Fontoura Saldanha, Rogério Ferreira, Ricardo Perecin Nociti, Angélica Camargo dos Santos, Samuel Volpe Souza, Marcos Roberto Chiaratti, Guilherme Pugliesi, Felipe Perecin, Flávio Vieira Meirelles, Juliano Coelho da Silveira

**Affiliations:** ^1^ Department of Veterinary Medicine, School of Animal Science and Food Engineering University of São Paulo Pirassununga Brazil; ^2^ Department of Animal Production Santa Catarina State University Chapecó Brazil; ^3^ Department of Genetics and Evolution Federal University of São Carlos São Carlos Brazil; ^4^ Department of Animal Reproduction, School of Veterinary Medicine and Animal Science University of São Paulo Pirassununga Brazil

**Keywords:** cattle, metabolic status, transition period, uterine cells

## Abstract

Dairy cows often experience a period of negative energy balance (NEB) during the post‐calving period, which can significantly impact economic outcomes due to extended calving‐to‐conception intervals and overall reduced fertility. This reduction is due, in part, to the impact on uterine biology by high nonesterified fatty acids (NEFA) and beta‐hydroxybutyrate concentration. The uterine fluid (UF) contains small extracellular vesicles (UF‐EVs) that, through their cargo, including microRNAs (miRNAs), respond to metabolic stress, affecting the uterine environment. This study aimed to assess the long‐term impact of NEB intensity on the uterine environment of dairy cows. Post‐partum dairy cows were classified based on NEFA concentrations in their blood during the 3 weeks post‐calving as having either Low or High NEB. At 30 and 60 DPC, the synchronization protocol was started, and UF samples were collected (corresponding to ~15 days after initiation of the synchronization protocol) to isolate UF‐EVs and uterine epithelial cells for miRNA and transcriptome profiling. We also investigated whether UF‐EVs could modulate epithelial uterine naïve cells. Our results indicate that the uterine environment of dairy cows experiencing a High NEB post‐calving is unfavorable for embryo development at 60‐day post‐calving. Importantly, we show that UF‐EVs can reproduce this phenotype in epithelial uterine naïve cells, suggesting that UF‐EVs may act as modulators of the uterine response to metabolic challenges.

## 1 Introduction

Genetic selection to enhance milk production in dairy cows has negatively impacted reproductive cells, concurrently reducing fertility. While heifers have calving rates of approximately 55%–60%, these rates decline to around 35%–40% in lactating cows (Lonergan et al. 2016), likely due to the metabolic challenges encountered during the transition period. Negative energy balance (NEB) is a primary contributor to reduced fertility during the post‐calving period (Butler 2000; Serbetci et al. 2023). NEB occurs when a cow's energy demand for maintenance and lactation exceeds its dietary energy intake (Bauman and Bruce Currie 1980; VandeHaar et al. 2016). To compensate for the energy required for milk production, the cow metabolizes fat reserves through lipogenesis and ketogenesis, leading to elevated blood levels of nonesterified fatty acids (NEFA) and beta‐hydroxybutyrate (BHB) (Herdt 2000). These metabolites are known to adversely affect uterine cells (Chankeaw et al. 2018; P. Li et al. 2019; Qin et al. 2020). The resulting impact on uterine biology is directly associated with the development of uterine diseases, which prolong the calving‐to‐conception interval and increase the risk of failure in pregnancy establishment (Bicalho et al. 2017; Esposito et al. 2014; LeBlanc 2023; Nicola et al. 2022; Ospina et al. 2010a).

Due to the complex physiology of the uterus, the uterine environment plays a crucial role in enabling uterine remodeling during the transition period (Wathes et al. 2011). One mechanism for achieving uterine remodeling involves intercellular and intracellular communication, which can be mediated by extracellular vesicles (EVs) among other factors (Valadi et al. 2007). EVs are lipid bilayer‐enclosed structures secreted by different cell types and have been identified in several body fluids, including follicular fluid (da Silveira et al. 2012), blood (Cleys et al. 2014), oviduct fluid (Lopera‐Vásquez et al. 2016), and uterine fluid (UF) (Burns et al. 2016; Ng et al. 2013). EVs are classified according to their size, biosynthesis, and contents (György et al. 2011). EVs carry a range of biomolecules, such as proteins, lipids, RNAs, and microRNAs (Al‐Dossary and Martin‐DeLeon 2016; Da Silveira et al. 2015; Valadi et al. 2007). MicroRNAs (miRNAs) are small (~22 nucleotides) noncoding molecules that regulate gene expression post‐transcriptionally by repressing target mRNA in various cell types (Bartel 2018). Recent studies have shown that EVs from uterine fluid (UF‐EVs) modulate key reproductive events, such as pregnancy establishment, maternal‐embryo communication, and regulation of the maternal immune system to facilitate embryo attachment (Chen et al. 2022; Fazeli and Godakumara 2024). Based on these findings, EVs are believed to influence numerous reproductive processes by delivering molecular signals under normal physiological conditions and in response to stressors (Simon et al. 2018). Notably, metabolic stress, such as NEB, is associated with the downregulation of EV‐coupled miRNAs in the follicular fluid of dairy cows (Hailay et al. 2019). Thus, the metabolic stress related to the transition period may induce alterations in EVs and uterine cells.

The period of greatest mobilization of body reserves occurs around 2 weeks post‐calving, when NEB is most intense (Drackley 1999). However, the negative effects of NEB on reproductive cells persist beyond this period of intense mobilization (Girard et al. 2015; Horlock et al. 2020). Similarly, the consequences of conditions such as endometritis, which can arise as a result of NEB, may continue to be observed even after apparent uterine recovery (Silva et al. 2024). Recently, we demonstrated that uterine cells exhibit epigenetic marks following in vitro exposure to high concentrations of NEFA and BHB (Ferst et al. 2021), which could explain the prolonged effects of NEB on uterine cells. Given that EVs contain a variety of biomolecules and mediate intercellular communication in response to metabolic stress, we hypothesize that the uterine environment is differentially affected depending on the intensity of NEB post‐calving, and that these effects may be sustained over time due to the biomolecular content of EVs. Therefore, the aim of this study was to investigate the effects of NEB intensity on the miRNA profile of UF‐EVs and the transcriptome of uterine epithelial cells (UEpCs) collected at 30‐ and 60‐day post‐calving (DPC). To characterize the uterine environment, samples were collected from dairy cows during two critical periods: near the metabolic challenge (30 DPC) and the time when cows typically begin to be inseminated post‐calving (60 DPC), at a time when the uterus is preparing to receive the embryo (5 days after artificial insemination). All the animals were inseminated to mimic the physiological condition of a uterus preparing to receive an embryo following fertilization. Furthermore, to determine whether UF‐EVs can modulate uterine cells not exposed to metabolic challenges (epithelial uterine naïve cells), we cultured these cells with UF‐EVs and evaluated their miRNA profiles. Altogether, the experiments conducted in this study aim to understand the impact of NEB intensity on the uterine environment and assess whether the uterus is prepared to receive an embryo at 60 DPC in dairy cows.

## 2 Materials and Methods

The experimental procedure was approved by the University of São Paulo Research Ethics Committee (protocol number: 4628211021) and adhered to ethical principles for animal research. Unless otherwise specified, all chemicals and reagents were purchased from Sigma‐Aldrich/Merck Chemicals Company (St. Louis, United States of America).

### 2.1 Animals

**Figure 1:**
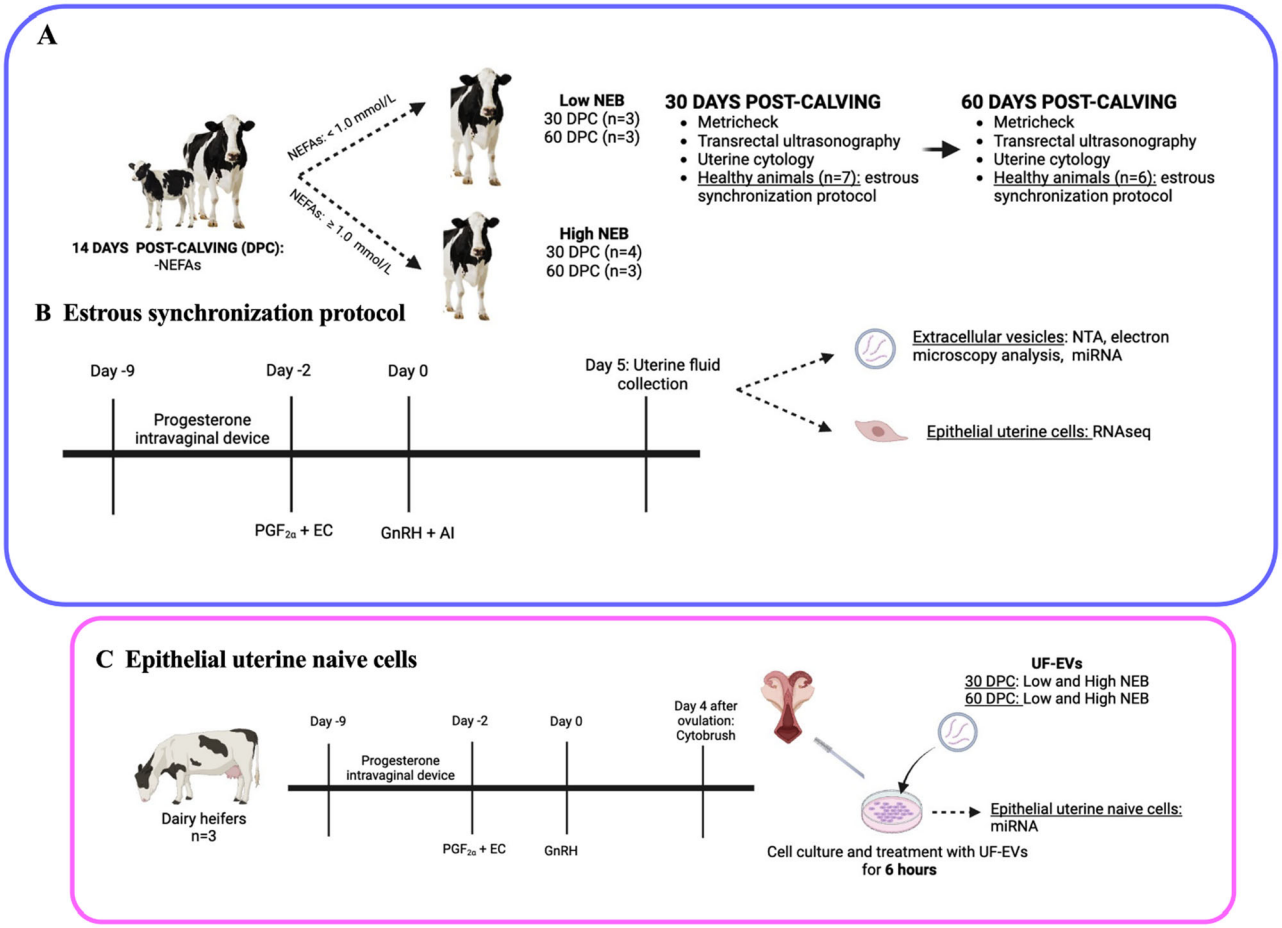
Schematic overview of the experimental model. (A) Blood samples were collected from individual cows (*n* = 7) at 7‐, 14‐, and 21‐day post‐calving (DPC) to determine the animal's energy status based on NEFA concentration. At 30 DPC, animals were evaluated by metricheck, transrectal ultrasonography, and uterine cytology. Only healthy animals (Low NEB = 3; High NEB = 4) had the estrous cycle synchronized. At 60 DPC the same animals were reassessed for reproductive parameters, and healthy animals (Low NEB = 3; High NEB = 3) had the estrous cycle synchronized. (B) After evaluation, the animals were submitted to an estrus synchronization protocol (PGF: prostaglandin F_2α_; EC: estradiol cypionate, GnRH: gonadotropin‐releasing hormone, AI: artificial insemination) and at 5 days after AI, the uterine fluid collection was performed (Day 0 = day of AI). Extracellular vesicles and epithelial uterine cells were separated from the uterine fluid. (C) To collect epithelial uterine naïve cells, the estrous cycles of dairy heifers were synchronized and 4 days after estrous, luminal epithelial cells from the ipsilateral horn to the corpus luteum were collected in vivo with a cytology brush (*n* = 3). After collection, cells were cultured, subcultures were performed, and cells were treated for 6 h with extracellular vesicles from uterine fluid (UF‐EVs). BHB, β‐hydroxybutyrate; NEB, negative energy balance; NEFA, nonesterified fatty acids. Created with BioRender (https://biorender.com/).

The study involved 16 multiparous Holstein Friesian (*n* = 16) housed at the dairy farm of the University of São Paulo. Sample collection took place from May to October. After parturition, cows were kept in a free‐stall setting. The farm maintained an average milk production of 27 kg/cow/day (from a total of 52 cows), with 177 days in milk and a calving interval of 70.3 days. At the onset of lactation, cows were milked twice daily at 7:00 h a.m. and 14:00 h p.m., approximately. Cows were fed a base diet consisting of alfalfa silage, corn silage, and supplemented with *Minerthal Núcleo Leite MD* (Minerthal, Agricultural Products Ltd., Brazil), with ad libitum access to fresh water. The body condition score, milk yield, temperature, and blood samples were monitored weekly until 63 DPC. At 30 DPC, animals were evaluated using metricheck, transrectal ultrasonography, and uterine cytology (Figure 1A). Uterine discharge samples were collected using a Metricheck device and scored on a scale from 0 to 3 based on the criteria established by Sheldon et al. (2006). Transrectal ultrasonography was performed to detect intrauterine contents, assess uterine wall thickness, and evaluate the presence of a corpus luteum (CL). In addition, uterine cytology was carried out to identify subclinical endometritis by determining the percentage of polymorphonuclear cells, according to the criteria described by Sheldon et al. (2006). To minimize confounding factors, cows with uterine conditions (e.g., clinical or subclinical endometritis) based on the criteria defined by Sheldon et al. (2006), calving difficulties or other clinical diseases were excluded from the experiment. Consequently, the study sample included seven healthy cows (Low NEB = 3; High NEB = 4), which is acknowledged as a limitation of this study. The estrous cycle was then synchronized using a progesterone‐releasing intravaginal device (IVD; CIDR, Zoetis, United States of America, 1.9 g progesterone), 2 mg estradiol benzoate (EB; Betaproginn, Boehringer Ingelheim, Germany), and prostaglandin F_2α_ analog (PGF_2α_; 500 μg cloprostenol, Cioprostinn, Boehringer Ingelheim, Germany), which were administered intramuscularly. The IVD was removed after 7 days, followed by the administration of estradiol cypionate (1 mg; EC; SincroCP, Ourofino Saúde Animal, Brazil) and PGF_2α_ (500 μg). On day 9, cows received a GnRH analog (0.01 mg; Sincroforte, Ourofino Saúde Animal, Brazil) and were inseminated at a fixed time without estrus detection. Frozen‐thawed semen from the same bull and batch (donated by ABS Brazil) was used. This procedure was performed in all animals to mimic the physiological condition of a uterus preparing to receive an embryo following fertilization. UF was collected 5 days after AI (Figure 1B). Following UF collection, PGF2α was administered to the cows. At 60 DPC (total *n* = 6; Low NEB = 3; High NEB = 3), the same procedures for reproductive evaluation, estrous cycle synchronization, AI and UF collection were repeated. One animal was excluded from the experiment between 30 and 60 DPC due to the development of disease.

### 2.2 Blood Serum Metabolite Analysis

Blood samples were collected from individual cows (*n* = 7) 3 weeks post‐calving. Samples were cooled for 1 h at 4°C and centrifuged at 400 g for 40 min to separate serum from blood cells and clotting factors. Serum samples from individual post‐calving cows were analyzed for metabolite levels, including NEFA and β‐hydroxybutyrate (BHB), to assess the energy status of the cows. NEFA and BHB concentrations were quantified using the NEFA assay kit (FA115; Randox laboratory limited, United Kingdom) and the Ranbut D‐3‐hydroxybutrate with BHBA kit (RB1007; Randox laboratory limited, United Kingdom), respectively, following the manufacturer's protocols. Analyses were performed using Cobas Mira machine (LaborLab, Brazil). To confirm the presence of an active CL, serum progesterone concentrations were measured from samples collected during UF collection (Day 5 post‐AI) at 30 and 60 DPC. CL diameter was also evaluated using transrectal ultrasonography. Progesterone levels were assessed using a chemiluminescence kit (Progesterone III, Roche, Switzerland, cat. # 07092539), with an intra‐assay coefficient of variation of 13.7%.

### 2.3 In Vivo UF Collection

UF samples were collected 5 days post‐AI, corresponding to approximately 15 days after the initiation of the synchronization protocol at 30 and 60 DPC. At the time of collection, blood samples were taken to evaluate progesterone levels and CL diameter for assessing CL functionality. The UF collection was performed under epidural anesthesia using 2% Lidocaine hydrochloride (Lidovet, Bravet, Brazil). Both uterine horns were flushed with 50 mL of dmPBS (Dulbecco's Phosphate Buffered Saline; REPRODUX, Brazil) using a Foley embryo transfer catheter (24FR; Medix, Brazil). The volumes of UF recovered were as follows (mean ± SD, in mL): 33.9 ± 4.2 for Low NEB 30 DPC; 38.8 ± 5.6 for High NEB 30 DPC; 32.7 ± 3.8 for Low NEB 60 DPC and 40.5 ± 3.3 for High NEB 60 DPC. Recovered UF was collected in a 60 mL syringe and transferred to a 50 mL plastic tube. Samples were transported on ice to the laboratory for further processing, including the isolation of small extracellular vesicles (sEVs) and UEpC, which began within 1–2 h of sample collection.

### 2.4 Separation of sEVs and Epithelial Uterine Cells From UF

sEVs from uterine fluid (UF‐EVs) and UEpC were collected from dairy cows with Low and High NEB at day 30 and 60 DPC. To isolate UF‐EVs, the UF underwent a series of centrifugation steps to remove live cells, cellular debris, and large vesicles: 300 x g for 10 min, 2000 x g for 10 min, and 16,500 x g for 30 min. After the first centrifugation, the EUC pellets were separated and stored at −80°C until further use. The supernatant was also stored at −80°C until further processing. For sEVs isolation, the UF supernatant was filtered through a 0.20 μm sterile syringe filter (PES membrane; Corning, United States of America) to remove residual large EVs. Subsequently, the filtered UF was centrifuged twice at 119,700 x g for 70 min at 4°C using an ultracentrifuge (Optima XE‐90 Ultracentrifuge; rotor 70 Ti; Beckman Coulter, United States of America). The supernatant was discarded, and the resulting sEVs pellets were resuspended in 30 μL of phosphate‐buffered saline (PBS) (1 × Ca^2+^/Mg^2+^ free PBS; 137 mM NaCl, 2.7 mM KCl, 10 mM Na_2_HPO4, 2 mM KH_2_PO_4_) for further analysis. The sEV samples were divided into two sets: One set was analyzed for sEVs size and concentration, and the other set was diluted in TRI Reagent BD (Molecular Research Center, Inc., United States of America) and stored at −80°C for RNA analysis.

### 2.5 Nanoparticle Tracking and Transmission Electron Microscopy (TEM) Analysis

The particle size and concentration of sEVs were measured using nanoparticle tracking analysis (NTA) on a NanoSight NS300 instrument (Malvern, United Kingdom). Samples were diluted at a ratio of 1:100 in 1 × Ca^2+^/Mg^2+^ free PBS before analysis. Each sample was analyzed by capturing five 60‐second images under controlled conditions (camera level: 12, detection threshold: 5, and temperature: 38.5°C. Data on particle size and concentration were processed using NanoSight NTA 3.4 Analytical Software (NTA 3.4 Build 3.4.003; Malvern, United Kingdom).

The morphology of the sEVs was characterized using TEM. sEV pellets obtained from 2 mL of UF via ultracentrifugation were fixed in 200 μL of a fixing solution containing 0.1 M cacodylate; 2% glutaraldehyde, and 2% paraformaldehyde at pH 7.2–7.4) for 2 h at room temperature. After fixation, 2 mL of PBS was added to the sample to wash the fixative solution. The samples were centrifuged once (119,700x g for 70 min at 4°C) to re‐pellet the sEVs. The pellets were then resuspended in 30 μL of 1% cacodylate buffer and placed onto copper grids for approximately 60 min to air‐dry at room temperature. Grids were stained with 2% uranyl acetate for 90 s. Excess stain was removed using filter paper, and the grids were allowed to dry before analysis using a TEM instrument (FEI Tecnai 20; LAB6 emission; 200 kV).

### 2.6 Flow Cytometry

Flow cytometry was used for the detection of sEV markers. For machine preparation, ultrapure water served as sheath fluid to minimize background. Isolated UF‐EVs were stained with the following antibodies as positive markers: PE‐conjugated mouse monoclonal Alix (sc‐53540; 1:50), FITC‐conjugated mouse monoclonal CD9 (AB18241; 1:5), FITC‐conjugated mouse monoclonal CD63 (AB18235; 1:5), mouse monoclonal Syntenin (sc‐515538; 1:10), and Alexa fluor 488 goat anti‐mouse polyclonal secondary antibody (A11001; 1:2000). As a marker to detect any contamination in the isolate, FITC‐conjugated mouse monoclonal Calnexin (sc‐23954; 1:50) was used. For samples preparation, the samples were individually incubated with the antibodies for at least 2 h at room temperature in a shaker. For Alix, Syntenin and Calnexin, before the incubation the sEVs samples were incubated with 0.001% Triton X‐100 (X100) solution for 15 min at room temperature. For Syntenin, Alexa fluor 488‐conjugated secondary antibody was added to the samples (100 μL) and incubated for further 1:30 h. After the incubation, samples were diluted in 200 µL PBS trice filtered and analyzed by Cytoflex (Beckman Coulter, United States of America). The flow cytometry instrument was optimized for nanoparticle detection by the violet SSC channel (V‐SSC) and the fluorophore PE. The gain for V‐SSC was 100, FITC 450 and PE 600. The threshold was set primarily for V‐SSC at 500 and secondarily for FITC at 600. Events/s were maintained around 2000, and the abortion rate less than 8%. Approximate size of the nanoparticles was determinate using a mixture of fluorescent Megamix‐Plus SSC and MegamixPlus FSC beads (BioCytex, France), which have different sizes (100, 160, 200, 240, 300, 500, 900 nm). Using the controls (unlabeled sEVs and negative samples), gating was organized so unlabeled particles and negative samples were not detected. The acquisition was programmed to occur slowly (10 μL/min) for 5 min/sample. Thus, the number of events within the set gates was used to determine the presence or absence of markers.

### 2.7 Epithelial Uterine Naïve Cells Collection and Culture

The estrous cycles of dairy heifers (*n* = 9) were synchronized using a progesterone‐releasing intravaginal device (IVD; Progestar, Biógenisis Bagó, Brazil, 0.96 g progesterone) combined with intramuscular injections of 2 mg EB (Betaproginn, Boehringer Ingelheim, Germany) and 500 μg prostaglandin F_2α_ analog (PGF_2α_; cloprostenol, Estron, Agener União Saúde Animal, Brazil). The IVD was removed after 7 days, followed by administration of 1 mg estradiol cypionate (EC; E.C.P., Zoetis, United States of America) and 500 μg PGF_2α_ w. Cows observed in estrus (*n* = 8) were included in the study. Four days post‐estrous, luminal epithelial cells were collected in vivo from the ipsilateral horn to the CL using a cytology brush (Cytobrush; Viamed Ltd., United Kingdom; Figure 1C) as described by Cardoso et al. (2017) and ROCHA et al. (2022). Brushes containing cells (*n* = 4 heifers) were transported to the laboratory in a transport medium consisting of Dulbecco's modified Eagle medium/nutrient mixture F12 Ham (DMEM/F‐12; cat. #D6421) supplemented with 10% fetal bovine serum (FBS; cat. #10270106 Gibco/ThermoFisher Scientific, United States of America), 3% penicillin‐streptomycin (Gibco/ThermoFisher Scientific, United States of America, cat. # 15240112), and 2% amphotericin B (Gibco/ThermoFisher Scientific, United States of America, cat. # 15290026) at room temperature. Upon arrival at the laboratory, brushes were rinsed to detach adhered cells, and the resulting suspension was pelleted by centrifugation (200x g, 5 min, 24°C) as described by Rocha et al. (2022). The pellet was treated with a hyperosmotic lysis buffer (1 mM EDTA, 150 mM NH_4_Cl and 100 mM NaHCO_3_) for 1 min to lysed erythrocytes, followed by neutralization with the transport medium. After washing twice with medium, cells were pelleted and resuspended in pre‐warmed cell culture medium. The cell culture consisted of DMEM/F‐12, supplemented with 10% FBS, 2% penicillin/streptomycin, and 1% fungizone. Cells were plated in 6‐well plates (one well per heifer) and cultured at 38.5°C in a humidified 5% CO_2_ atmosphere. The culture medium was replaced every 2 days until the cells reached 90% confluence. Once confluent, cells were transferred to P175 flasks (SPL Life Sciences, Korea, #70175) for further propagation.

Primary monolayers at 90% confluence were washed with PBS (Ca^2+^ and Mg^2+^ free) and dispersed using TrypLE Express (Gibco/ThermoFisher Scientific, United States of America, cat. # 12604‐021) for 6 min. The reaction was stopped by adding culture medium, and cells were pelleted 200x g for 5 min at 24°C. Epithelial uterine naïve cells were seeded at a density of 1 × 104 viable cells per well in 24‐well tissue culture plates (Corning Incorporated, Costar 3524, United States of America) using 1 mL of culture medium containing EVs‐depleted FBS. Treatments were initiated 24 h after seeding. All experiments were conducted in triplicate using cells derived from three different heifers, with each heifer representing a biological replicate.

### 2.8 EVs Supplementation During Epithelial Uterine Naïve Cells Culture

The effects of UF‐EVs on bovine epithelial uterine naïve cells were evaluated following supplementation of the culture medium with UF‐EVs from cows in different metabolic statuses and postpartum conditions. Pooled UF‐EVs were isolated from 1 mL of UF per cow, collected from animals categorized based on their NEB status (Low or High) and DPC (30 or 60 DPC) as follows:30 DPC (Low NEB = 3; High NEB = 4) and 60 DPC (Low NEB = 3; High NEB = 3). The UF‐EVs were resuspended in an equal volume of DMEM/F‐12 medium supplemented with 2% penicillin/streptomycin and 1% fungizone. At 30 DPC, UF‐EVs from the Low NEB group were resuspended in 3 mL of medium, while those from the High NEB group were resuspended in 4 mL. At 60 DPC, UF‐EVs from both groups were resuspended in 3 mL of medium. The particle concentrations, measured by NTA, were as follows: 1.55 × 1010 ± 3.41 × 108 particles/mL for UF‐EVs from animals with Low NEB at 30 DPC; 5.36 × 109 ± 1.67 × 108 particles/mL for UF‐EVs from animals with High NEB at 30 DPC; 1.12 × 1010 ± 3.04 × 108 particles/mL for UF‐EVs from animals with Low NEB at 60 DPC and; 5.43 × 109 ± 2.22 × 108 particles/mL for UF‐EVs from animals with High NEB at 60 DPC. Twenty‐4 h after seeding, the culture medium containing EV‐depleted FBS was removed, and cells were washed twice with PBS. The bovine epithelial uterine naïve cells (*n* = 3, with each heifer considered a biological replicate) were then incubated for 6 h in one of the following treatment groups: (1) Control group: DMEM/F‐12 supplemented with 2% penicillin/streptomycin, and 1% fungizone, without UF‐EVs; (2) Low NEB, 30 DPC: medium supplemented with UF‐EVs from low NEB animals at 30 DPC; (3) Low NEB, 60 DPC: medium supplemented with UF‐EVs from low NEB animals at 60 DPC; (4) High NEB, 30 DPC: medium supplemented with UF‐EVs from high NEB animals at 30 DPC; and (5) High NEB, 60 DPC: medium supplemented with UF‐EVs from high NEB animals at 60 DPC. After 6 h of treatment, cells were collected for RNA extraction and immunofluorescence detection.

### 2.9 Immunofluorescence Detection of Cytokeratin

Bovine epithelial uterine naïve cells were cultured on coverslips placed in 24‐well tissue culture plates at a density of 1 × 105 cells per well. After 24 h of culture, the cells were treated for 6 h as described above. Briefly, cells were fixed with 4% paraformaldehyde for 15 min and then permeabilized with D‐PBS + 1% Triton X‐100 for 30 min. Blocking was performed for 1 h at room temperature in a solution containing D‐PBS, 0.3% Triton X‐100% and 1% BSA. Cells were then incubated overnight at 4°C with a primary mouse Cytokeratin antibody (epithelial cell marker; C2931 diluted 1:1000 in blocking solution). Negative controls included cells incubated without primary antibodies but treated with secondary antibodies. After overnight incubation, cells were washed four times for 5 min each with D‐PBS and then incubated with a secondary antibody (Alexa Fluor 488‐conjugate goat anti‐mouse IgG; Life Technologies, United States of America; cat. # A‐11029) for 1 h. Nuclei were stained with 10 μg/mL Hoechst for 15 min. Cells were then washed three times for 10 min each with D‐PBS and coverslips mounted onto microscope slides using Prolong Gold Antifade Mountant (Life Technologies, United States of America; cat. #: P36935). Images were captured using a Thunder 3D Imager microscope (DM18; Leica, Germany) under identical settings for all samples within the same experiment. Fluorescent intensity (average mean gray value) was measured for each channel by manually outlining nuclei and adjusting against the background. All samples for a given experiment were processed simultaneously, and imaging was conducted under consistent laser power settings.

### 2.10 Total RNA Extraction of sEVs, UEpCs, and Epithelial Uterine Naïve Cells

Total RNA, including mRNA and miRNAs, from UF‐EVs, UEpC and epithelial uterine naïve cells after 6 h treatment with UF‐EVs was extracted using miRNeasy Mini Kit (QIAGEN, Hilden, Germany), in accordance with the manufacturer's instruction. To avoid DNA contamination, the RNA was treated with RNase‐Free DNase Set (QIAGEN, Hilden, Germany; cat #79254) according to the manufacturer's instructions. RNA quality and concentration were analyzed using spectrometry (NanoDrop 2000; Thermo Fisher Scientific, United States of America). For RNA Sequencing (RNA‐Seq), the RNA quality was also assessed using a Bioanalyzer.

### 2.11 Reverse Transcription and Quantitative Reverse Transcription‐Polymerase Chain Reaction (RT‐PCR) Analysis for miRNAs and mRNAs

Total RNA isolated from both UF‐EVs and epithelial uterine naïve cells cultured with UF‐EVs was used to investigate miRNA expression. To evaluate the relative levels of 382 bovine miRNAs from UF‐EVs and epithelial uterine naïve cells after 6 h of treatment with UF‐EVs, a Poly‐A tail was first added to the RNA. The reaction mix contained: 10x Poly(A) Polymerase Reaction Buffer (New England BioLabs, United States of America; cat #B0276S); 10 mM Adenosine‐5’‐Triphosphate (ATP; New England BioLabs, United States of America; cat #B0756A); 5000 units/mL *Escherichia coli* Poly(A) Polymerase (New England BioLabs, United States of America; cat #B0276S) and 10 μM Oligo‐dT adapter primer (5′‐GCATAGACCTGAATGGCGGTAAGGGTGTGGTAGGCGAGACATTTTTTTTTTTTTTTTTTTT ‐ 3′). A total of 200 ng RNA was incubated with the reaction mix (final volume: 5 μL) at 37°C for 60 min, followed by 70°C for 5 min. RT‐PCR was then performed using ReadyScript cDNA Synthesis Min (RDRT‐100RXN) at 25°C for 5 min, 42°C for 30 min, and 85°C for 5 min. The reaction was prepared to a final volume of 10 μL.

Quantitative RT‐PCR was performed using GoTaq qPCR Master Mix (Promega, United States of America), Universal Primer (10 μM; 5’‐ GCATAGACCTGAATGGCGGTA – 3’), 10 μL of cDNA in the mix, and 1 μL of specific forward primer (10 μM; Table S1). The reaction volume was completed to 6 μL per well. Reactions were analyzed in 384‐well plates using a QuantStudio 6 Flex (Applied Biosystems, United States of America), with the following PCR cycle conditions: 95°C for 15 min, 45 cycles of 94°C for 15 s, 55°C for 30 s, and 70°C for 30 s. A melting curve analysis followed to confirm the amplification of specific cDNA products. Reactions with Ct values > 37 or multiple peaks were excluded. The Ct values were normalized to the geometric mean of miR‐99b, RNU43 snoRNA, and Hm/Ms/Rt U1 snRNA. The relative expression was calculated using the Ct method and the data transformed by 2^−ΔCt^ for graphical representation of relative transcript levels (Schmittgen and Livak 2008). The heatmap was generated using MetaboAnalyst 6.0 to illustrate the relative levels of transcript expression. Bioinformatic analyses were performed using miRWalk software version 3.0 to identify pathways modulated by miRNAs that were differentially expressed between Low and High NEB groups at 30 and 60 DPC. Pathways with a BH < 0.05 were considered enriched. We considered miRNAs as exclusive if they were present in all samples from one group and absent in all samples from the other group.

To confirm the presence of bovine epithelial uterine naïve cells in the culture, part of the extracted mRNA after 6 h of treatment with UF‐EVs was reverse‐transcribed into cDNA using 30 ng of total RNA per gene with the High‐Capacity cDNA Reverse Transcription Kit (Thermo Fisher Scientific, United States of America). Real‐time‐PCR was conducted to measure the relative abundance of *KRT18* transcripts (Sponchiado et al. 2020) using GoTaq qPCR Master Mix (Promega, United States of America) and QuantStudio 6 Flex (Applied Biosystems, United States of America). The RT‐qPCR reactions were performed in duplicate, with 5 μL SYBR Green master mix, 1.5 μL of forward and reverse primers (Final concentration: 0.5 μM), and 3.5 μL nuclease‐free water with cDNA template (30 ng of total RNA per gene). The PCR cycle conditions were 95°C for 10 min, followed by 45 cycles of 95°C for 15 s, and 60°C for 60 s. Amplification of a single cDNA product was confirmed by melt curve analysis. The relative expression was calculated using the Ct method and normalized using the geometric mean of the reference genes *PPIA* and *ACTB* (Rocha et al. 2022). The relative transcript levels were then represented graphically using 2^−ΔCt^, as described by SCHMITTGEN; LIVAK (2008).

### 2.12 cDNA Synthesis, RNA Library Preparation, and Sequencing

The cDNA synthesis and amplification of UEpC from animals with Low and High NEB at 30 and 60 DPC were performed using the SMART‐Seq HT Kit (Takara Bio Inc, Japan), following the manufacturer's recommendations. After synthesis, the cDNA was purified using AMPure XP Beads (Beckman Coulter, United States of America). The concentration of cDNA was determined using the Qubit dsDNA High Sensitivity (ThermoFisher Scientific, United States of America), and its length was assessed using the Bioanalyzer High Sensitivity DNA Kit (Agilent, United Sates of America). Libraries were prepared using the Nextera XT DNA Library Prep (Illumina, United Sates of America), as recommended by manufacturer. Before sequencing, the libraries were quantified with Qubit, and their fragment size was analyzed on the Bioanalyzer. RNA‐Seq was performed with 75 bp single‐end reads on the NextSeq. 550 (Illumina, United States of America). The entire cDNA synthesis, library preparation, and sequencing were carried out at the Multiuser Laboratory of Cellular and Molecular at the Federal University of São Carlos (UFSCar).

The quality of the sequencing reads was assessed using FASTQC software (http://www.bioinformatics.babraham. ac. uk/projects/fastqc). The reads were mapped using the STAR (DOBIN et al. 2013), with identification and quantification performed using the ARS‐UCD1.2) reference genome from Ensembl and NCBI. Gene counts were calculated using the featureCounts function in the Rsuberead package (Liao et al. 2014, 2019). Genes were considered expressed if they had more than 10 read counts in all samples from at least one group. Differential gene expression analysis was conducted using the DESEQ. 2 R package (Love et al. 2014), with adjusted *p* values < 0.10 and absolute log2 fold changes > 0.5 considered significant. Gene ontology analysis was performed using the ClusterProfiler (Yu et al. 2012). The results were visualized using the R software, with a focus on the classification, intensity, and differential expression between the groups.

### 2.13 Statistical Analysis

Continuous data and residuals were tested for normality using the Shapiro–Wilk test. NEFA and BHB concentrations were analyzed using mixed‐effects models for repeated measures, with group, day, and the interaction between group and day included as fixed factors, and cow treated as a random subject effect. Various covariance structures were evaluated for each model, and the structure with the lowest Akaike Information Criterion was selected. NEFA values were normalized using the sinh‐arcsinh (SHASH) transformation. Other continuous data, such as the size and concentration of sEVs, as well as mRNA and miRNA expression levels, were compared between groups (Low vs. High NEB) at specific time points using Student's *t*‐test. All analyses, except for RNA‐Seq analysis, were performed using JMP 17 software (SAS Institute Inc., United States of America). A *p* value < 0.05 was considered statistically significant.

## 3 Results

### 3.1 Characterization of Dairy Cows With Low and High NEB

**Figure 2:**
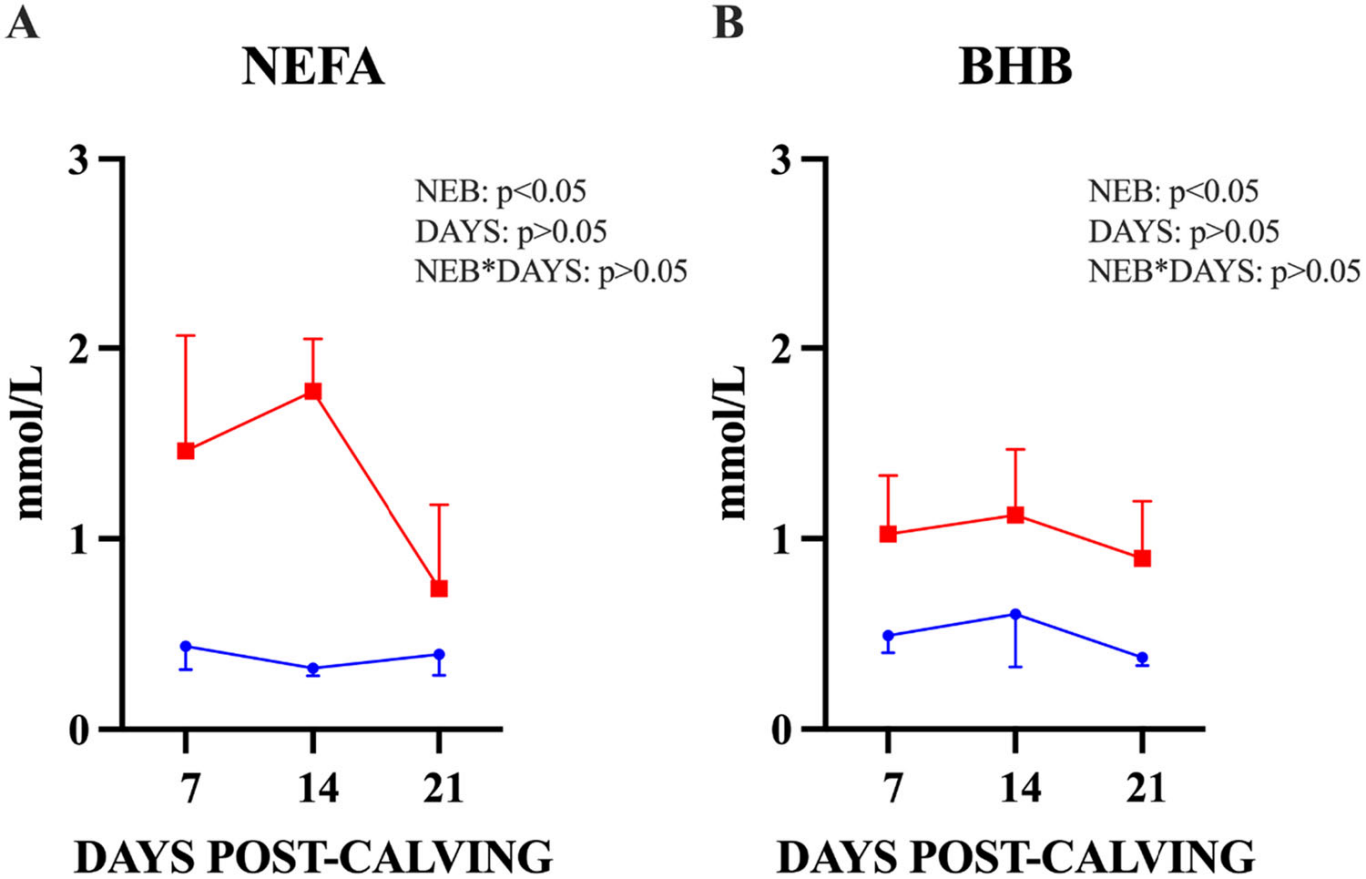
NEFA and BHB concentration in the serum. (A) Blood samples to evaluate NEFA were evaluated once a week during the 3 weeks post‐calving (DPC; *p* < 0.05). (B) BHB were evaluated once a week during the 3 weeks post‐calving (*p* < 0.05). *n* = 7 total; Low NEB = 3 (blue line); High NEB = 4 (red line). BHB, β‐hydroxybutyrate; NEFA, nonesterified fatty acids.

The intensity of NEB in dairy cows was assessed through weekly blood metabolite analyses, starting on the week post‐calving and continuing until the third week post‐calving. NEFA concentrations at 14 DPC were used to classify cows based on thresholds described in the literature (Fenwick et al. 2008; Girard et al. 2015; Ospina et al. 2010b). Cows were classified as being in Low NEB when NEFA concentrations were < 1.0 mmol/L and in High NEB when NEFA concentrations were ≥ 1.0 mmol/L, at 14 DPC. As shown in Figure 2A, NEFA concentrations increased significantly over time in High NEB animals during the 3 weeks post‐calving (Low NEB 0.38 ± 0.05 mmol/L, High NEB 1.43 ± 0.28 mmol/L; *p* value < 0.05). We also measured the BHB concentrations (BHB: Low NEB 0.49 ± 0.09 mmol/L, High NEB 0.98 ± 0.18 mmol/L; Figure 2B). Additional metabolic parameters (Figure S1) and body condition scores (BCS; 7 DPC: Low NEB = 3.87 ± 0.53, High NEB = 3.68 ± 0.12; 14 DPC: Low NEB = 3.58 ± 0.14, High NEB = 3.68 ± 0.37; 21 DPC: Low NEB = 3.5 ± 0.25, High NEB = 3.56 ± 0.23) showed no significant differences between groups. Rectal temperatures were measured during sampling, and cows with temperatures above 38.5°C were excluded. A carefully selected subset of animals (*n* = 7) was included based on the absence of uterine or systemic diseases and the absence of any metabolic alterations, apart from elevated NEFA levels, during the first 3 weeks postpartum. NEFA and BHB concentrations were also measured during the UF collection at 30 (NEFA: Low NEB = 0.22 ± 0.02, High NEB = 0.31 ± 0.14; BHB: Low NEB = 0.31 ± 0.07, High NEB = 1.00 ± 0.28) and 60 DPC (NEFA: Low NEB = 0.19 ± 0.07, High NEB = 0.84 ± 0.73; BHB: Low NEB = 0.36 ± 0.08, High NEB = 0.58 ± 0.31). No significant differences were observed between the groups (*p* > 0.05).

Before UF collection, all animals underwent estrous cycle synchronization and were at the same cycle stage. At the time of collection, progesterone concentrations and CL diameters were assessed. Progesterone concentrations were similar between groups at 30 DPC (Low NEB: 2.52 ± 0.63 ng/mL; High NEB: 2.72 ± 1.63 ng/mL; *p* > 0.05) and at 60 DPC (Low NEB: 5.58 ± 4.32 ng/mL; High NEB: 1.7 ± 0.96 ng/mL; *p* > 0.05). The CL diameters were as follows: at 30 DPC, Low NEB 2.85 ± 0.96 cm^2^ versus High NEB 6.54 ± 8.4 cm^2^ (*p* > 0.05); at 60 DPC, Low NEB 2.17 ± 1.12 cm^2^ versus High NEB 2.02 ± 0.21 cm^2^ (*p* > 0.05).

### 3.2 Size and Concentration of EVs in UF Are Unaffected by NEB at 30 and 60 DPC

**Figure 3:**
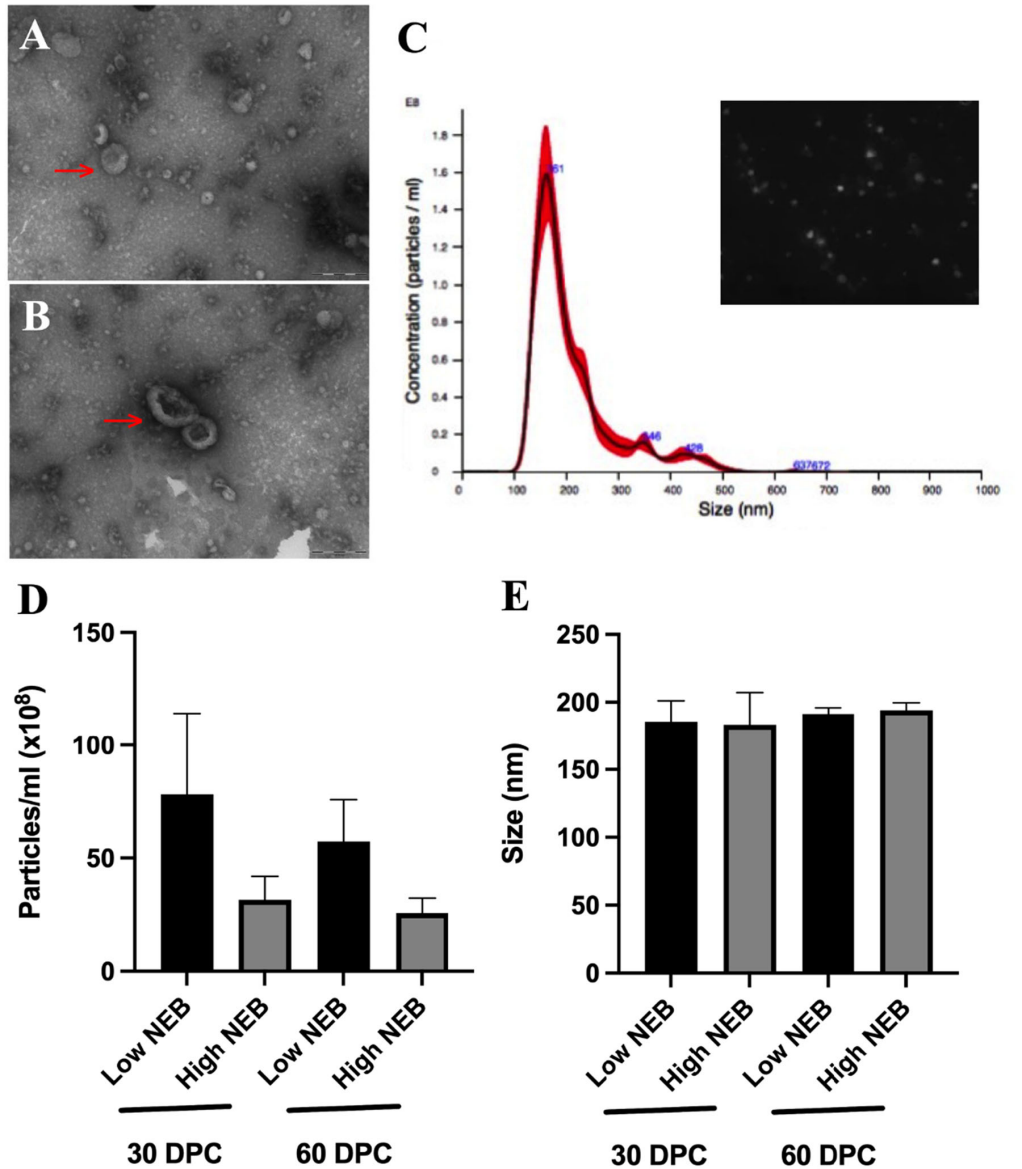
Small extracellular vesicles characterization and analysis. (A, B) Transmission electron microscopy images of sEVs from uterine fluid of dairy cows with Low and High Negative Energy Balance (NEB) at 30 and 60 days post‐calving. The magnification of both electron microscopy images was 200,000×. (C) Nanoparticle tracking analysis characterization of size and concentration profiles of UF‐EVs present in the uterine fluid (example image). (D) sEVs concentration presented in the uterine fluid of dairy cows with Low and High NEB at 30 DPC (Low NEB = 3; High NEB = 4) and 60 DPC (Low NEB = 3; High NEB = 3). (E) sEVs size presented in the uterine fluid of dairy cows with Low and High NEB at 30 DPC (Low NEB = 3; High NEB = 4) and 60 DPC (Low NEB = 3; High NEB = 3). Bars represent the group means, and error bars represent the standard error of the mean (SEM). DPC, days post‐calving; NEB, negative energy balance; sEVs, small extracellular vesicles; UF‐EVs, extracellular vesicles from uterine fluid.

The presence of sEVs in the UF was analyzed using TEM. TEM images confirmed the morphology and diameter of sEVs consistent with previous literature descriptions (Figure 3A,B). The size and concentration of UF‐derived sEVs (UF‐EVs) from Low and High NEB cows at 30‐ and 60‐DPC were characterized using NTA (Figure 3C). At 30 DPC, no significant differences were observed in particle concentration (Low NEB: 78.2 × 108 ± 35.9 × 108 particles/mL; High NEB: 31.6 × 108 ± 10.3 × 108 particles/mL) or mode size (Low NEB: 185.7 ± 15.3 nm; High NEB: 183.4 ± 23.7 nm; Figure 3D). Similarly, at 60 DPC, particle concentration (Low NEB: 57.3 × 108 ± 18.4 × 108 particles/mL; High NEB: 25.7 × 108 ± 6.5 × 108 particles/mL) and mode size (Low NEB: 189.52 ± 5.6 nm; High NEB: 192.7 ± 4.2 nm; Figure 3E) did not differ significantly between the groups. These results suggest that NEB does not influence the concentration or size of sEVs in UF at either 30 or 60 DPC.

**Figure 4:**
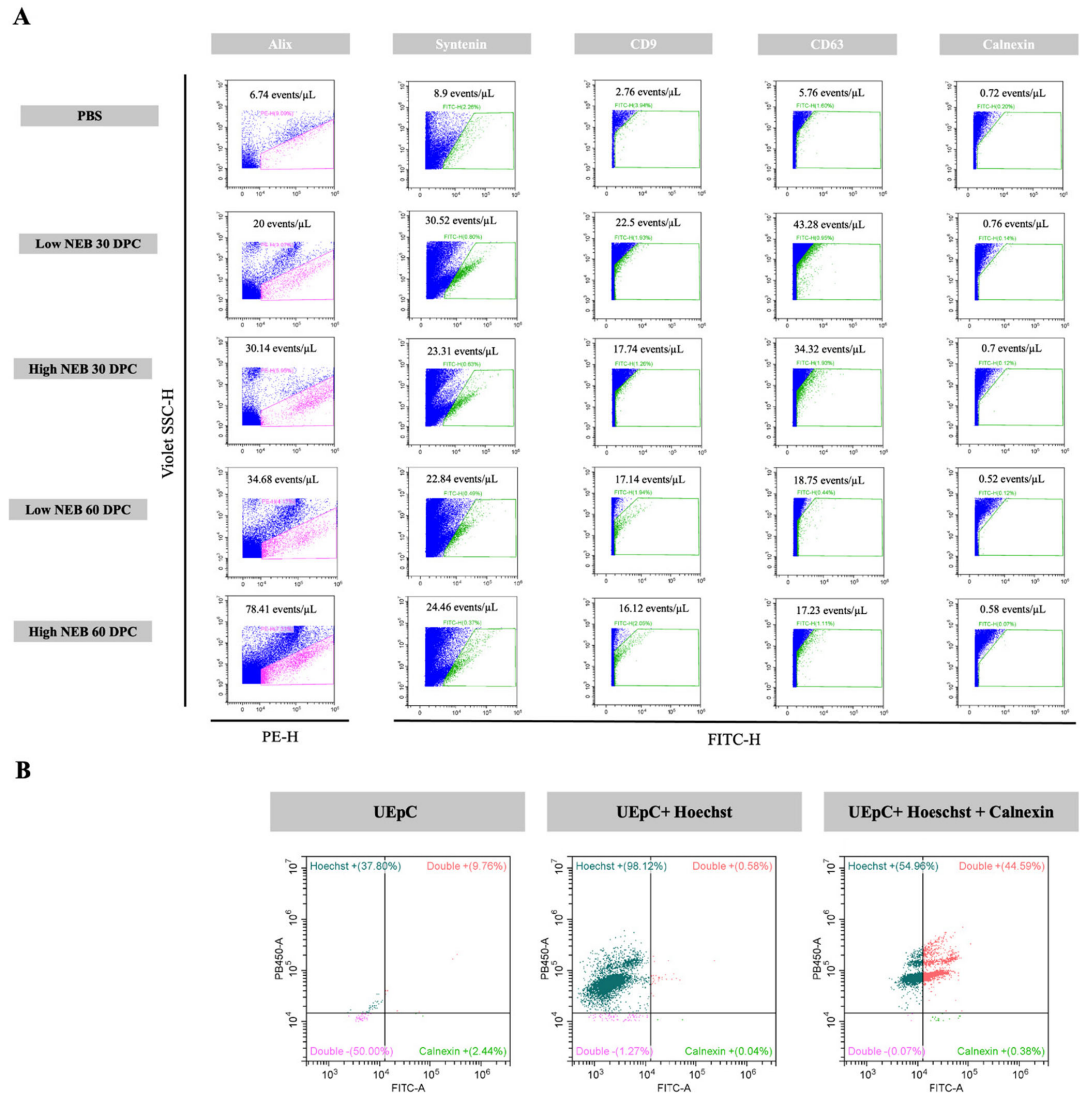
Characterization of small extracellular vesicles (sEVs) by flow cytometry. (A) Panels illustrating the gating strategy and detection of surface and cytoplasmic markers Alix, Syntenin, CD9, CD63, and Calnexin in sEVs isolated from uterine fluid of dairy cows experiencing Low and High Negative Energy Balance (NEB) at 30 and 60 days post‐calving (DPC). (B) Panels showing Calnexin detection in uterine epithelial cells. The X‐axis represents the fluorescence intensity of the corresponding fluorophore (PE‐H or FITC‐H), while the Y‐axis (Violet SSC‐H) displays the side scatter signal in the violet laser. Gates were defined based on the negative control (PBS + antibody) and subsequently applied to the sEV samples labeled with specific antibodies. The number of events per microliter and the percentage of positive events for each marker are indicated in each plot. DPC, days post‐calving; NEB, negative energy balance; sEVs, small extracellular vesicles; UEpC, uterine epithelial cells.

Nano‐flow cytometry analysis confirmed the presence of specific surface markers characteristic of sEVs in all experimental groups (Low and High NEB at 30 and 60 DPC). The analysis showed that the samples were positive for Alix, Syntenin, CD9, and CD63, and negative for Calnexin (Figure 4A). PBS was used as the negative control. The concentration of positive events/µL for Alix was 50.20 in Low NEB 30 DPC, 151.29 in High NEB 30 DPC, 30.10 in Low NEB 60 DPC, 101.51 in High NEB 60 DPC, and 6.74 in the PBS. For Syntenin, positive events/µL were 23.19 in Low NEB 30 DPC, 11.54 in High NEB 30 DPC, 15.98 in Low NEB 60 DPC, 14.65 in High NEB 60 DPC, and 8.90 in the PBS. Regarding CD9, values were 12.43 in Low NEB 30 DPC, 12.36 in High NEB 30 DPC, 10.79 in Low NEB 60 DPC, 8.75 in High NEB 60 DPC, and 2.76 in the PBS, respectively. For CD63, the number of positive events/µL was 40.17 in Low NEB 30 DPC, 23.13 in High NEB 30 DPC, 16.80 in Low NEB 60 DPC, 23.35 in High NEB 60 DPC, and 5.76 in the PBS. These results confirm the presence of sEVs in the uterine flushings. Calnexin, a negative marker for sEVs, was used to verify the absence of cellular contaminants in the isolated vesicles (Figure 4B). As expected, all four groups exhibited markedly lower numbers of positive events/µL compared to the uterine cell lysate used as a positive control (Table S2).

### 3.3 MiRNA Contents in UF‐EVs Differ Between Dairy Cows With Low and High NEB at 30 and 60 DPC

**Figure 5:**
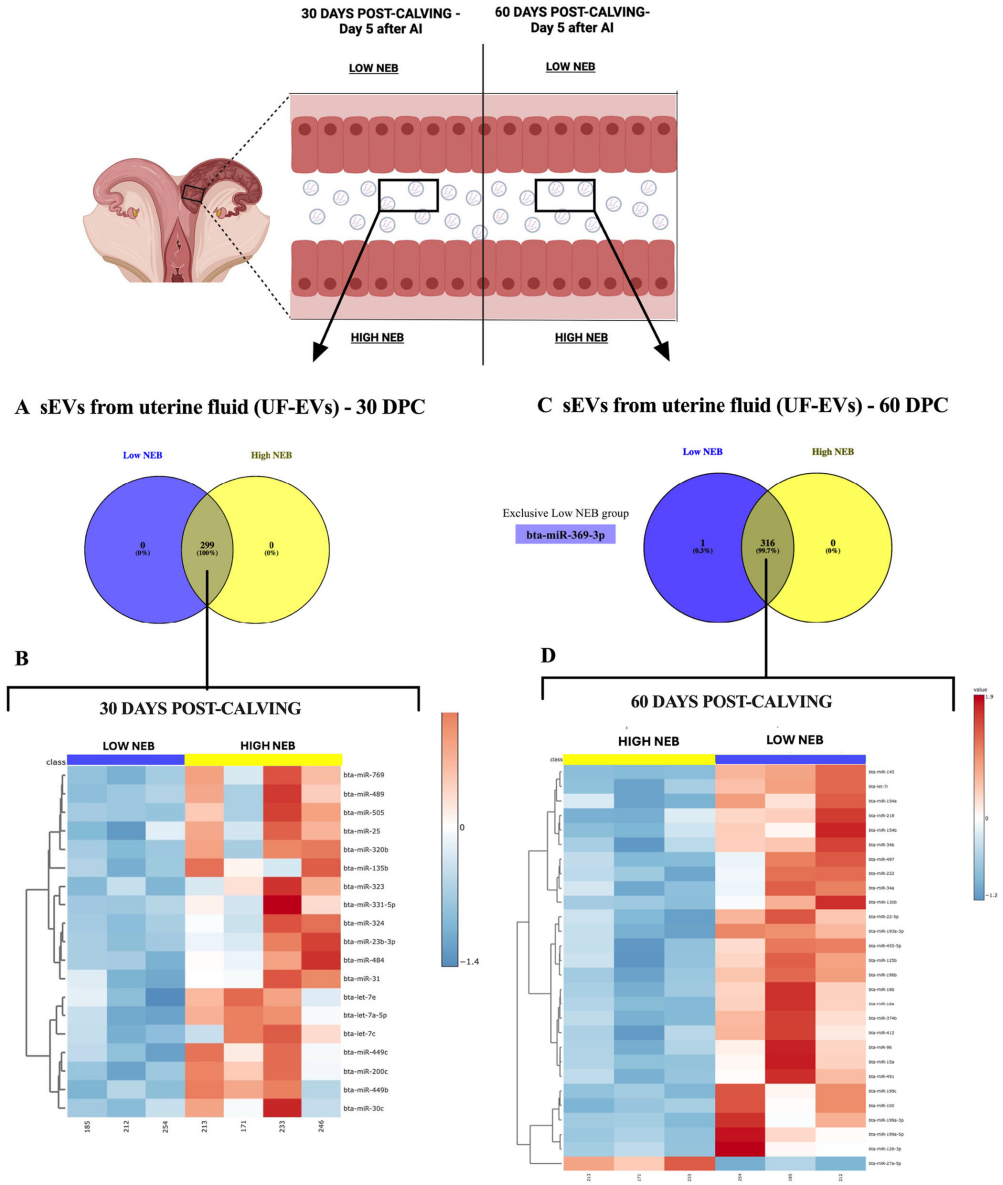
UF‐EVs miRNA profile from dairy cows at 30 and 60 DPC. MiRNA contents present in sEVs isolated from uterine fluid (UF‐EVs) of dairy cows with Low and High NEB at 30‐ and 60‐day post‐calving. (A) Venn diagram demonstrating the distribution of the 299 miRNAs present in UF‐EVs at 30 DPC. (B) Heatmap demonstrating that 19 miRNAs were upregulated in UF‐EVs from dairy cows with High NEB (red color). (C) Venn diagram demonstrating the distribution of the 317 miRNAs present in UF‐EVs at 60 DPC. (D) Heatmap demonstrating that 27 miRNAs were upregulated in UF‐EVs from dairy cows with Low NEB (red color), and one miRNA was upregulated in High NEB (red color). Data in the heatmap are *p* < 0.05. DPC, days post‐calving.

**Figure 6:**
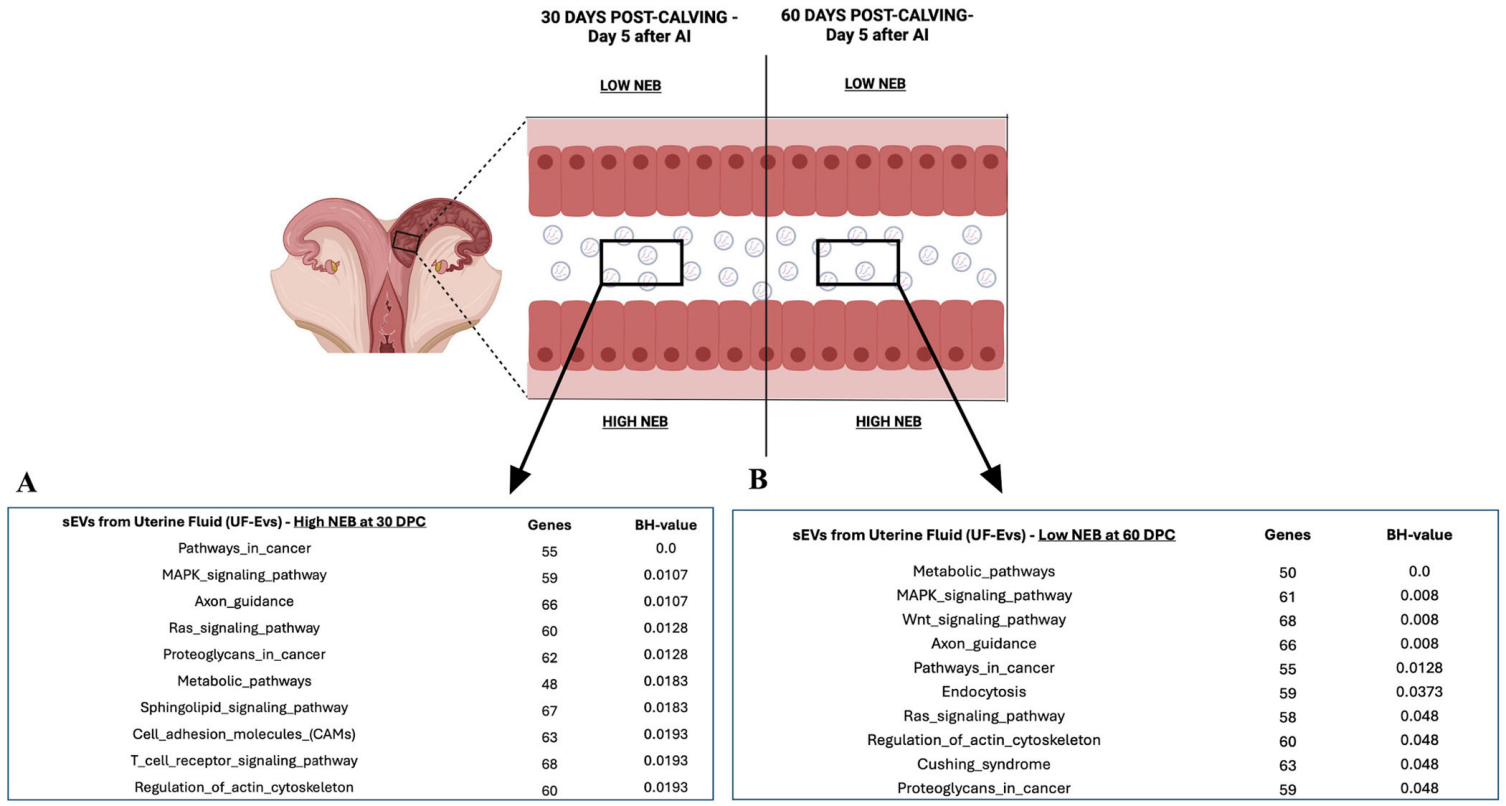
Enrichment analysis performed in miRWalk 3.0 software of predicted pathways modulated by upregulated miRNAs of sEVs miRNAs isolated from uterine fluid (UF‐EVs) of dairy cows with Low or High NEB at 30‐ and 60‐day post‐calving. (A) Top 10 predicted pathways regulated by 19 miRNAs upregulated in UF‐EVs from dairy cows with High NEB at 30 DPC. (B) Top 10 predicted pathways regulated by 27 miRNAs upregulated in UF‐EVs from dairy cows with Low NEB at 60 DPC. DPC, days post‐calving; NEB, negative energy balance; sEVs, small extracellular vesicles.

To investigate the impact of NEB on the uterine environment as it prepares to receive the embryo (5 days after AI), sEVs were isolated from the UF of dairy cows with synchronized estrous cycles and classified as Low or High NEB at 30 DPC (Low NEB = 3; High NEB = 4) and 60 DPC (Low NEB = 3; High NEB = 3). Total RNA from UF‐EVs was extracted and profiled for 382 bovine miRNAs using RT‐qPCR. At 30 DPC, 331 miRNAs were identified in UF‐EVs from Low NEB cows, and 307 were detected in High NEB cows (Table S3). Among these, 299 miRNAs were commonly detected between the groups (Figure 5A). Notably, 19 miRNAs were differently expressed and upregulated in UF‐EVs from High NEB cows compared to Low NEB cows (*p* < 0.05; Figure 5B) including bta‐let‐7a‐5p, bta‐let‐7c, bta‐let‐7e, bta‐miR‐135b, bta‐miR‐200c, bta‐miR‐23b‐3p, bta‐miR‐25, bta‐miR‐30c, bta‐miR‐31, bta‐miR‐331‐5p, bta‐miR‐320b, bta‐miR‐323, bta‐miR‐324, bta‐miR‐449c, bta‐miR‐449b, bta‐miR‐484, bta‐miR‐489, bta‐miR‐505, bta‐miR‐7690. Functional pathway analysis of the 19 upregulated miRNAs in UF‐EVs from High NEB cows was predicted to regulate 67 biological pathways. The top 10 most significant pathways (*p* < 0.05; Figure 6A) included Pathways in cancer, MAPK signaling pathway, Axon guidance, Ras signaling pathway, Proteoglycans in cancer, Metabolic pathways, Sphingolipid signaling pathway, Cell adhesion molecules, T cell receptor signaling pathway, regulation of actin cytoskeleton. These findings suggest that High NEB significantly induces increased levels of miRNAs in UF‐EVs, potentially downregulating key pathways involved in cellular signaling and uterine preparation for embryo implantation.

At 60 DPC, 339 miRNAs were identified in UF‐EVs from Low NEB cows and 328 in High NEB cows (Table S4). One miRNA (miR‐369‐3p) was uniquely detected in UF‐EVs from the Low NEB group, while 316 miRNAs were expressed in both groups (Figure 5C). Among these 316 shared miRNAs, 1 miRNA (miR‐27a‐5p) was upregulated in UF‐EVs from High NEB cows, whereas 27 miRNAs were upregulated in UF‐EVs from Low NEB cows (*p* < 0.05; Figure 5D). The miRNAs upregulated in the Low NEB group included: bta‐let‐7i, bta‐miR‐100, bta‐miR‐125b, bta‐miR‐126‐3p, bta‐miR‐130b, bta‐miR‐145, bta‐miR‐154a, bta‐miR‐154b, bta‐miR‐15a, bta‐miR‐16a, bta‐miR‐16b, bta‐miR‐193a‐3p, bta‐miR‐196b, bta‐miR‐199a‐3p, bta‐miR‐199a‐5p, bta‐miR‐199c, bta‐miR‐22‐5p, bta‐miR‐222, bta‐miR‐218, bta‐miR‐27a‐5p, bta‐miR‐34a, bta‐miR‐34a, bta‐miR‐34b, bta‐miR‐374b, bta‐miR‐412, bta‐miR‐455‐5p, bta‐miR‐497, bta‐miR‐491, bta‐miR‐96. Biological pathway analysis of the upregulated miRNAs in Low NEB revealed 10 predicted pathways at 60 DPC (*p* < 0.05; Figure 6B), including Metabolic pathways, MAPK signaling pathway, Wnt signaling pathway, Axon guidance, Pathways in cancer, Endocytosis, Ras signaling pathway, Regulation of actin cytoskeleton, Cushing syndrome, Proteoglycans in cancer. These results demonstrate that Low NEB induces an increase in miRNA contents in UF‐EVs. At 30 DPC, more miRNAs were upregulated in UF‐EVs from High NEB cows, whereas at 60 DPC, a greater number of miRNAs were upregulated in UF‐EVs from Low NEB cows. This suggests distinct mechanisms underlying the uterine response to NEB at different time points.

### 3.4 NEB Intensity and Post‐Calving Period Alter the Molecular Profile of UEpCs

**Figure 7:**
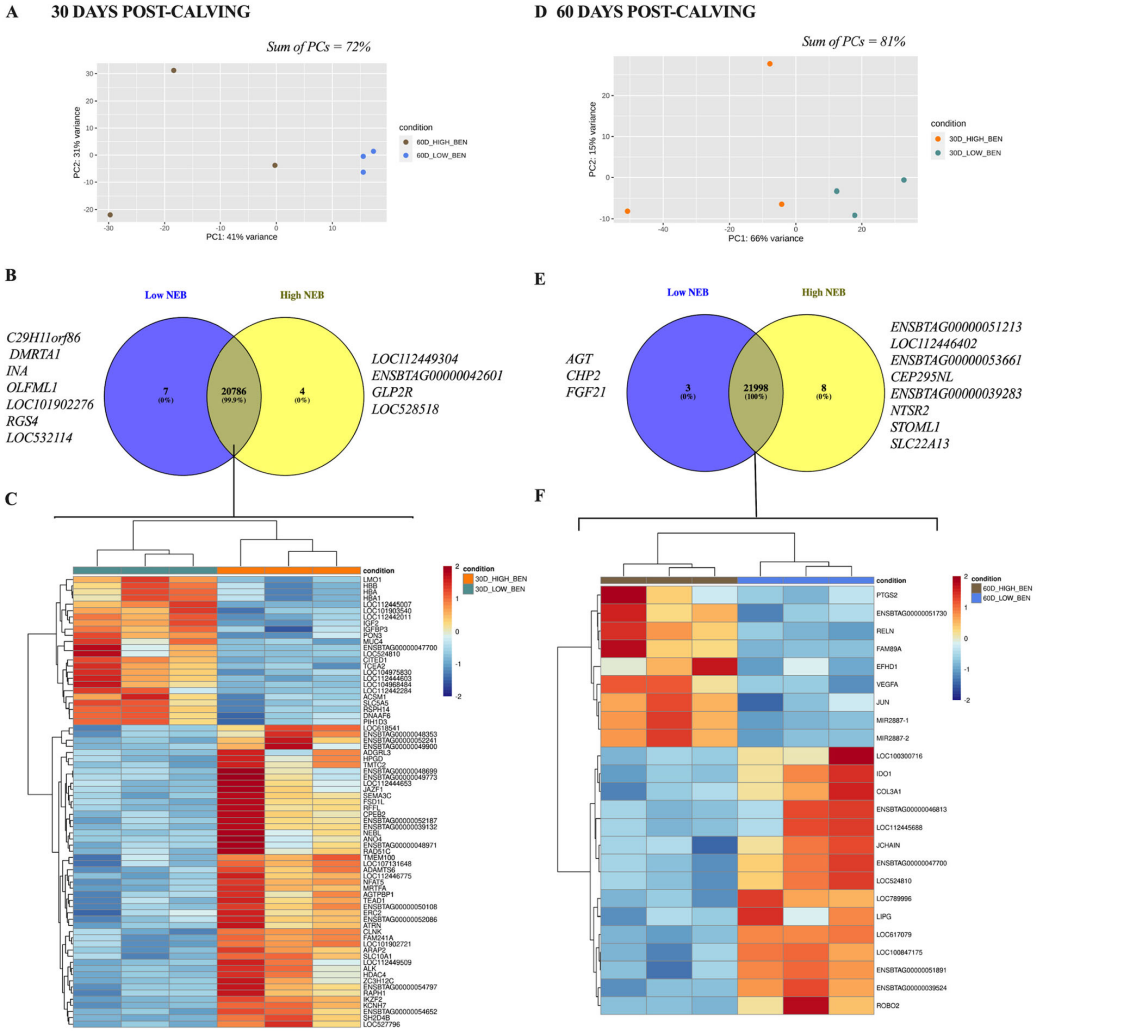
RNA profile of uterine epithelial cells from dairy cows that presented Low or High NEB at 30‐ and 60‐day post‐calving (DPC). (A) PCA represents the variation in RNAs in epithelial uterine cells at 30 days post‐calving. (B) Venn diagram of RNA‐Seq data from uterine epithelial cells collected at 30 DPC from dairy cows that presented Low or High NEB. (C) Heatmap representing the variation of differentially expressed genes (DEGs) between cells collected from dairy cows at 30 DPC. (D) PCA representing the variation in RNAs in uterine epithelial cells at 60 days post‐calving. (E) Venn diagram of RNA‐Seq data from uterine epithelial cells collected at 60 DPC from dairy cows that presented Low or High NEB. (F) Heatmap representing the variation of differentially expressed genes (DEGs) between cells collected from dairy cows at 60 DPC. Color scales vary from red (upregulated genes) to blue (downregulated genes). DEGs were either upregulated or downregulated when the *p*‐adjusted was < 0.10 and log2 fold change > 0.5.

To investigate how NEB intensity affects the RNA profile and associated biological process, we conducted a transcriptomic analysis of the UEpC at 30 and 60 DPC (Figure 7). The PCA analysis revealed distinct RNA profiles between the groups at both 30 DPC (Figure 7A) and 60 DPC (Figure 7D). At 30 DPC, we identified 7 transcripts (*C29H11orf86*, *DMRTA1*, *INA*, *OLFML1*, *LOC101902276*, *RGS4*, *LOC532114*) exclusive to the Low NEB, 4 transcripts (*LOC112449304*, *ENSBTAG00000042601*, *GLP2R*, *LOC528518*) exclusive to High NEB group, and 20.786 transcripts shared between the two groups (Figure 7B). By 60 DPC, 3 transcripts (*AGT*, *CHP2*, *FGF21*) were exclusive to the Low NEB group, 8 transcripts (*ENSBTAG00000051213*, *LOC112446402*, *ENSBTAG00000053661*, *CEP295NL*, *ENSBTAG00000039283*, *NTSR2*, *STOML1*, *SLC22A13*) were exclusive to the High NEB group, and 21.998 were commonly expressed between the groups (Figure 7E). Exclusive transcripts were defined as those detected in all samples from one group but absent in all samples from the other group. Analysis of differentially expressed genes (DEGs) showed that at 30 DPC, there were 73 DEGs between the groups, with 24 upregulated in the Low NEB group and 49 upregulated in the High NEB group (Figure 7C). At 60 DPC, 24 DEGs were identified, including 15 upregulated in the Low NEB group and 9 upregulated in the High NEB group (Figure 7F). These results align with the miRNAs analysis, indicating that High NEB cows exhibit a larger number of DEGs at 30 DPC, while Low NEB cows display a greater number of DEGs at 60 DPC.

Functional enrichment analysis of genes exclusively or upregulated in UEpC revealed that biological processes in these cells are differentially modulated depending on NEB intensity and DPC. At 30 DPC, genes that were exclusive or upregulated in UEpC from dairy cows with Low NEB at 30 DPC were associated with cell differentiation and proliferation, cell viability, and fatty acid metabolism. In contrast, genes exclusive or upregulated in UEpC from dairy cows with High NEB at 30 DPC were linked to nutrient absorption, inflammatory responses, reduced embryo development, and metabolic pathways. By 60 DPC, Low NEB cows exhibited gene activity associated with reduced lipid accumulation, blood pressure regulation, protection of embryos from maternal immune responses, conceptus development, and extracellular matrix organization. However, in High NEB cows, the gene activity at 60 DPC suggested ongoing recovery from metabolic stress, with exclusive or upregulated genes predicted to regulate metabolic processes and cell proliferation pathways. These results highlight the significant impact of NEB intensity on the RNA profile of uterine cells, suggesting that the uterine environment and embryo receptivity post‐calving may be compromised in animals with High NEB. This underscores the potential role of NEB levels in influencing reproductive success during the post‐calving period.

### 3.5 UF‐EVs Modulate Epithelial Uterine Naïve Cells in NEB‐Dependent and Time‐Specific Manner

**Figure 8:**
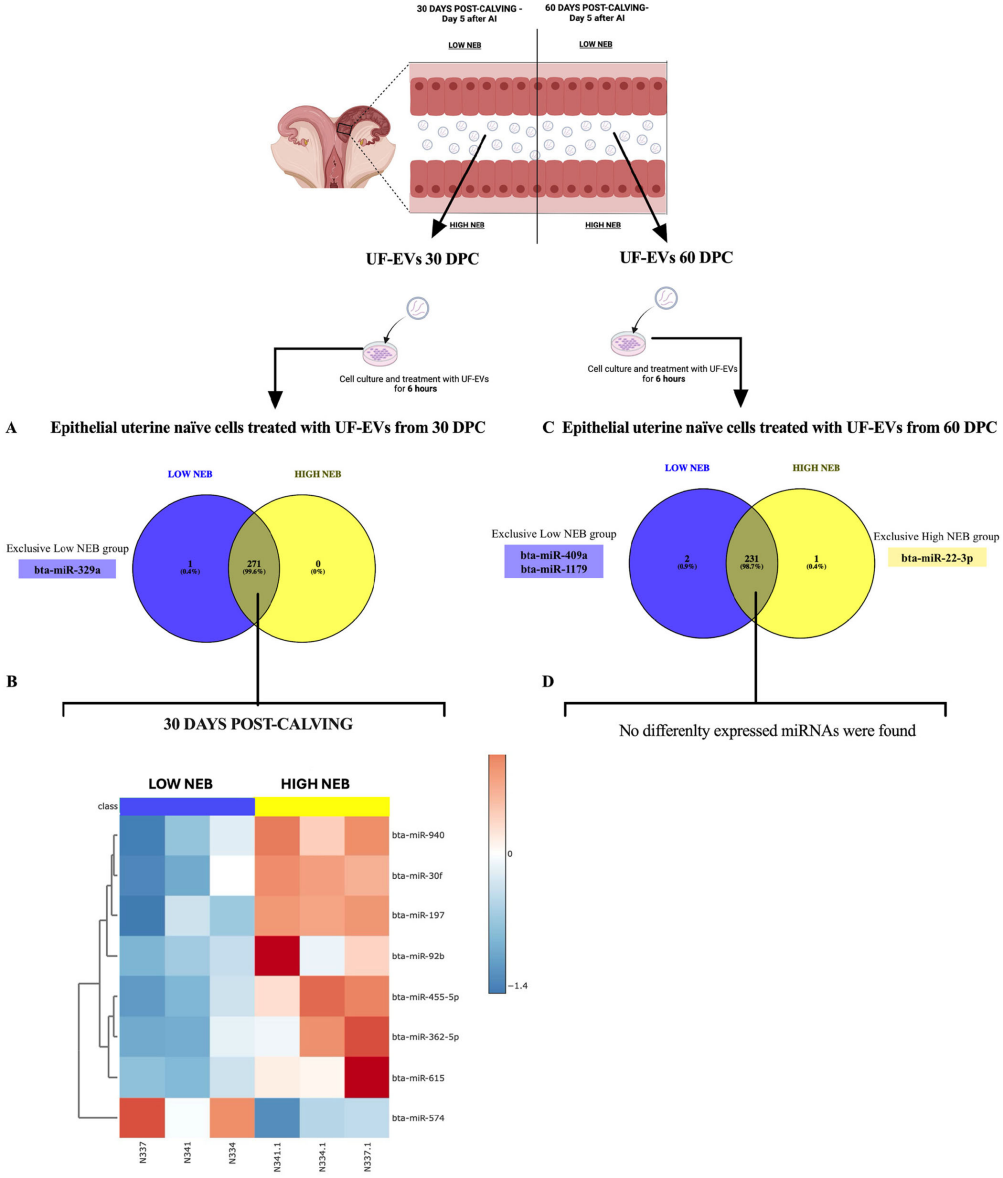
MiRNA profile of the epithelial uterine naïve cells treated with UF‐EVs at 30 and 60 DPC. To investigate if UF‐EVs can modulate the uterine cells, epithelial uterine naïve cells were treated for 6 h with UF‐EVs from dairy cows that presented Low or High NEB at 30 and 60 DPC. After the treatment, the epithelial uterine naïve cells were collected, and the miRNA profile was performed. (A) Venn diagram demonstrating the distribution of the 278 miRNAs present in epithelial uterine naïve cells (*n* = 3) treated with UF‐EVs at 30 DPC. (B) Heatmap demonstrating that seven miRNAs were upregulated in the cells (*n* = 3) treated with UF‐EVs from dairy cows that presented High NEB (red color), and one miRNA was upregulated in the cells (*n* = 3) treated with UF‐EVs from dairy cows that presented Low NEB (red color). (C) Venn diagram demonstrating the distribution of the 234 miRNAs present in epithelial uterine naïve cells (*n* = 3) treated with UF‐EVs at 60 DPC. (D) No differently expressed miRNA were found. Heat map data are based on significantly different miRNAs, *p* < 0.05. DPC, days post‐calving.

**Figure 9:**
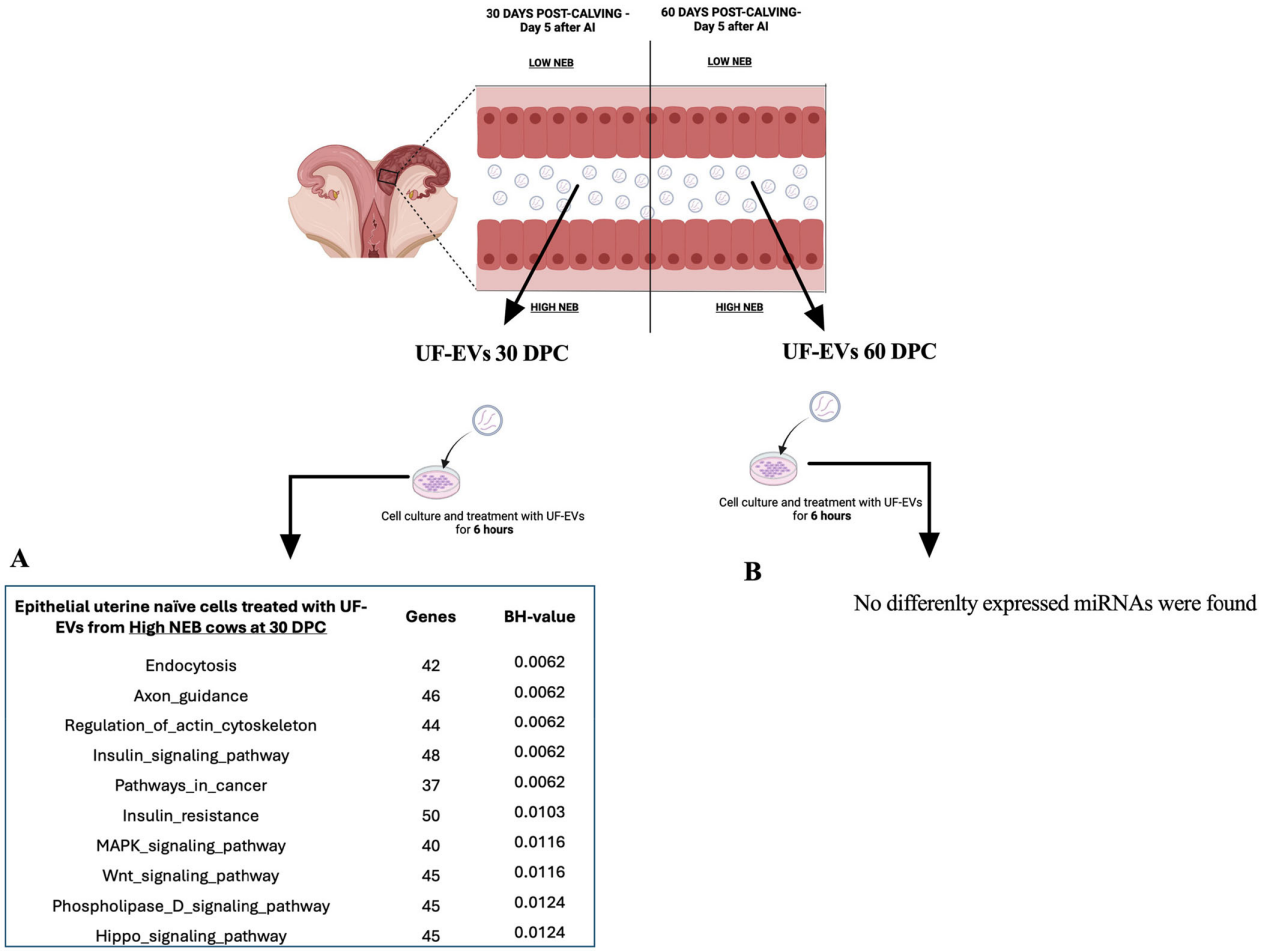
Enrichment analysis performed in miRWalk 3.0 software of predicted pathways modulated by upregulated miRNAs of epithelial uterine naïve cells treated with UF‐EVs from dairy cows that presented Low or High NEB at 30‐ and 60‐day post‐calving. (A) Top 10 predicted pathways regulated by 7 miRNAs upregulated in epithelial uterine naïve cells treated with UF‐EVs from dairy cows that presented High NEB at 30 DPC. (B) No pathways presented because no differently expressed miRNAs were found. DPC, days post‐calving; NEB, negative energy balance; sEVs, small extracellular vesicles.

Epithelial uterine naïve cells treated with UF‐EVs from 30 to 60 DPC dairy cows with Low and High NEB demonstrated distinct miRNA profiles. Validation through immunofluorescence and RT‐qPCR confirmed the presence of cytokeratin and *KRT18* markers (Figure S2). In the immunofluorescence images, cytokeratin‐positive staining was clearly detected; however, a small group of cells did not show detectable cytokeratin labeling. This may reflect reduced cytokeratin expression levels below the detection threshold in certain cells or the natural differentiation of epithelial cells during in vitro culture, a phenomenon previously reported by (Sponchiado et al. 2020). Total RNA was extracted from the treated cells to analyze 382 bovine miRNAs. At 30 DPC, cells treated with UF‐EVs from Low and High NEB cows expressed 293 and 288 miRNAs, respectively (Table S5). One miRNA (miR‐329a) was unique to Low NEB‐treated cells, and 271 miRNAs were commonly expressed between the groups (Figure 8A). Among these, eight miRNAs were differentially expressed: seven miRNAs (bta‐miR‐197, bta‐miR‐30f, bta‐miR‐362‐5p, bta‐miR‐455‐5p, bta‐miR‐615, bta‐miR‐92b, bta‐miR‐940) were upregulated in High NEB‐treated cells, and one miRNA (miR‐574) was upregulated in the Low NEB‐treated cells (*p* < 0.05; Figure 8B) treated with UF‐EVs from 30 DPC groups. Functional analysis of these miRNAs identified 30 biologically relevant pathways, with the 10 most significant pathways including Endocytosis, Axon guidance, Regulation of actin cytoskeleton, Insulin signaling pathway, Insulin signaling pathway, Pathways in cancer, Insulin resistance, MAPK signaling pathway, WNT signaling pathway, Phospholipase D signaling pathway, Hippo signaling pathway (*p* < 0.05; Figure 9A). These pathways are predominantly linked to cell metabolism.

At 60 DPC, cells treated with UF‐EVs from Low and High NEB cows expressed 269 and 270 miRNAs, respectively (Table S6). Two miRNAs (miR‐409a and miR‐1179) were uniquely detected in Low NEB‐treated cells, while one miRNA (miR‐22‐3p) was unique to High NEB‐treated cells (Figure 8C) treated with UF‐EVs from 60 DPC groups. A total of 231 miRNAs were commonly expressed between groups; however, none showed statistical differences (*p* > 0.05; Figures 8D and 9B). These findings indicate that UF‐EVs from Low and High NEB dairy cows can modulate epithelial uterine naïve cells, with modulation being more pronounced at 30 DPC compared to 60 DPC, reflecting NEB intensity and temporal changes in miRNA content. In summary, these results suggest that epithelial uterine naïve cells are modulated based on the content of UF‐EVs. At 30 DPC, the epithelial uterine cells from both groups are still recovering from the effects of NEB. However, by 60 DPC, UF‐EVs from animals experiencing Low NEB appear to facilitate recovery from the injury caused by NEB, while UF‐EVs from High NEB animals do not support the establishment of a uterine environment conducive to embryo receptivity.

## 4 Discussion

Due to the critical role of the uterine environment in embryo development, we investigated the effects of NEB intensity on this environment at 30‐ and 60‐DPC (5 days after AI), which corresponds to the period close to metabolic challenge (30 DPC) and the time when dairy cows are typically inseminated (60 DPC). The key findings from this in vivo study were as follows: (1) Blood NEFA concentration effectively distinguished cows with Low and High NEB; (2) the miRNA profile of UF‐EVs was modulated by metabolic status throughout the post‐calving period; (3) the transcriptomic profile of UEpC was influenced by the intensity of NEB and the post‐calving period, revealing that the uterine environment at 60 DPC in dairy cows with High NEB was less conducive to meeting the early requirements for embryo development; and (4) UF‐EVs were capable to emulate this phenotype in epithelial uterine naïve cells. The results suggest that, based on the miRNA signature and transcriptomic analysis, the uterine environment at 60 DPC from cows with High NEB post‐calving appears suboptimal for supporting embryo development, while the environment from dairy cows with Low NEB is more conducive to embryo development. Furthermore, the supplementation of naive epithelial cell cultures with UF‐EVs from both Low and High NEB cows induced molecular changes in the cells that mirrored the in vivo profiles of the endometrial samples. Collectively, these findings highlight the lasting effects of post‐calving metabolic status on the uterine environment and its potential implications for reproductive performance in dairy cows.

Blood NEFA and BHB concentrations are commonly used as indicators of NEB (Herdt 2000). Previous studies have shown that lactating cows with average NEFA concentrations ≥ 0.3 mmol/L (Chapinal et al. 2012; Ospina et al. 2010b) and BHB > 0.65 mmol/L (Girard et al. 2015) experience negative impacts on reproductive performance. In this study, we classified the cows into Low or High NEB groups based on their blood NEFA concentrations during the 3 weeks post‐calving to evaluate whether NEB intensity affects the uterine environment. Moreover, we explored whether increased NEFA concentrations during this early post‐calving period could have lasting effects on the uterine environment at 30 and 60 DPC—a time when cows are no longer in NEB—coinciding with the period when the uterus is preparing to receive an embryo (5 days after AI). At 3 weeks post‐calving, the cows showed clear separation between the groups, especially in NEFA concentrations.

To evaluate the changes in the uterine environment, we isolated UF‐EVs from the UF. Several techniques are available for isolating sEVs, including size‐exclusion chromatography and polymer‐based precipitation methods such as ExoQuick. In the present study, we chose to isolate UF‐EVs using ultracentrifugation. While this methodology has the potential to cause vesicle disruption and co‐isolate non‐vesicular components, ultracentrifugation remains a gold standard for EV isolation from complex biological fluids. In addition, the large volume obtained by the uterine flushing played an important role in our decision to avoid the concentration of unknown molecules. The ultracentrifugation has been extensively validated by our research group in previous studies involving reproductive tract samples (Andrade et al. 2017; De Ávila et al. 2020; Bastos et al. 2025; Bastos et al. 2023; Mazzarella et al. 2021; da Silveira et al. 2012, 2017). Our protocol has been optimized to reduce vesicle damage and consistently yield EV preparations suitable for downstream RNA‐based analyses, such as miRNAs and RNA‐sequencing. The validation of UF‐EVs isolation followed the Minimal Information for Studies of Extracellular Vesicles 2023 guidelines (Welsh et al. 2024). Our results revealed a population of sEVs with the expected size (< 200 nm) and cup‐shaped morphology. After confirming the morphological characteristics, we further validated the sEVs isolation protocol by evaluating the specific proteins present in sEVs such as as Alix, Syntenin, CD9 and CD63. Consistent with our findings, (Bridi et al. 2025; Fiorenza et al. 2024) also identified these markers in sEVs using nano‐flow analysis. Using a different technique, western blot, Burns et al. (2014) similarly detected CD63 in sEVs derived from ewe UF. Collectively, these results support the effectiveness of our protocol in isolating UF‐EVs. When we evaluated mode size and particle concentration, no differences were identified between the groups, suggesting that NEB intensity does not affect the uterine secretion of sEVs at 30 and 60 DPC. Although the concentration and size of UF‐EVs were similar between cows with Low and High NEB at 30 and 60 DPC, distinct miRNA profiles were identified between the groups.

Firstly, we aimed to evaluate the miRNA profile of UF‐EVs collected at 30 and 60 DPC from cows with Low or High NEB to characterize the uterine environment. At 30 DPC, we identified 19 miRNAs that were upregulated in dairy cows with High NEB. These miRNAs were involved in pathways such as MAPK, cell metabolism, and immune response. The MAPK pathway plays an important role in cell proliferation, differentiation, and apoptosis (W. Zhang and Liu 2002) and is particularly relevant during uterine involution. Uterine involution is a physiological process through which the uterus returns to its pre‐pregnancy dimensions, involving repair and regeneration of the endometrium in dairy cows. However, metabolic disorders can delay this process (Braga Paiano et al. 2019). Dairy cows affected by metabolic diseases also show alterations in innate immunity, which can contribute to the development of uterine diseases (Dervishi et al. 2016; G. Zhang et al. 2016). In addition, the connection between metabolic diseases and uterine involution may be mediated by immune responses. Furthermore, miR‐31 levels were found to be elevated in endometrial tissues from humans with recurrent implantation failure, suggesting a potential correlation with implantation failure (Azarpoor et al. 2020). In a mouse endometrial cell line, increased miR‐200c levels have been linked with impaired uterine receptivity (Zheng et al. 2017). Both miR‐31 and miR‐200c were elevated in cows with High NEB at 30 DPC, and the generalized upregulation of miRNAs in the High NEB group indicates a more reactive uterine state, potentially reflecting ongoing cellular repair processes and heightened immune activity required for the completion of uterine involution. These findings contrast with animals that experienced Low NEB, who presented a large number of transcripts upregulated in the uterine cells, leading to a uterine environment primed to respond to metabolism challenges and better equipped to achieve complete uterine recovery. Based on these results, we speculate that, at 30 DPC, the uterine environment in High NEB cows is still undergoing restoration following both parturition and the associated metabolic stress.

Based on our findings, cows experiencing Low NEB after calving are able to regulate tissue remodeling and reorganize the uterine environment earlier than cows experiencing High NEB after calving. At 60 DPC, analysis of the miRNA profile of UF‐EVs revealed one exclusive miRNA (miR‐369‐3p) in cows that experienced Low NEB. Upregulation of miR‐369‐3p is related to increased anti‐inflammatory cytokine production in dendritic cells (Scalavino et al. 2020). Previous studies have demonstrated that NEB predisposes dairy cows to uterine diseases (Bicalho et al. 2017; Nicola et al. 2022) and exposure to high NEFA concentrations stimulates the production of pro‐inflammatory cytokines in uterine cells (Chankeaw et al. 2018). In addition, among 28 miRNAs differentially expressed between the groups, 27 were elevated in UF‐EVs from dairy cows that experienced Low NEB. These upregulated miRNAs are involved in pathways related to cell remodeling. Altogether, these results suggest that dairy cows with Low NEB after calving have a uterine environment that is more capable of responding to inflammatory agents. One miRNA (miR‐27a‐5p) was increased in UF‐EVs from dairy cows with High NEB at 60 DPC. The downregulation of miR‐27a‐5p has been linked to alleviating inflammatory responses in rat pancreatic acinar cells (Zhu et al. 2020). Also, miR‐27a‐5p may regulate oxidative stress in rats (D. Li et al. 2020). Oxidative stress has also been observed in uterine cells after treatment with NEFA and BHB (Ferst et al. 2021; P. Li et al. 2019), which can negatively affect reproductive cells. These results suggest that the uterine environment of dairy cows with High NEB retains negative effects related to oxidative stress and inflammatory response at 60 DPC.

Since cell‐to‐cell communication in the uterus can be mediated by EVs, we evaluated the transcriptome profile of UEpC to investigate whether the information present in UF‐EVs is reflected in these cells. At 30 DPC, a time close to the metabolic challenge, UEpC from cows that experienced Low NEB showed increased expression of transcripts related to cell proliferation (*IGF2* and *IGFBP3*). UEpC were collected 5 days after AI, when the uterine environment is primed for embryo reception. At this stage, an endometrium that is less engaged in proliferation is more compatible and receptive to the early needs of the embryo. These results are in accordance with previous studies that evaluated transcript levels in uterine cells on day 7 after estrous (Sponchiado et al. 2017). The increase in the transcript *MUC4* was also detected in UEpC. Mucins are molecules that impact embryo implantation (Johnson et al. 2001), and elevated *MUC4* expression might contribute to infertility in cows (Wagener et al. 2017). In contrast, in UEpC from dairy cows that experienced High NEB post‐calving, we observed increased expression of transcripts related to inflammation (*NFAT5*), immune response (*IKZF2*), and negative effects on embryo development (*SLC10A1*) at 30 DPC. NFAT5 plays a crucial role in the inflammatory response to hypertonic stress (Lee et al. 2019), while IKZF2, expressed in regulatory T cells, helps to regulate their activity in an inflammatory context (Hetemäki et al. 2021). SLC10A1 is a solute carrier associated with inefficient trafficking of macromolecules in the placenta, which can negatively affect fetal development (Salilew‐Wondim et al. 2013). Previous studies have also demonstrated that NEB alters gene expression in uterine cells (Chankeaw et al. 2021; Wathes et al. 2009). Taken together, these results suggest that uterine cells from cows with High NEB at 30 DPC are not prepared to receive the embryo.

Dairy cows are usually inseminated around 60 DPC, at which point the uterus must be prepared to receive the embryo. To investigate whether metabolic status affects uterine cells and disrupts this environment, we also evaluated the transcriptome profile of UEpC at 60 DPC. The same cows collected at 30 DPC were resynchronized and collected again at 60 DPC. At this stage, the information in the UEpC appears to differ based on metabolic status. In the uterine cells from dairy cows that experienced Low NEB, we observed an increase in transcripts related to conceptus protection from inflammation and the maternal immune system, specifically *IDO1* (Groebner et al. 2011), indicating an environment more receptive to the embryo. In contrast, uterine cells from cows with High NEB showed increased expression of transcripts related to proliferation, such as *JUN*, at 60 DPC, suggesting that these cells are still engaged in proliferation, which is less compatible with the early requirements of the embryo. These findings highlight that the RNA profile of uterine cells is influenced by both the NEB intensity and the post‐calving period.

To further understand the impact of NEB on the uterine environment, we performed a functional study to evaluate whether UF‐EVs could modulate uterine cells that were not exposed to a metabolic challenge (epithelial uterine naïve cells). We collected epithelial uterine naïve cells from dairy heifers and treated them with UF‐EVs collected at 30 and 60 DPC from dairy cows with either Low or High NEB. After a 6‐hour culture, we assessed the miRNA profile of the epithelial uterine naïve cells. In this experiment, UF‐EV samples from animals classified as High or Low NEB were pooled as a strategy to reduce the influence of individual animal variability and to minimize potential confounding effects related to sample collection procedures. When comparing the miRNA profiles of the cells treated with UF‐EVs from dairy cows with Low or High NEB at 30 DPC, we identified one miRNA upregulated in cells treated with UF‐EVs from Low NEB cows, and seven miRNAs upregulated in cells treated with UF‐EVs from High NEB cows. These miRNAs modulate pathways such as MAPK signaling and insulin resistance, which were also regulated by UF‐EVs collected at 30 DPC. When epithelial uterine naïve cells were treated with UF‐EVs collected at 60 DPC, we identified two exclusives miRNAs (miR‐409a and miR‐1179) in cells treated with UF‐EVs from cows with Low NEB, and one exclusive miRNA (miR‐22‐3p) in cells treated with UF‐EVs from cows with High NEB. MiR‐22‐3p is known to inhibit cell activity and promote cell apoptosis (Lu et al. 2023). Moreover, this miRNA can modulate immune cell function and may be implicated in autoimmune diseases (Wang et al. 2017). Therefore, the presence of this miRNA could serve as a negative indicator for uterine cell function. These results demonstrate that UF‐EVs can modulate epithelial uterine naïve cells based on metabolic status and the post‐calving period.

To minimize potential confounding factors, cows presenting uterine disorders (e.g., clinical or subclinical endometritis), calving difficulties, or other clinical diseases were excluded from the study. As a result, the final sample consisted of seven healthy animals (Low NEB = 3; High NEB = 4), which we acknowledge as a limitation of this study. Although these inclusion criteria allowed for a more homogeneous study population and reduced variability unrelated to NEB status, the small sample size may have limited the statistical power to detect additional differences in EV cargo composition. Nevertheless, the fact that significant differences were observed in the EV and naive UEpCs miRNA profiles, as well as in the uterine cells RNA‐Seq data even with a limited number of animals suggests that the effects identified are robust and biologically meaningful.

In conclusion, we demonstrate the effects of post‐calving NEB on the uterine environment of dairy cows at 30 and 60 DPC through miRNA and transcriptomic analyses. Our mechanistic experiment further suggests that similar pathways were predicted to be modulated upon UF‐EVs treatment, suggesting that UF‐EVs play a role in modulating the endometrial response. To the best of our knowledge, this is the first study to show that cows that experienced NEB post‐calving still exhibit effects on the uterine environment at 60 DPC—a time when cows are no longer in NEB—depending on their metabolic status. At 30 DPC, cows with High NEB showed evidence of a uterus still engaged in repair and immune activity, whereas cows with Low NEB displayed a uterine environment better prepared for full recovery. By 60 DPC, the uterine environment in High NEB cows remained unfavorable for embryo development, while in Low NEB cows appeared more favorable for embryo receptivity, based on the our molecular analysis. Importantly, although our data suggests a possible comprehension of the molecular changes experienced by the uterus of Low and High NEB animals, we have a small number of animals and we have not transferred embryos to really test the uterine receptivity. However, in an attempt to understand that we demonstrate that sEVs derived from UF can reproduce these phenotypes in naïve UEpCs, further supporting the role of EV‐mediated communication in the modulation of the uterine environment in response to metabolic status. Altogether, these findings indicate that the impact of NEB persists for at least 60 DPC, with its intensity shaping the uterine environment in ways that may influence subsequent fertility in dairy cows.

## Author Contributions

**Juliana Germano Ferst:** conceptualization, investigation, writing – original draft. **Matheus Andrade Chaves:** writing – review and editing, methodology. **Amanda Nespolo Silva:** methodology. **Schaienni Fontoura Saldanha:** methodology, writing – review and editing. **Rogério Ferreira:** methodology, formal analysis, writing – review and editing. **Ricardo Perecin Nociti:** formal analysis, writing – review and editing. **Angélica Camargo dos Santos:** formal analysis. **Samuel Volpe Souza:** methodology. **Marcos Roberto Chiaratti:** formal analysis, writing – review and editing. **Guilherme Pugliesi:** methodology, writing – review and editing. **Felipe Perecin:** writing – review and editing, investigation, resources. **Flávio Vieira Meirelles:** resources. **Juliano Coelho da Silveira:** supervision, resources, conceptualization, writing – review and editing, funding acquisition.

## Conflicts of Interest

The authors declare no conflicts of interest.

## Supporting information

Supporting material final.

## Data Availability

The datasets generated and/or analyzed during the current study are available from the corresponding author upon reasonable request.
