# Supplementary material for: Uterine Extracellular Vesicles Can Emulate the Long‐Term Effects of Post‐Partum Negative Energy Balance in Dairy Cows
Source: Mol Reprod Dev. 2025 Oct 23;92(10):e70062. doi: 10.1002/mrd.70062 (PMC12548006; doi:10.1002/mrd.70062)
Supplement: Supplementary file 1 — Supporting material final. [file MRD-92-e70062-s001.docx]

**SUPPLEMENTARY MATERIAL**

**FIG. SUPP. 1: Metabolic parameters evaluated in the serum of dairy cows at 14 days post-calving (DPC).** n = 7 total; Low NEB = 3; High NEB = 4. AST: aspartate aminotransferase; GGT: gamma-glutamyl transferase; CK: creatine kinase. * indicates statistical difference (P<0.05).


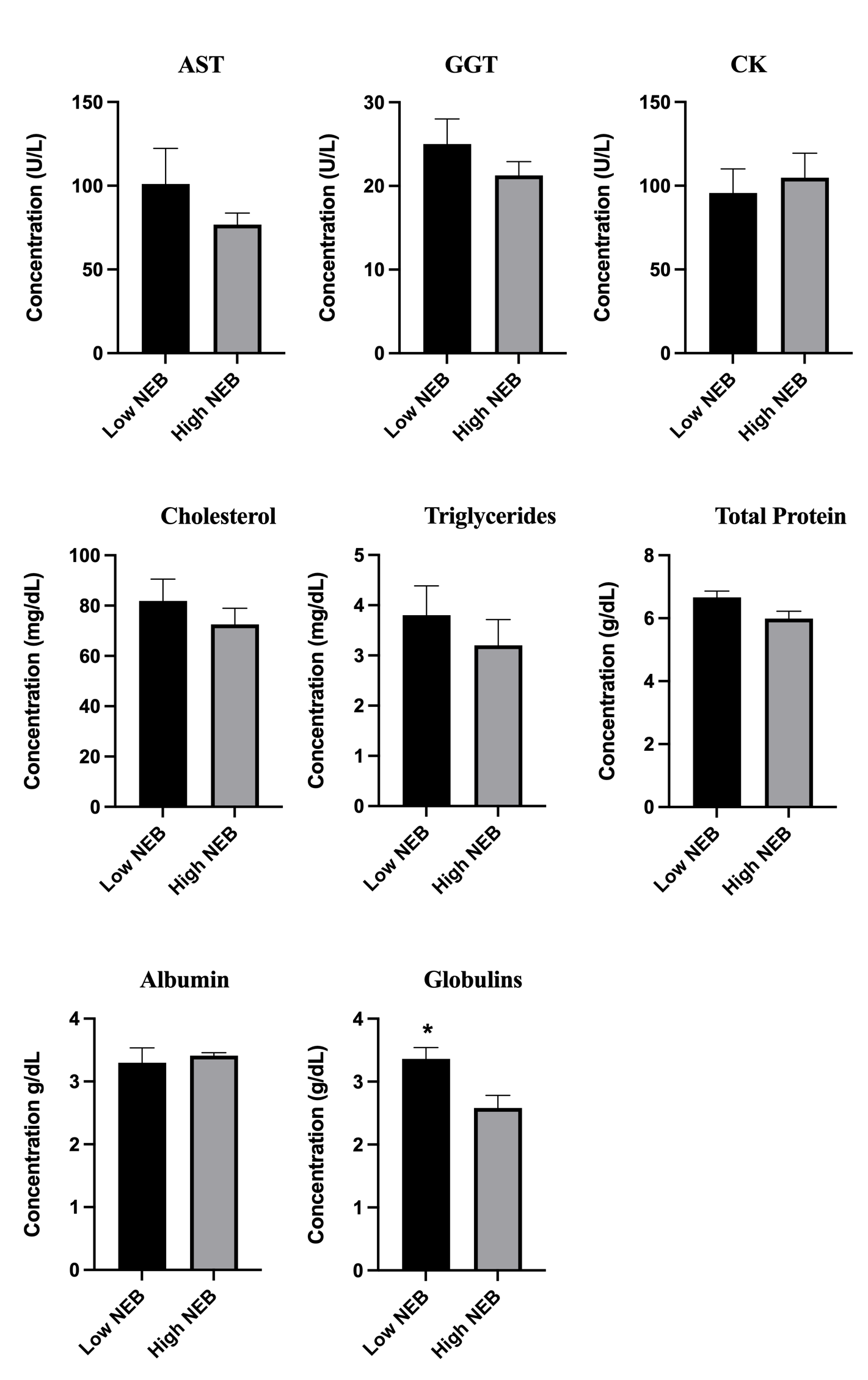


**FIG. SUPP. 2: Functional characteristics of cultured epithelial uterine naïve cells. A.** Light microscopy image of cultured epithelial uterine naïve cells (×20 magnification). **B.** Representative image of immunostaining for epithelial cell markers (cytokeratin, in green; ×10 magnification). **C.** Representative image of immunostaining for epithelial cell markers (cytokeratin, in green; ×40 magnification). **D.** Relative mRNA abundance of keratin 18 (*KRT18*) in epithelial uterine naïve cells treated with UF-EVs from dairy cows that presented Low or High NEB at 30 DPC. **E.** Relative mRNA abundance of keratin 18 (*KRT18*) in epithelial uterine naïve cells treated with UF-EVs from dairy cows that presented Low or High NEB at 60 DPC. DPC: days post-calving.

To investigate if uterine naïve cells can be modulated by UF-EVs according to the NEB intensity and the period post-calving, we collected epithelial uterine naïve cells from dairy heifers (n=3) and treated with pooled of UF-EVs from dairy cows with Low and High NEB at 30 and 60 DPC. First, for validation purposes we characterized the epithelial uterine naïve cells regarding the expression of cell-line specific markers. During primary culture monolayers consisted of a homogeneous population of epithelial-like adherent cells (Figure 10A). Immunostaining revealed that epithelial uterine naïve cells co-expressed cytokeratin, a typical cytoplasmic marker for epithelial origin (Figure 10B). We compared the abundance of transcripts for cell-origin by RT-qPCR in epithelial uterine naïve cells among treatments in the last passage. Gene expressed data showed the presence of transcripts for keratin 18 (*KRT18*) in the uterine naive cells treated with UF-EVs from dairy cows with Low and High NEB at 30 (Figure 10C) and 60 DPC (Figure 10D). These results are in accordance with ^63^.


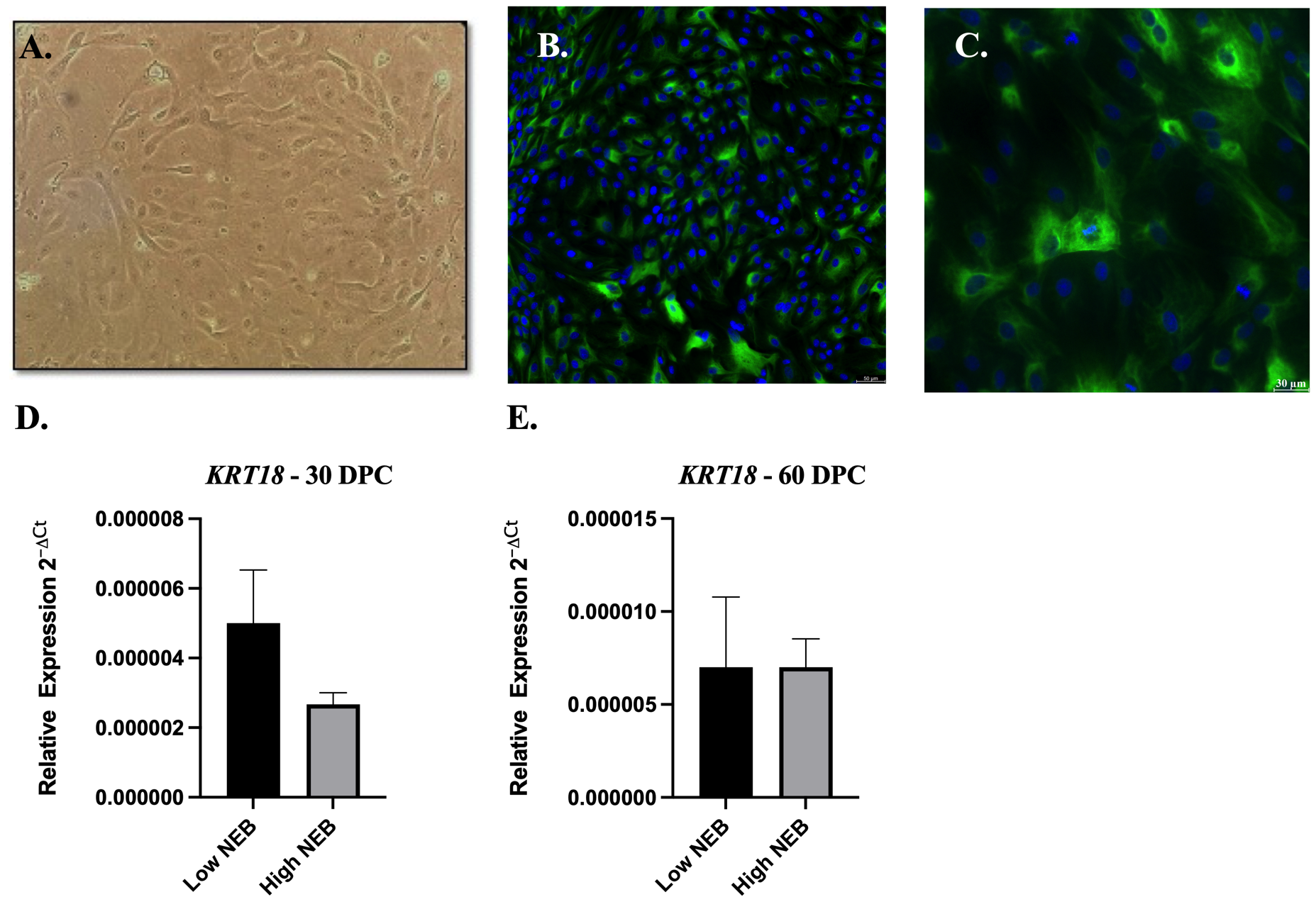


| **Supplementary Table S1:** List of miRNA forward primers used in the RT-qPCR reaction. | |
| --- | --- |
|  |  |
| **miRNA** | **Sequence (5'- 3')** |
| bta-let-7a-3p | CTATACAATCTACTGTCTTTC |
| bta-miR-103 | AGCAGCATTGTACAGGGCTATGA |
| bta-let-7a-5p | TGAGGTAGTAGGTTGTATAGTT |
| bta-miR-105a | TCAAATGCTCAGACTCCTGTGGT |
| bta-let-7b | TGAGGTAGTAGGTTGTGTGGTT |
| bta-miR-105b | TCAAATGCTCAGACTCCTTGGT |
| bta-let-7c | TGAGGTAGTAGGTTGTATGGTT |
| bta-miR-106a | AAAAGTGCTTACAGTGCAGGTA |
| bta-let-7d | AGAGGTAGTAGGTTGCATAGTT |
| bta-miR-106b | TAAAGTGCTGACAGTGCAGAT |
| bta-let-7e | TGAGGTAGGAGGTTGTATAGT |
| bta-miR-107 | AGCAGCATTGTACAGGGCTATC |
| bta-let-7f | TGAGGTAGTAGATTGTATAGTT |
| bta-miR-10a | TACCCTGTAGATCCGAATTTGTG |
| bta-let-7g | TGAGGTAGTAGTTTGTACAGTT |
| bta-miR-10b | TACCCTGTAGAACCGAATTTGTG |
| bta-let-7i | TGAGGTAGTAGTTTGTGCTGTT |
| bta-miR-122 | TGGAGTGTGACAATGGTGTTTG |
| bta-miR-1 | TGGAATGTAAAGAAGTATGTAT |
| bta-miR-124a | TAAGGCACGCGGTGAATGCCAAG |
| bta-miR-100 | AACCCGTAGATCCGAACTTGTG |
| bta-miR-124b | TAAGGCACGCGGTGAATGCCAAG |
| bta-miR-101 | TACAGTACTGTGATAACTGAA |
| bta-miR-125a | TCCCTGAGACCCTTTAACCTGTG |
| bta-miR-125b | TCCCTGAGACCCTAACTTGTGA |
| bta-miR-133b | TTTGGTCCCCTTCAACCAGCTA |
| bta-miR-126-3p | CGTACCGTGAGTAATAATGCG |
| bta-miR-133c | ATTTGGTTCCATTTTACCAGC |
| bta-miR-126-5p | CATTATTACTTTTGGTACGCG |
| bta-miR-134 | TGTGACTGGTTGACCAGAGTGG |
| bta-miR-127 | TCGGATCCGTCTGAGCTTGGCT |
| bta-miR-135a | TATGGCTTTTTATTCCTATGTGA |
| bta-miR-128 | TCACAGTGAACCGGTCTCTTT |
| bta-miR-135b | TATGGCTTTTCATTCCTATGTGA |
| bta-miR-129 | CTTTTTGCGGTCTGGGCTTGCT |
| bta-miR-136 | ACTCCATTTGTTTTGATGATGGA |
| bta-miR-129-3p | AAGCCCTTACCCCAAAAAGCAT |
| bta-miR-137 | TTATTGCTTAAGAATACGCGTAG |
| bta-miR-129-5p | CTTTTTGCGGTCTGGGCTTGCT |
| bta-miR-138 | AGCTGGTGTTGTGAATCAGGCCG |
| bta-miR-130a | CAGTGCAATGTTAAAAGGGCAT |
| bta-miR-139 | TCTACAGTGCACGTGTCTCCAGT |
| bta-miR-130b | CAGTGCAATGATGAAAGGGCAT |
| bta-miR-140 | TACCACAGGGTAGAACCACGGA |
| bta-miR-132 | TAACAGTCTACAGCCATGGTCG |
| bta-miR-141 | TAACACTGTCTGGTAAAGATGG |
| bta-miR-133a | TTTGGTCCCCTTCAACCAGCTG |
| bta-miR-142-3p | AGTGTTTCCTACTTTATGGATG |
| bta-miR-142-5p | CATAAAGTAGAAAGCACTAC |
| bta-miR-151-3p | CTAGACTGAAGCTCCTTGAGG |
| bta-miR-143 | TGAGATGAAGCACTGTAGCTCG |
| bta-miR-151-5p | TCGAGGAGCTCACAGTCTAGT |
| bta-miR-144 | TACAGTATAGATGATGTACTAG |
| bta-miR-152 | TCAGTGCATGACAGAACTTGGG |
| bta-miR-145 | GTCCAGTTTTCCCAGGAATCCCT |
| bta-miR-153 | TTGCATAGTCACAAAAGTGATC |
| bta-miR-146a | TGAGAACTGAATTCCATAGGTTGT |
| bta-miR-154a | TAGGTTATCCGTGTAGCCTTCG |
| bta-miR-146b | TGAGAACTGAATTCCATAGGCTGT |
| bta-miR-154b | AGAGGTCTTCCATGGTGCATTCG |
| bta-miR-147 | GTGTGCGGAAATGCTTCTGCTA |
| bta-miR-154c | AGATATTGCACGGTTGATCTCT |
| bta-miR-148a | TCAGTGCACTACAGAACTTTGT |
| bta-miR-155 | TTAATGCTAATCGTGATAGGGGT |
| bta-miR-148b | TCAGTGCATCACAGAACTTTGT |
| bta-miR-15a | TAGCAGCACATAATGGTTTGT |
| bta-miR-149-3p | GAGGGAGGGACGGGGGCTGTGC |
| bta-miR-15b | TAGCAGCACATCATGGTTTACA |
| bta-miR-149-5p | TCTGGCTCCGTGTCTTCACTCCC |
| bta-miR-16a | TAGCAGCACGTAAATATTGGTG |
| bta-miR-150 | TCTCCCAACCCTTGTACCAGTGT |
| bta-miR-16b | TAGCAGCACGTAAATATTGGC |
| bta-miR-17-3p | ACTGCAGTGAAGGCACTTGT |
| bta-miR-188 | CATCCCTTGCATGGTGGAGGGT |
| bta-miR-17-5p | CAAAGTGCTTACAGTGCAGGTAGT |
| bta-miR-18a | TAAGGTGCATCTAGTGCAGATA |
| bta-miR-181a | AACATTCAACGCTGTCGGTGAGTT |
| bta-miR-18b | TAAGGTGCATCTAGTGCAGTTA |
| bta-miR-181b | AACATTCATTGCTGTCGGTGGGTT |
| bta-miR-190a | TGATATGTTTGATATATTAGGT |
| bta-miR-181c | AACATTCAACCTGTCGGTGAGTTT |
| bta-miR-190b | TGATATGTTTGATATTGGGTT |
| bta-miR-181d | AACATTCATTGTTGTCGGTGGGT |
| bta-miR-191 | CAACGGAATCCCAAAAGCAGCTG |
| bta-miR-182 | TTTGGCAATGGTAGAACTCACACT |
| bta-miR-192 | CTGACCTATGAATTGACAGCCAG |
| bta-miR-183 | TATGGCACTGGTAGAATTCACTG |
| bta-miR-193a | GGGACTTTGTAGGCCAGTT |
| bta-miR-184 | TGGACGGAGAACTGATAAGGGT |
| bta-miR-193a-3p | AACTGGCCTACAAAGTCCCAGT |
| bta-miR-185 | TGGAGAGAAAGGCAGTTCCTGA |
| bta-miR-193a-5p | TGGGTCTTTGCGGGCGAGATGA |
| bta-miR-186 | CAAAGAATTCTCCTTTTGGGCT |
| bta-miR-193b | AACTGGCCCACAAAGTCCCGCTTT |
| bta-miR-187 | TCGTGTCTTGTGTTGCAGCCGG |
| bta-miR-194 | TGTAACAGCAACTCCATGTGGA |
| bta-miR-195 | TAGCAGCACAGAAATATTGGCA |
| bta-miR-200c | TAATACTGCCGGGTAATGATGGA |
| bta-miR-196a | TAGGTAGTTTCATGTTGTTGGG |
| bta-miR-202 | TTCCTATGCATATACTTCTTT |
| bta-miR-196b | TAGGTAGTTTCCTGTTGTTGGGA |
| bta-miR-204 | TTCCCTTTGTCATCCTATGCCT |
| bta-miR-197 | TTCACCACCTTCTCCACCCAGC |
| bta-miR-205 | TCCTTCATTCCACCGGAGTCTG |
| bta-miR-199a-3p | ACAGTAGTCTGCACATTGGTTA |
| bta-miR-206 | TGGAATGTAAGGAAGTGTGTGG |
| bta-miR-199a-5p | CCCAGTGTTCAGACTACCTGTT |
| bta-miR-208a | ATAAGACGAGCAAAAAGCTTGT |
| bta-miR-199b | CCCAGTGTTTAGACTATCTGTTC |
| bta-miR-208b | ATAAGACGAACAAAAGGTTTGT |
| bta-miR-199c | TACAGTAGTCTGCACATTGG |
| bta-miR-20a | TAAAGTGCTTATAGTGCAGGTAG |
| bta-miR-19a | TGTGCAAATCTATGCAAAACTGA |
| bta-miR-20b | CAAAGTGCTCACAGTGCAGGTA |
| bta-miR-19b | TGTGCAAATCCATGCAAAACTGA |
| bta-miR-21-3p | AACAGCAGTCGATGGGCTGTCT |
| bta-miR-200a | TAACACTGTCTGGTAACGATGTT |
| bta-miR-21-5p | TAGCTTATCAGACTGATGTTGACT |
| bta-miR-200b | TAATACTGCCTGGTAATGATG |
| bta-miR-210 | ACTGTGCGTGTGACAGCGGCTGA |
| bta-miR-211 | TTCCCTTTGTCATCCTTTGCC |
| bta-miR-22-5p | AGTTCTTCAGTGGCAAGCTTTA |
| bta-miR-212 | ACCTTGGCTCTAGACTGCTTACT |
| bta-miR-221 | AGCTACATTGTCTGCTGGGTTT |
| bta-miR-214 | ACAGCAGGCACAGACAGGCAGT |
| bta-miR-222 | AGCTACATCTGGCTACTGGGT |
| bta-miR-215 | ATGACCTATGAATTGACAGACA |
| bta-miR-223 | TGTCAGTTTGTCAAATACCCCA |
| bta-miR-216a | TAATCTCAGCTGGCAACTGTGA |
| bta-miR-224 | CAAGTCACTAGTGGTTCCGTTTA |
| bta-miR-216b | AAATCTCTGCAGGCAAATGTGA |
| bta-miR-23a | ATCACATTGCCAGGGATTTCCA |
| bta-miR-217 | TACTGCATCAGGAACTGATTGGAT |
| bta-miR-23b-3p | ATCACATTGCCAGGGATTACCAC |
| bta-miR-218 | TTGTGCTTGATCTAACCATGTG |
| bta-miR-23b-5p | GGGTTCCTGGCATGCTGATTT |
| bta-miR-219 | AGAGTTGAGTCTGGACGTCCCG |
| bta-miR-24 | GTGCCTACTGAGCTGATATCAGT |
| bta-miR-219-3p | AGAATTGTGGCTGGACATCTG |
| bta-miR-24-3p | TGGCTCAGTTCAGCAGGAACAG |
| bta-miR-219-5p | TGATTGTCCAAACGCAATTCTT |
| bta-miR-25 | CATTGCACTTGTCTCGGTCTGA |
| bta-miR-22-3p | AAGCTGCCAGTTGAAGAACTG |
| bta-miR-26a | TTCAAGTAATCCAGGATAGGCT |
| bta-miR-26b | TTCAAGTAATTCAGGATAGGTT |
| bta-miR-29d-3p | TAGCACCATTTGAAATCGATTA |
| bta-miR-26c | AGCCTATCCTGGATTACTTGAA |
| bta-miR-29d-5p | TGACCGATTTCTCCTGGTGTT |
| bta-miR-27a-3p | TTCACAGTGGCTAAGTTCCG |
| bta-miR-29e | TAGCATCATTTGAAATCAGTGTTT |
| bta-miR-27a-5p | AGGGCTTAGCTGCTTGTGAGCA |
| bta-miR-301a | CAGTGCAATAGTATTGTCAAAGCAT |
| bta-miR-27b | TTCACAGTGGCTAAGTTCTGC |
| bta-miR-301b | CAGTGCAATGATATTGTCAAAGCAT |
| bta-miR-28 | AAGGAGCTCACAGTCTATTGAG |
| bta-miR-302a | AAGTGCTTCCATGTTTTAGTGA |
| bta-miR-296-3p | GAGGGTTGGGCGGAGGCTTTCC |
| bta-miR-302b | TAAGTGCTTCCATGTTTTAGTAG |
| bta-miR-296-5p | GAGGGCCCCCCCCAATCCT |
| bta-miR-302c | TAAGTGCTTCCATGTTTCAGTGG |
| bta-miR-299 | TGGTTTACCGTCCCACATACAT |
| bta-miR-302d | TAAGTGCTTCCATGTTTTAGT |
| bta-miR-29a | CTAGCACCATCTGAAATCGGTTA |
| bta-miR-3064 | TTGCCACACTGCAACACCTTACA |
| bta-miR-29b | TAGCACCATTTGAAATCAGTGTT |
| bta-miR-30a-5p | TGTAAACATCCTCGACTGGAAGCT |
| bta-miR-29c | TAGCACCATTTGAAATCGGTTA |
| bta-miR-30b-3p | CTGGGAGGTGGATGTTTACTT |
| bta-miR-30b-5p | TGTAAACATCCTACACTCAGCT |
| bta-miR-328 | CTGGCCCTCTCTGCCCTTCCGT |
| bta-miR-30c | TGTAAACATCCTACACTCTCAGC |
| bta-miR-329a | AACACACCTGGTTAACCTTTTT |
| bta-miR-30d | TGTAAACATCCCCGACTGGAAGCT |
| bta-miR-329b | AGAGGTTTTCTGGGTTTCTGTTT |
| bta-miR-30e-5p | TGTAAACATCCTTGACTGGAAGCT |
| bta-miR-330 | GCAAAGCACACGGCCTGCAGAGA |
| bta-miR-30f | TGTAAACACCCTACACTCTCAGCT |
| bta-miR-331-3p | GCCCCTGGGCCTATCCTAGAA |
| bta-miR-31 | AGGCAAGATGCTGGCATAGCT |
| bta-miR-331-5p | TCTAGGTATGGTCCCAGG |
| bta-miR-32 | TATTGCACATGACTAAGTTGCAT |
| bta-miR-335 | TCAAGAGCAATAACGAAAAATGT |
| bta-miR-320a | AAAAGCTGGGTTGAGAGGGCGA |
| bta-miR-338 | TCCAGCATCAGTGATTTTGTTGA |
| bta-miR-320b | AGCTGGGTTGAGAGGGTGGT |
| bta-miR-339a | TCCCTGTCCTCCAGGAGCTCAC |
| bta-miR-323 | GCACATTACACGGTCGACCTCT |
| bta-miR-339b | TCCCTGTCCTCCAGGAGCTC |
| bta-miR-324 | CGCATCCCCTAGGGCATTGGTGT |
| bta-miR-33a | GTGCATTGTAGTTGCATTGCA |
| bta-miR-326 | CCTCTGGGCCCTTCCTCCAG |
| bta-miR-33b | GTGCATTGCTGTTGCATTGC |
| bta-miR-340 | TCCGTCTCAGTTACTTTATAGCC |
| bta-miR-365-3p | TAATGCCCCTAAAAATCCTTAT |
| bta-miR-342 | TCTCACACAGAAATCGCACCCATCT |
| bta-miR-365-5p | AGGGACTTTTGGGGGCAGATGTG |
| bta-miR-345-3p | CCTGAACTAGGGGTCTGGAG |
| bta-miR-367 | GAATTGCACTTTAGCAATGGTGA |
| bta-miR-345-5p | GCTGACTCCTAGTCCAGTGCT |
| bta-miR-369-3p | AATAATACATGGTTGATCTTT |
| bta-miR-346 | TGTCTGCCCGCATGCCTGCCTCT |
| bta-miR-369-5p | ATCGACCGTGTTATATTCGC |
| bta-miR-34a | TGGCAGTGTCTTAGCTGGTTGT |
| bta-miR-370 | GCCTGCTGGGGTGGAACCTGGT |
| bta-miR-34b | AGGCAGTGTAATTAGCTGATTG |
| bta-miR-371 | AAGTGCCGCCATGTTTTGAGTGT |
| bta-miR-34c | AGGCAGTGTAGTTAGCTGATTG |
| bta-miR-374a | TTATAATACAACCTGATAAGTG |
| bta-miR-361 | TTATCAGAATCTCCAGGGGTAC |
| bta-miR-374b | ATATAATACAACCTGCTAAGTG |
| bta-miR-362-3p | AACACACCTATTCAAGGATTC |
| bta-miR-375 | TTTTGTTCGTTCGGCTCGCGTGA |
| bta-miR-362-5p | AATCCTTGGAACCTAGGTGTGAGT |
| bta-miR-376a | ATCATAGAGGAAAATCCACGT |
| bta-miR-363 | ATTGCACGGTATCCATCTGCG |
| bta-miR-376b | ATCATAGAGGAAAATCCATGTT |
| bta-miR-376c | GTGGATATTCCTTCTATGTTTA |
| bta-miR-382 | GAAGTTGTTCGTGGTGGATTCG |
| bta-miR-376d | ATCATAGAGGAAAATCCACAT |
| bta-miR-383 | AGATCAGAAGGTGATTGTGGCT |
| bta-miR-376e | AACATAGAGGAAAATCCACATT |
| bta-miR-409a | AGGTTACCCGAGCAACTTTGCAT |
| bta-miR-377 | ATCACACAAAGGCAACTTTTGT |
| bta-miR-409b | GGGGTTCACCGAGCAACATTC |
| bta-miR-378 | ACTGGACTTGGAGTCAGAAGGC |
| bta-miR-410 | AATATAACACAGATGGCCTGT |
| bta-miR-378b | ACTTGACTTGGAGTCAGAAGGC |
| bta-miR-411a | ATAGTAGACCGTATAGCGTACG |
| bta-miR-378c | ACTGGACTTGGAGTCAGAAGT |
| bta-miR-411b | TGGTCGACCATAAAACGTACGT |
| bta-miR-378d | CTGGACTTGGAGTCAGAAGACC |
| bta-miR-411c-3p | TGTATGTCAACTGATCCACAGT |
| bta-miR-379 | TGGTAGACTATGGAACGTAGG |
| bta-miR-411c-5p | GGTTGATCAGAGAACATACATT |
| bta-miR-380-3p | TATGTAATGTGGTCCACGTCT |
| bta-miR-412 | ACTTCACCTGGTCCACTAGCTGT |
| bta-miR-380-5p | TGGTTGACCATAGAACATGCGC |
| bta-miR-421 | ATCAACAGACATTAATTGGGCGC |
| bta-miR-381 | TATACAAGGGCAAGCTCTCTGT |
| bta-miR-423-3p | AAGCTCGGTCTGAGGCCCCTCAGT |
| bta-miR-423-5p | TGAGGGGCAGAGAGCGAGACTTT |
| bta-miR-449c | AGGCAGTGCATCTCTAGCTGG |
| bta-miR-424-3p | CAAAACGTGAGGCGCTGCTAT |
| bta-miR-449d | GAAGGCTGTGTGCTGTGGAG |
| bta-miR-424-5p | CAGCAGCAATTCATGTTTTGA |
| bta-miR-450a | TTTTGCGATGTGTTCCTAATAT |
| bta-miR-425-3p | ATCGGGAATGTCGTGTCCGCCC |
| bta-miR-450b | TTTTGCAATATGTTCCTGAATA |
| bta-miR-425-5p | ATGACACGATCACTCCCGTTGA |
| bta-miR-451 | AAACCGTTACCATTACTGAGTTT |
| bta-miR-429 | TAATACTGTCTGGTAATGCCGT |
| bta-miR-452 | TGTTTGCAGAGGAAACTGAGAC |
| bta-miR-431 | TGTCTTGCAGGCCGTCATGCAGG |
| bta-miR-4523 | GACCGAGAGGGCCTCGGCTGT |
| bta-miR-432 | TCTTGGAGTAGGTCATTGGGTGG |
| bta-miR-453 | AGGTTGTCCGTGGTGAGTTCGCA |
| bta-miR-433 | ATCATGATGGGCTCCTCGGTGT |
| bta-miR-454 | TAGTGCAATATTGCTTATAGGGT |
| bta-miR-448 | TTGCATATGTAGGATGTCCCAT |
| bta-miR-455-3p | GCAGTCCATGGGCATATACACT |
| bta-miR-449a | TGGCAGTGTATTGTTAGCTGGT |
| bta-miR-455-5p | TATGTGCCTTTGGACTACATC |
| bta-miR-449b | AGGCAGTGTATTGTTAGCTGGC |
| bta-miR-483 | TCACTCCTCTCCTCCCGTCTT |
| bta-miR-484 | TCAGGCTCAGTCCCCTCCCGAT |
| bta-miR-496 | TGAGTATTACATGGCCAATCTC |
| bta-miR-485 | AGAGGCTGGCCGTGATGAATTCG |
| bta-miR-497 | CAGCAGCACACTGTGGTTTGTA |
| bta-miR-486 | TCCTGTACTGAGCTGCCCCGAG |
| bta-miR-499 | TTAAGACTTGCAGTGATGTTT |
| bta-miR-487a | AATCATACAGGGACATCCAGT |
| bta-miR-500 | TAATCCTTGCTACCTGGGTGAGA |
| bta-miR-487b | AATCGTACAGGGTCATCCACTT |
| bta-miR-502a | AATGCACCTGGGCAAGGATTCA |
| bta-miR-488 | TTGAAAGGCTGTTTCTTGGTC |
| bta-miR-502b | AATCCACCTGGGCAAGGATTC |
| bta-miR-489 | GTGACATCACATATATGGCGAC |
| bta-miR-503-3p | GGAGTATTGTTTCTGCTGCCCGG |
| bta-miR-490 | CAACCTGGAGGACTCCATGCTG |
| bta-miR-503-5p | TAGCAGCGGGAACAGTACTG |
| bta-miR-491 | AGTGGGGAACCCTTCCATGAGG |
| bta-miR-504 | AGACCCTGGTCTGCACTCTGTC |
| bta-miR-493 | TGAAGGTCTACTGTGTGCCAGG |
| bta-miR-505 | CGTCAACACTTGCTGGTTTCCT |
| bta-miR-494 | TGAAACATACACGGGAAACCTC |
| bta-miR-532 | CATGCCTTGAGTGTAGGACCGT |
| bta-miR-495 | AAACAAACATGGTGCACTTCTT |
| bta-miR-539 | GGAGAAATTATCCTTGGTGTGT |
| bta-miR-541 | TGGTGGGCACAGAATCCGGCCT |
| bta-miR-582 | TTACAGTTGTTCAACCAGTTACT |
| bta-miR-542-5p | TCGGGGATCATCATGTCACGAG |
| bta-miR-584 | TGGTTTGCCTGGGACTGAG |
| bta-miR-543 | AAACATTCGCGGTGCACTTCTT |
| bta-miR-592 | ATTGTGTCAATATGCGATGATGT |
| bta-miR-544a | ATTCTGCATTTTTAGCAAGTTC |
| bta-miR-599 | GTTGTGTCAGTTTATCAAAC |
| bta-miR-544b | ATTCTGCATTTCTAACAAGTTC |
| bta-miR-615 | GGGGGTCCCCGGTGCTCGGATC |
| bta-miR-545-3p | ATCAACAAACATTTATTGTGTG |
| bta-miR-628 | ATGCTGACATATTTACTAGAGG |
| bta-miR-545-5p | TCAGTAAATGTTTATTGGATG |
| bta-miR-631 | AGACCTGGCTTAGACCTCAGC |
| bta-miR-551a | GCGACCCAATCTTGGTTTCCA |
| bta-miR-652 | AATGGCGCCACTAGGGTTGTG |
| bta-miR-551b | GGCGACCCATACTTGGTTTCAG |
| bta-miR-653 | GTGTTGAAACAATCTCTGTTG |
| bta-miR-562 | AAAGCAGCTGTACCATTTAC |
| bta-miR-654 | TATGTCTGCTGACCATCACCTT |
| bta-miR-568 | ATGTATAAATGTATACACAC |
| bta-miR-655 | ATAATACATGGTTAACCTCTCT |
| bta-miR-574 | TGAGTGTGTGTGTGTGAGTGTGTG |
| bta-miR-656 | AATATTATACAGTCAACCTCT |
| bta-miR-658 | GGCGGAGGGAAGCGGGTCCGTTGGT |
| bta-miR-758 | TTTGTGACCTGGTCCACTAACC |
| bta-miR-660 | TACCCATTGCATATCGGAGCTG |
| bta-miR-759 | GCAGACTGCAAACAATTTTGAC |
| bta-miR-664a | CAGGCTGGGGTGTGTGTGGATG |
| bta-miR-760-3p | CGGCTCTGGGTCTGTGGGGA |
| bta-miR-664b | TATTCATTTATCTCCCAGCCTAC |
| bta-miR-760-5p | CCCCTCAGTCCACCAGAGCCCG |
| bta-miR-665 | ACCAGTAGGCCGAGGCCCCT |
| bta-miR-761 | GCAGCAGGGTGAAACTGACACA |
| bta-miR-669 | TGTGGGTGTGTGCATGTGCGTG |
| bta-miR-763 | CCAGCTGGGAGGAACCAGTGGC |
| bta-miR-670 | TCCCTGAGTATATGTGGTGAA |
| bta-miR-764 | GGTGCTCACTCGTCCTTCT |
| bta-miR-671 | AGGAAGCCCTGGAGGGGCTGGAG |
| bta-miR-767 | TGCACCATGGTTGTCTGAGCATG |
| bta-miR-677 | CTCACTGATGAGCAGCTTCTGAC |
| bta-miR-769 | TGAGACCTCCGGGTTCTGAGCT |
| bta-miR-7 | TGGAAGACTAGTGATTTTGTTGTT |
| bta-miR-873 | GCAGGAACTTGTGAGTCTCCT |
| bta-miR-708 | AAGGAGCTTACAATCTAGCTGGG |
| bta-miR-874 | CTGCCCTGGCCCGAGGGACCGA |
| bta-miR-744 | TGCGGGGCTAGGGCTAACAGCA |
| bta-miR-875 | TATACCTCAGTTTTATCAGGTG |
| bta-miR-876 | TGGATTTCTTTGTGAATCACCA |
| bta-miR-98 | TGAGGTAGTAAGTTGTATTGTT |
| bta-miR-877 | GTAGAGGAGATGGCGCAGGG |
| bta-miR-99a-3p | CAAGCTCGCTTCTATGGGT |
| bta-miR-885 | TCCATTACACTACCCTGCCTCT |
| bta-miR-99a-5p | AACCCGTAGATCCGATCTTGT |
| bta-miR-9-3p | ATAAAGCTAGATAACCG |
| bta-miR-99b | CACCCGTAGAACCGACCTTGCG |
| bta-miR-9-5p | TCTTTGGTTATCTAGCTGTATG |
| bta-miR-1179 | AAGCATTCTTTCATTGGTTGG |
| bta-miR-92a | TATTGCACTTGTCCCGGCCTGT |
| bta-miR-1185 | AGAGGATACCCTTTGTATGTT |
| bta-miR-92b | TATTGCACTCGTCCCGGCCTCC |
| bta-miR-1193 | TAGGTCACCCGTTTGACTATC |
| bta-miR-93 | CAAAGTGCTGTTCGTGCAGGTA |
| bta-miR-1197 | TAGGACACATGGTCTACTTCT |
| bta-miR-935 | CCAGTTACCGCTTCCGCTACCGC |
| bta-miR-122 | TGGAGTGTGACAATGGTGTTTG |
| bta-miR-940 | AAGGCTGGGCCCCCGCTCCGC |
| bta-miR-1224 | GTGAGGACTCGGGAGGTGGAG |
| bta-miR-95 | TTCAACGGGTATTTATTGAGCA |
| bta-miR-1225-3p | CCGAGCCCCTGTGCCGCCCCCAG |
| bta-miR-96 | TTTGGCACTAGCACATTTTTGCT |
| bta-miR-1246 | AATGGATTTTTGGAGCAGG |
| bta-miR-1247-3p | CGGGAACGTCGGGACTGGAGC |
| bta-miR-1296 | TTAGGGCCCTGGCTCCATCTCC |
| bta-miR-1247-5p | ACCCGTCCCGTGCGTCCCCGGA |
| bta-miR-1298 | TTCATTCGGCTGTCCAGATGTA |
| bta-miR-1248 | ACCTTCTTGTATAAGCACTGTGCTAAA |
| bta-miR-1301 | TTGCAGCTGCCTAGGAGTGATTTC |
| bta-miR-1249 | ACGCCCTTCCCCCCCTTCTTCA |
| bta-miR-1306 | CCACCTCCCCTGCAAACGTCC |
| bta-miR-1260b | ATCCCACCACTGCCACCA |
| bta-miR-1307 | ACTCGGCGTGGCGTCGGTCGTG |
| bta-miR-1271 | CTTGGCACCTAGTAAGTACTCA |
| bta-miR-1343-3p | CTCCTGGGGCCCGCACTCTC |
| bta-miR-1277 | TACGTAGATATATATGTATTTT |
| bta-miR-1343-5p | TGGGGAGCGGCCCCCGGGCGGG |
| bta-miR-1281 | TCGCCTCCTCCTCTCCC |
| bta-miR-1388-3p | ATCTCAGGTTTGTCAGCCCGCA |
| bta-miR-1282 | TCGTTTGCCTTTTTCTGCTT |
| RNT43 snoRNA | CTTATTGACGGGCGGACAGAAAC |
| bta-miR-1284 | TCTGCACAGACCCTGGCTTTTC |
| Hm/Ms/Rt T1 snRNA | CGACTGCATAATTTGTGGTAGTGG |
| bta-miR-1287 | TGCTGGATCAGTGGTTTGAGTC |
| bta-miR-99b | CACCCGTAGAACCGACCTTGCG |
| bta-miR-1291 | TGGCCCTGACTGAAGACCTGCAGT |

| **Supplementary Table S2.** **Nano-flow cytometry analysis of small extracellular vesicles (sEVs) isolated from uterine fluid.** Flow cytometry was performed to assess the presence of sEV markers (Alix, Syntenin, CD63, and CD9) and the endoplasmic reticulum marker (Calnexin) in extracellular vesicles isolated from uterine fluid (UF-EVs) collected from dairy cows with Low or High negative energy balance (NEB) at 30 and 60 days post-calving (DPC), compared to a negative control (PBS). The number of marker-positive events per microliter (Events/µL) is presented in the first column for each protein, while the total number of events below 300 nm (representing the EV-enriched population) is shown in the second column for each marker.   \| **Events/µL** \| **Alix (PE-H)** \| \| **Syntenin (FITC-H)** \| \| **CD9 (FITC-H)** \| \| **CD63 (FITC-H)** \| \| **Calnexin (FITC-H)** \| \| \| --- \| --- \| --- \| --- \| --- \| --- \| --- \| --- \| --- \| --- \| --- \| \|  \| Marked events (Events/µL) \| <300 nm (all events) \| Marked events (Events/µL) \| <300 nm (all events) \| Marked events (Events/µL) \| <300 nm (all events) \| Marked events (Events/µL) \| <300 nm (all events) \| Marked events (Events/µL) \| <300 nm (all events) \| \| **PBS** \| 6.74 \| 74.09 \| 8.9 \| 393.97 \| 2.76 \| 70.03 \| 5.76 \| 359.38 \| 0.72 \| 351.8 \| \| **Low NEB 30DPC** \| 57.20 \| 1125.39 \| 23.19 \| 4089.44 \| 12.43 \| 1214.32 \| 40.17 \| 5471.68 \| 0.72 \| 744.49 \| \| **High NEB 30DPC** \| 151.29 \| 1010.03 \| 11.54 \| 4660.83 \| 12.36 \| 1062.23 \| 23.13 \| 2437.65 \| 0.64 \| 716.44 \| \| **Low NEB 60DPC** \| 30.10 \| 914.63 \| 15.98 \| 4055.91 \| 10.79 \| 725.13 \| 16.80 \| 4610.63 \| 1.02 \| 1225.68 \| \| **High NEB 60DPC** \| 101.51 \| 940.94 \| 14.65 \| 4506.49 \| 8.75 \| 721.16 \| 23.35 \| 2534.59 \| 0.31 \| 605.64 \|   **Supplementary Table S3:** List of miRNA identified (Cycle Threshold) in UF-EVs from Low and High NEB dairy cows collected at 30 DPC. | | | | | | | |
| --- | --- | --- | --- | --- | --- | --- | --- | --- | --- | --- | --- | --- | --- | --- | --- | --- | --- | --- | --- | --- | --- | --- | --- | --- | --- | --- | --- | --- | --- | --- | --- | --- | --- | --- | --- | --- | --- | --- | --- | --- | --- | --- | --- | --- | --- | --- | --- | --- | --- | --- | --- | --- | --- | --- | --- | --- | --- | --- | --- | --- | --- | --- | --- | --- | --- | --- | --- | --- | --- | --- | --- | --- | --- | --- | --- | --- | --- | --- | --- | --- | --- | --- | --- | --- |
|  |  |  |  |  |  |  |  |
| **MicroRNAs** | **Group - 30 DPC** | | | | | | |
|  | **Low NEB** | | | **High NEB** | | | |
|  | **EVS LU 185** | **EVS LU 212** | **EVS LU 254** | **EVS LU 213** | **EVS LU 233** | **EVS LU 246** | **EVS LU 171** |
| bta-let-7a-3p | 30.934 | 31.503 | 30.057 | 30.691 | 31.950 | 36.248 | 30.144 |
| bta-miR-103 | 26.338 | 26.180 | 27.561 | 27.532 | 28.320 | 28.874 | 26.580 |
| bta-let-7a-5p | 23.235 | 23.240 | 24.654 | 23.623 | 24.835 | 25.888 | 23.054 |
| bta-miR-105a | 31.865 | 31.805 | 33.942 | 33.992 | 31.890 | 34.202 | 32.930 |
| bta-let-7b | 22.071 | 22.291 | 23.133 | 23.212 | 23.489 | 23.941 | 22.182 |
| bta-miR-105b | 33.479 | 32.961 | . | 35.494 | 35.235 | 35.656 | 35.482 |
| bta-let-7c | 22.129 | 22.404 | 23.554 | 22.952 | 23.231 | 24.425 | 21.593 |
| bta-miR-106a | 25.353 | 25.443 | 25.781 | 25.269 | 27.693 | 28.670 | 25.982 |
| bta-let-7d | 24.804 | 24.993 | 26.551 | 24.981 | 25.775 | 27.615 | 26.459 |
| bta-miR-106b | 26.575 | 26.705 | 26.687 | 26.075 | 27.708 | 29.361 | 27.219 |
| bta-let-7e | 22.834 | 22.768 | 24.427 | 23.391 | 24.640 | 25.669 | 22.752 |
| bta-miR-107 | 30.421 | 30.151 | 32.700 | 31.950 | 31.871 | 32.413 | 30.268 |
| bta-let-7f | 25.148 | 24.873 | 26.246 | 25.248 | 26.069 | 27.937 | 25.025 |
| bta-miR-10a | 28.051 | 27.745 | 27.742 | 27.159 | 28.839 | 30.162 | 27.372 |
| bta-let-7g | 25.813 | 25.864 | 25.747 | 26.381 | 27.220 | 28.433 | 25.732 |
| bta-miR-10b | 28.155 | 27.812 | 28.171 | 27.333 | 29.432 | 30.781 | 27.502 |
| bta-let-7i | 26.880 | 26.513 | 25.791 | 27.426 | 28.839 | 29.933 | 26.752 |
| bta-miR-122 | 31.899 | 31.811 | 28.658 | 36.118 | 33.338 | 35.947 | 31.897 |
| bta-miR-1 | . | . | 36.401 | . | . | . | . |
| bta-miR-124a | 29.758 | 29.677 | . | 30.785 | 30.752 | 32.855 | 31.074 |
| bta-miR-100 | 30.330 | 29.010 | 26.639 | 29.411 | 29.226 | 33.873 | 28.731 |
| bta-miR-124b | 29.773 | 29.734 | 36.397 | 30.826 | 31.524 | 31.870 | 32.903 |
| bta-miR-101 | 28.098 | 28.101 | 25.483 | 27.358 | 29.762 | 30.762 | 27.745 |
| bta-miR-125a | 26.555 | 26.352 | 26.952 | 27.016 | 27.860 | 28.740 | 26.214 |
| bta-miR-125b | 25.638 | 24.978 | 23.768 | 25.095 | 26.406 | 28.515 | 24.822 |
| bta-miR-133b | 32.911 | 31.912 | 32.872 | 34.630 | 32.926 | 33.848 | 34.907 |
| bta-miR-126-3p | 31.871 | 29.718 | 26.818 | 30.160 | 32.294 | 35.884 | 29.735 |
| bta-miR-133c | 34.149 | 34.997 | . | 36.651 | . | . | 36.768 |
| bta-miR-126-5p | 33.155 | 30.720 | 27.795 | 31.561 | 31.895 | . | 31.627 |
| bta-miR-134 | 28.829 | 28.837 | 32.496 | 30.833 | 31.363 | 31.347 | 29.764 |
| bta-miR-127 | 27.630 | 27.706 | 28.606 | 25.780 | 27.008 | 28.891 | 29.003 |
| bta-miR-135a | 27.253 | 27.958 | 28.760 | 27.506 | 29.072 | 30.359 | 27.295 |
| bta-miR-128 | 29.698 | 29.502 | 29.626 | 29.859 | 31.851 | 32.553 | 29.579 |
| bta-miR-135b | 28.791 | 28.746 | 29.889 | 28.807 | 30.776 | 30.726 | 28.833 |
| bta-miR-129 | 28.869 | 29.004 | 33.417 | 29.765 | 29.664 | 31.744 | 30.084 |
| bta-miR-136 | . | 33.431 | . | 33.912 | 32.831 | . | 35.586 |
| bta-miR-129-3p | 30.030 | 32.039 | 34.259 | 33.215 | 32.711 | 34.265 | 33.956 |
| bta-miR-137 | . | . | 27.929 | 34.491 | . | . | . |
| bta-miR-129-5p | 28.709 | 29.082 | 32.887 | 29.725 | 29.863 | 31.554 | 30.012 |
| bta-miR-138 | 27.312 | 27.643 | 32.137 | 30.211 | 30.912 | 31.885 | 29.753 |
| bta-miR-130a | 30.019 | 30.337 | 28.316 | 29.770 | 32.638 | 31.819 | 29.646 |
| bta-miR-139 | 29.900 | 29.666 | 28.346 | 30.807 | 30.827 | 31.893 | 29.981 |
| bta-miR-130b | 28.813 | 27.041 | 29.295 | 28.831 | 30.719 | 31.172 | 27.943 |
| bta-miR-140 | 29.795 | 29.037 | 28.336 | 29.363 | 30.088 | 31.294 | 30.039 |
| bta-miR-132 | 31.895 | 30.781 | 30.998 | 31.844 | 31.470 | 33.745 | 33.062 |
| bta-miR-141 | 25.654 | 26.135 | 26.069 | 24.928 | 26.522 | 27.996 | 26.388 |
| bta-miR-133a | 27.260 | 28.159 | 30.581 | 29.890 | 29.215 | 31.219 | 29.497 |
| bta-miR-142-3p | 35.016 | 33.966 | 32.098 | 33.105 | 36.809 | 36.880 | 33.072 |
| bta-miR-142-5p | 31.786 | 29.892 | 28.749 | 29.671 | 32.068 | 34.953 | 30.757 |
| bta-miR-151-3p | 28.106 | 28.339 | 29.413 | 29.407 | 30.195 | 30.542 | 28.816 |
| bta-miR-143 | 30.229 | 29.795 | 27.251 | 29.748 | 31.760 | 32.865 | 29.508 |
| bta-miR-151-5p | 27.927 | 27.799 | 27.858 | 27.818 | 29.367 | 31.223 | 27.748 |
| bta-miR-144 | . | . | 35.059 | . | . | . | . |
| bta-miR-152 | 31.848 | 31.766 | 31.864 | 33.314 | 32.539 | 33.838 | 31.950 |
| bta-miR-145 | 27.824 | 26.506 | 24.509 | 27.272 | 28.266 | 30.038 | 27.066 |
| bta-miR-153 | 33.971 | 35.615 | 32.512 | . | . | . | 30.802 |
| bta-miR-146a | 33.963 | 35.126 | 33.860 | 34.550 | . | . | 34.996 |
| bta-miR-154a | 33.570 | 34.383 | 32.031 | 34.561 | 35.972 | 35.176 | 34.413 |
| bta-miR-146b | 34.535 | 33.142 | 33.469 | 34.956 | 33.829 | . | 33.949 |
| bta-miR-154b | 33.988 | 32.863 | 32.761 | 32.935 | 35.070 | 36.965 | 34.377 |
| bta-miR-147 | 29.798 | 29.827 | 30.567 | 30.802 | 32.513 | 32.838 | 30.086 |
| bta-miR-154c | 35.203 | 32.590 | 32.283 | . | . | . | 33.966 |
| bta-miR-148a | 26.969 | 26.414 | 25.127 | 26.386 | 27.994 | 28.906 | 26.032 |
| bta-miR-155 | 27.717 | 27.312 | 27.288 | 27.772 | . | 31.527 | 27.519 |
| bta-miR-148b | 26.970 | 26.395 | 25.436 | 26.412 | 27.782 | 29.070 | 26.156 |
| bta-miR-15a | 27.338 | 26.614 | 26.822 | 26.964 | 29.382 | 29.956 | 26.974 |
| bta-miR-149-3p | 24.671 | 24.475 | 27.544 | 23.982 | 25.934 | 26.988 | 25.785 |
| bta-miR-15b | 27.824 | 27.432 | 28.756 | 28.474 | 29.262 | 30.434 | 27.638 |
| bta-miR-149-5p | 27.100 | 26.896 | 28.300 | 27.267 | 27.415 | 28.494 | 27.522 |
| bta-miR-16a | 26.592 | 26.117 | 25.477 | 26.035 | 27.799 | 29.298 | 26.025 |
| bta-miR-150 | 31.808 | 29.207 | 29.798 | 31.883 | 31.018 | 36.099 | 30.084 |
| bta-miR-16b | 27.244 | 26.755 | 25.785 | 26.935 | 28.796 | 30.018 | 26.860 |
| bta-miR-17-3p | 31.356 | 29.790 | 30.540 | 30.204 | 31.617 | 31.766 | 31.529 |
| bta-miR-188 | 30.154 | 30.367 | 31.808 | 31.336 | 30.620 | 33.285 | 31.882 |
| bta-miR-17-5p | 29.356 | 29.121 | 31.765 | 29.329 | 30.508 | 31.784 | 29.388 |
| bta-miR-18a | 28.816 | 29.125 | 28.697 | 29.117 | 30.892 | 31.863 | 28.740 |
| bta-miR-181a | 28.724 | 28.229 | 29.598 | 28.349 | 30.913 | 30.909 | 28.375 |
| bta-miR-18b | 30.309 | 30.538 | 30.813 | 30.792 | 34.366 | 33.930 | 31.699 |
| bta-miR-181b | 27.958 | 28.069 | 29.562 | 28.118 | 30.593 | 30.534 | 28.256 |
| bta-miR-190a | 31.660 | 31.840 | 32.086 | 33.438 | 33.618 | 35.084 | 33.950 |
| bta-miR-181c | 29.785 | 29.368 | 30.738 | 29.525 | 31.843 | 32.402 | 29.115 |
| bta-miR-190b | 31.113 | 31.703 | 30.573 | 30.178 | 33.146 | 31.880 | 29.793 |
| bta-miR-181d | 27.975 | 27.878 | 29.829 | 28.181 | 30.403 | 30.026 | 28.254 |
| bta-miR-191 | 27.501 | 27.593 | 27.706 | 27.774 | 28.721 | 30.551 | 27.614 |
| bta-miR-182 | 28.008 | 27.495 | 29.572 | 27.779 | 30.756 | 29.822 | 27.750 |
| bta-miR-192 | 30.726 | 30.368 | 32.718 | 31.153 | 31.234 | 32.808 | 32.057 |
| bta-miR-183 | 30.072 | 28.855 | 30.853 | 30.235 | 32.854 | 32.686 | 29.362 |
| bta-miR-193a | 33.166 | 32.889 | . | 34.942 | 34.173 | . | 34.527 |
| bta-miR-184 | 31.524 | 30.740 | 32.350 | 32.843 | 35.976 | 35.111 | 31.797 |
| bta-miR-193a-3p | 30.997 | 30.671 | 28.519 | 30.532 | 31.228 | 32.385 | 31.894 |
| bta-miR-185 | 29.412 | 28.929 | 28.817 | 29.786 | 30.319 | 31.592 | 29.627 |
| bta-miR-193a-5p | 28.707 | 28.439 | 30.521 | 29.855 | 30.259 | 30.894 | 29.538 |
| bta-miR-186 | 28.759 | 28.697 | 26.762 | 27.625 | 29.176 | 31.882 | 28.909 |
| bta-miR-193b | 31.421 | 31.376 | 33.488 | 32.919 | 31.824 | 33.318 | 31.808 |
| bta-miR-187 | 29.140 | 28.740 | 31.115 | 28.977 | 29.529 | 31.339 | 30.456 |
| bta-miR-194 | 30.048 | 29.654 | 29.789 | 29.391 | 31.862 | 32.924 | 30.904 |
| bta-miR-195 | 29.800 | 28.830 | 26.786 | 29.691 | 30.901 | 32.295 | 28.089 |
| bta-miR-200c | 24.830 | 24.802 | 25.792 | 24.637 | 25.879 | 27.158 | 24.475 |
| bta-miR-196a | 30.118 | 29.868 | 29.636 | 30.018 | 31.010 | 33.054 | 28.749 |
| bta-miR-202 | . | . | . | 36.641 | . | . | . |
| bta-miR-196b | 30.284 | 29.603 | 29.666 | 30.638 | 31.866 | 33.989 | 28.962 |
| bta-miR-204 | 28.740 | 28.924 | 27.834 | 28.969 | 29.230 | 31.563 | 28.746 |
| bta-miR-197 | 27.085 | 27.209 | 29.837 | 28.670 | 28.314 | 29.582 | 27.865 |
| bta-miR-205 | 28.903 | 28.550 | 28.571 | 27.827 | 30.330 | 30.573 | 28.560 |
| bta-miR-199a-3p | 28.284 | 26.534 | 24.194 | 27.767 | 28.028 | 32.746 | 26.517 |
| bta-miR-206 | 30.813 | 30.787 | 35.341 | 30.931 | 32.716 | 33.319 | 30.990 |
| bta-miR-199a-5p | 28.671 | 27.396 | 24.561 | 27.486 | 29.203 | 32.888 | 27.817 |
| bta-miR-208a | 35.324 | 35.002 | . | 35.033 | . | 35.357 | 35.319 |
| bta-miR-199b | 29.729 | 27.838 | 25.228 | 28.275 | 29.598 | 33.931 | 28.127 |
| bta-miR-208b | 35.232 | 34.908 | . | . | . | . | 34.880 |
| bta-miR-199c | 27.590 | 26.118 | 23.999 | 27.585 | 27.619 | 31.783 | 26.107 |
| bta-miR-20a | 25.815 | 25.825 | 26.649 | 25.701 | 27.713 | 28.783 | 25.938 |
| bta-miR-19a | 24.807 | 24.380 | 24.772 | 25.251 | 27.381 | 28.547 | 24.718 |
| bta-miR-20b | 26.521 | 26.656 | 27.006 | 25.677 | 28.762 | 28.975 | 26.796 |
| bta-miR-19b | 24.792 | 24.306 | 24.752 | 25.353 | 27.079 | 28.259 | 24.692 |
| bta-miR-21-3p | 30.076 | 30.143 | 31.801 | 30.786 | 30.816 | 31.856 | 30.547 |
| bta-miR-200a | 25.148 | 25.420 | 25.226 | 24.434 | 26.691 | 28.000 | 25.436 |
| bta-miR-21-5p | 29.793 | 29.452 | 30.737 | 30.799 | 31.070 | 32.793 | 29.828 |
| bta-miR-200b | 23.068 | 23.382 | 23.771 | 22.652 | 24.218 | 25.744 | 23.125 |
| bta-miR-210 | 26.657 | 25.171 | 28.580 | 26.577 | 26.720 | 28.775 | 25.824 |
| bta-miR-211 | 28.052 | 27.887 | 27.809 | 26.082 | 25.978 | 30.759 | 27.906 |
| bta-miR-22-5p | 30.754 | 30.733 | 29.939 | 31.540 | 32.472 | 33.948 | 30.978 |
| bta-miR-212 | 33.960 | 33.557 | 36.532 | . | 34.800 | . | 33.977 |
| bta-miR-221 | 28.257 | 28.066 | 26.985 | 28.737 | 29.587 | 31.525 | 28.453 |
| bta-miR-214 | 24.593 | 24.109 | 26.903 | 23.774 | 25.643 | 26.000 | 24.545 |
| bta-miR-222 | 25.775 | 26.074 | 27.028 | 27.853 | 28.236 | 28.725 | 26.579 |
| bta-miR-215 | 32.537 | 31.996 | 31.748 | 32.114 | 33.681 | . | 33.035 |
| bta-miR-223 | 29.340 | 29.297 | 27.138 | 26.928 | 30.827 | 35.945 | 31.260 |
| bta-miR-216a | 30.779 | 30.838 | 33.889 | 31.880 | 31.851 | 32.902 | 31.770 |
| bta-miR-224 | 27.891 | 27.589 | 29.653 | 28.566 | 31.122 | 31.173 | 28.353 |
| bta-miR-216b | 33.895 | 32.851 | . | . | . | 35.952 | 34.963 |
| bta-miR-23a | 24.272 | 24.313 | 24.366 | 24.294 | 25.219 | 26.997 | 24.099 |
| bta-miR-217 | 31.859 | 30.916 | . | 32.859 | 34.218 | 35.044 | 31.684 |
| bta-miR-23b-3p | 25.817 | 25.789 | 26.917 | 26.081 | 26.546 | 26.986 | 25.775 |
| bta-miR-218 | 31.792 | 30.778 | 30.834 | 31.595 | 35.896 | 36.041 | 29.797 |
| bta-miR-23b-5p | 30.888 | 31.834 | 34.520 | 34.371 | 32.940 | 33.779 | 32.441 |
| bta-miR-219 | 28.787 | 29.344 | 32.097 | 30.267 | 29.516 | 31.481 | 30.181 |
| bta-miR-24 | 33.998 | 35.881 | 34.518 | 33.865 | 34.700 | 36.820 | 34.895 |
| bta-miR-219-3p | 29.804 | 30.117 | 34.987 | 31.138 | 30.812 | 32.356 | 30.088 |
| bta-miR-24-3p | 24.711 | 24.947 | 25.062 | 25.640 | 26.731 | 27.180 | 24.761 |
| bta-miR-219-5p | 34.914 | 33.939 | . | 36.453 | . | . | . |
| bta-miR-25 | 26.006 | 25.762 | 26.756 | 26.301 | 27.465 | 28.297 | 26.187 |
| bta-miR-22-3p | 3.717 | 3.355 | 25.464 | . | 3.146 | . | . |
| bta-miR-26a | 24.735 | 24.618 | 23.595 | 24.159 | 25.822 | 27.109 | 23.908 |
| bta-miR-26b | 26.570 | 26.220 | 25.222 | 25.872 | 27.364 | 29.414 | 25.619 |
| bta-miR-29d-3p | 27.424 | 28.075 | 26.380 | 27.211 | 28.541 | 30.425 | 27.539 |
| bta-miR-26c | . | . | . | 36.785 | . | 36.075 | . |
| bta-miR-29d-5p | 31.253 | 30.742 | 30.039 | 30.821 | 31.582 | 33.183 | 30.863 |
| bta-miR-27a-3p | 25.182 | 25.504 | 24.043 | 24.902 | 26.584 | 29.025 | 25.328 |
| bta-miR-29e | 29.656 | 29.188 | 28.801 | 29.344 | 31.403 | 31.723 | 29.752 |
| bta-miR-27a-5p | 31.207 | 31.330 | 35.094 | 31.031 | 30.494 | 32.947 | 31.628 |
| bta-miR-301a | 32.663 | 32.661 | 32.548 | 32.935 | 34.386 | 34.496 | 30.086 |
| bta-miR-27b | 26.252 | 26.526 | 24.624 | 25.585 | 26.795 | 28.327 | 26.088 |
| bta-miR-301b | 32.123 | 32.204 | 32.927 | 32.934 | 35.985 | . | 32.264 |
| bta-miR-28 | 30.128 | 29.873 | 29.835 | 30.793 | 32.068 | 32.644 | 29.805 |
| bta-miR-302a | 34.787 | 33.484 | 36.694 | 34.171 | . | 35.845 | 34.416 |
| bta-miR-296-3p | 27.769 | 26.906 | 29.346 | 26.852 | 28.817 | 30.007 | 27.669 |
| bta-miR-302b | . | . | . | . | . | . | . |
| bta-miR-296-5p | 29.702 | 29.423 | 31.872 | 30.254 | 31.232 | 30.748 | 29.898 |
| bta-miR-302c | 32.026 | 33.949 | . | 34.193 | 34.951 | 34.530 | 35.126 |
| bta-miR-299 | 34.244 | 32.910 | 32.098 | 36.527 | 34.095 | . | 32.852 |
| bta-miR-302d | . | 35.040 | . | 35.489 | . | . | . |
| bta-miR-29a | 25.157 | 25.026 | 23.909 | 24.733 | 26.148 | 27.885 | 24.671 |
| bta-miR-3064 | 30.840 | 31.456 | . | 35.051 | 32.806 | 31.990 | 32.573 |
| bta-miR-29b | 26.969 | 26.238 | 25.814 | 26.492 | 28.147 | 29.410 | 26.291 |
| bta-miR-30a-5p | 27.678 | 27.475 | 27.440 | 27.110 | 27.763 | 29.206 | 27.809 |
| bta-miR-29c | 25.193 | 24.990 | 23.761 | 24.702 | 26.214 | 28.208 | 24.601 |
| bta-miR-30b-3p | 32.885 | 33.708 | 34.004 | 34.857 | 33.335 | 35.054 | 33.007 |
| bta-miR-30b-5p | 27.409 | 27.765 | 27.129 | 26.791 | 27.746 | 30.041 | 27.489 |
| bta-miR-328 | 28.956 | 28.672 | 30.803 | 28.418 | 29.808 | 31.821 | 29.154 |
| bta-miR-30c | 27.064 | 26.961 | 27.750 | 26.738 | 27.617 | 29.589 | 26.853 |
| bta-miR-329a | . | 36.593 | 34.597 | . | . | . | . |
| bta-miR-30d | 27.574 | 27.617 | 27.664 | 27.269 | 28.028 | 29.707 | 27.635 |
| bta-miR-329b | 33.863 | 33.906 | 35.109 | 34.659 | 33.875 | . | 33.937 |
| bta-miR-30e-5p | 27.653 | 27.680 | 27.460 | 27.280 | 28.370 | 29.656 | 27.837 |
| bta-miR-330 | 28.468 | 29.233 | 34.279 | 30.253 | 30.184 | 31.929 | 29.905 |
| bta-miR-30f | 27.797 | 27.527 | 28.877 | 27.453 | 28.301 | 30.366 | 27.792 |
| bta-miR-331-3p | 28.746 | 28.428 | 29.609 | 29.365 | 30.192 | 31.574 | 29.019 |
| bta-miR-31 | 25.156 | 25.537 | 27.040 | 25.817 | 26.395 | 27.213 | 25.424 |
| bta-miR-331-5p | 31.112 | 30.810 | 31.785 | 30.812 | 30.986 | 32.608 | 30.855 |
| bta-miR-32 | 33.539 | 32.021 | 32.736 | 31.584 | 33.950 | 35.234 | 31.656 |
| bta-miR-335 | 25.959 | 26.606 | 26.815 | 26.082 | 26.451 | 25.822 | 26.411 |
| bta-miR-320a | 25.339 | 25.382 | 25.666 | 24.874 | 26.045 | 27.496 | 25.237 |
| bta-miR-338 | 36.220 | 33.997 | 30.760 | 33.513 | 35.443 | . | 33.970 |
| bta-miR-320b | 28.281 | 28.409 | 29.403 | 27.671 | 28.847 | 29.446 | 28.675 |
| bta-miR-339a | 27.661 | 27.103 | 27.752 | 27.833 | 28.231 | 28.635 | 27.791 |
| bta-miR-323 | 32.404 | 30.879 | 33.605 | 31.908 | 31.716 | 32.912 | 30.870 |
| bta-miR-339b | 26.280 | 26.074 | 25.592 | 26.013 | 26.677 | 27.629 | 26.117 |
| bta-miR-324 | 29.691 | 29.605 | 30.636 | 29.832 | 30.247 | 31.003 | 29.720 |
| bta-miR-33a | 31.215 | 29.046 | 27.752 | 27.830 | 30.087 | 31.313 | 29.531 |
| bta-miR-326 | 27.669 | 27.559 | 30.043 | 28.451 | 28.603 | 29.728 | 28.653 |
| bta-miR-33b | 31.863 | 30.799 | 28.499 | 30.036 | 31.039 | 33.955 | 31.747 |
| bta-miR-340 | . | 33.853 | 33.299 | 33.896 | . | 36.099 | 35.765 |
| bta-miR-365-3p | 29.200 | 28.925 | 29.000 | 29.969 | 30.823 | 31.679 | 28.822 |
| bta-miR-342 | 28.321 | 27.233 | 28.931 | 28.176 | 28.159 | 30.790 | 28.100 |
| bta-miR-365-5p | 29.713 | 29.877 | 33.139 | 29.938 | 31.410 | 31.623 | 30.440 |
| bta-miR-345-3p | 29.748 | 29.837 | 31.339 | 30.359 | 30.359 | 31.842 | 30.563 |
| bta-miR-367 | 36.864 | 34.825 | . | . | . | . | 36.342 |
| bta-miR-345-5p | 27.157 | 27.755 | 28.648 | 26.428 | 27.804 | 29.661 | 28.503 |
| bta-miR-369-3p | . | 34.980 | 30.029 | 34.839 | 34.746 | . | 34.954 |
| bta-miR-346 | 24.749 | 25.193 | 31.781 | 27.692 | 27.188 | 28.152 | 25.958 |
| bta-miR-369-5p | . | . | 33.885 | . | . | . | . |
| bta-miR-34a | 26.414 | 25.824 | 26.803 | 27.497 | 29.029 | 29.027 | 26.173 |
| bta-miR-370 | 28.840 | 29.174 | 32.431 | 30.469 | 30.504 | 30.827 | 29.909 |
| bta-miR-34b | 27.191 | 26.735 | 24.642 | 26.134 | 28.295 | 29.731 | 25.382 |
| bta-miR-371 | 31.676 | 31.616 | 33.077 | 30.745 | 32.043 | 33.752 | 32.922 |
| bta-miR-34c | 26.846 | 26.677 | 24.558 | 25.890 | 28.468 | 29.601 | 25.200 |
| bta-miR-374a | 28.013 | 27.760 | 27.868 | 27.494 | 29.455 | 30.800 | 26.855 |
| bta-miR-361 | 27.090 | 26.742 | 27.161 | 26.786 | 28.743 | 29.221 | 27.143 |
| bta-miR-374b | 28.993 | 28.791 | 28.885 | 28.840 | 30.158 | 31.874 | 28.510 |
| bta-miR-362-3p | 31.814 | 32.893 | 30.436 | 32.665 | 32.900 | . | 32.638 |
| bta-miR-375 | 26.205 | 25.710 | 25.300 | 26.576 | 26.915 | 29.630 | 24.549 |
| bta-miR-362-5p | 31.062 | 31.480 | 31.849 | 30.908 | 32.044 | 33.405 | 32.098 |
| bta-miR-376a | 33.246 | 33.423 | 32.458 | 35.796 | . | 36.137 | . |
| bta-miR-363 | 33.091 | 33.913 | 33.166 | 33.188 | 34.232 | 35.679 | 34.132 |
| bta-miR-376b | . | 33.951 | 31.531 | 33.612 | . | . | 35.449 |
| bta-miR-376c | 34.962 | 35.017 | 30.825 | 35.996 | . | . | 33.112 |
| bta-miR-382 | 34.274 | 33.309 | 31.852 | 32.972 | 33.124 | . | 33.102 |
| bta-miR-376d | 34.943 | 34.443 | 29.524 | 36.287 | 33.726 | . | 34.063 |
| bta-miR-383 | 30.329 | 30.605 | 35.935 | 31.614 | 32.045 | 32.004 | 32.059 |
| bta-miR-376e | 35.396 | 33.507 | 29.768 | 34.169 | 33.495 | . | 33.030 |
| bta-miR-409a | 33.922 | 32.987 | 33.037 | 34.310 | 34.603 | 36.014 | 33.820 |
| bta-miR-377 | 34.026 | 34.846 | 31.406 | 32.247 | 34.962 | 34.754 | 32.858 |
| bta-miR-409b | 33.262 | 33.971 | . | . | 35.955 | 34.665 | 35.024 |
| bta-miR-378 | 27.264 | 27.094 | 28.364 | 28.772 | 28.762 | 28.912 | 27.749 |
| bta-miR-410 | 30.928 | 29.531 | 31.852 | 31.892 | 31.073 | 32.121 | 32.875 |
| bta-miR-378b | 27.381 | 27.402 | 28.565 | 28.601 | 28.986 | 28.921 | 27.779 |
| bta-miR-411a | 34.968 | 31.854 | 29.877 | 32.210 | 33.703 | . | 32.944 |
| bta-miR-378c | 31.879 | 32.768 | 32.904 | 34.546 | 33.921 | 34.745 | 32.679 |
| bta-miR-411b | 31.981 | 32.578 | 35.308 | 36.928 | . | 34.972 | 32.843 |
| bta-miR-378d | 29.949 | 30.766 | 31.796 | 30.070 | 31.903 | 31.851 | 31.914 |
| bta-miR-411c-3p | 32.039 | 32.268 | 33.241 | . | 35.597 | . | 35.512 |
| bta-miR-379 | 32.944 | 30.241 | 29.902 | 32.720 | 32.979 | 34.654 | 31.378 |
| bta-miR-411c-5p | 35.875 | . | 32.931 | 35.704 | . | . | . |
| bta-miR-380-3p | 32.056 | 31.015 | 29.772 | 32.945 | 32.222 | 33.947 | 33.603 |
| bta-miR-412 | 32.849 | 32.776 | 34.106 | 34.657 | 34.630 | 36.373 | 32.901 |
| bta-miR-380-5p | 33.921 | 34.042 | 35.971 | 35.002 | 34.573 | 35.470 | 34.909 |
| bta-miR-421 | 27.544 | 26.644 | 28.361 | 23.471 | 25.986 | 30.784 | 25.913 |
| bta-miR-381 | 29.912 | 30.777 | 30.448 | 32.165 | 31.858 | 33.294 | 32.024 |
| bta-miR-423-3p | 26.873 | 27.032 | 29.083 | 27.810 | 28.568 | 28.896 | 27.426 |
| bta-miR-423-5p | 26.829 | 26.735 | 27.261 | 27.151 | 27.823 | 28.591 | 26.784 |
| bta-miR-449c | 29.447 | 29.677 | 31.983 | 28.940 | 30.223 | 31.796 | 29.173 |
| bta-miR-424-3p | 31.859 | 31.156 | 32.178 | 32.629 | 32.875 | 34.875 | 31.601 |
| bta-miR-449d | 27.476 | 27.879 | 33.013 | 29.142 | 29.041 | 29.738 | 29.134 |
| bta-miR-424-5p | 27.749 | 27.805 | 26.599 | 28.629 | 29.162 | 31.840 | 28.775 |
| bta-miR-450a | 29.393 | 30.354 | 28.640 | 29.976 | 30.194 | 32.904 | 29.809 |
| bta-miR-425-3p | 23.039 | 22.935 | 22.781 | 19.841 | 21.778 | 25.669 | 24.597 |
| bta-miR-450b | 30.693 | 32.601 | 29.909 | 31.572 | 31.955 | . | 31.420 |
| bta-miR-425-5p | 28.537 | 28.989 | 28.779 | 28.817 | 29.974 | 31.372 | 29.310 |
| bta-miR-451 | 29.025 | 27.681 | 21.735 | 28.028 | 30.005 | 35.746 | 28.518 |
| bta-miR-429 | 27.658 | 27.871 | 28.130 | 27.632 | 28.558 | 30.156 | 27.139 |
| bta-miR-452 | 30.576 | 30.547 | 33.721 | 32.826 | 31.880 | 33.844 | 31.478 |
| bta-miR-431 | 28.278 | 28.314 | 32.724 | 30.103 | 29.312 | 30.276 | 29.546 |
| bta-miR-4523 | 29.379 | 29.590 | 32.964 | 30.982 | 30.815 | 31.909 | 30.453 |
| bta-miR-432 | 30.516 | 30.668 | 31.928 | 31.844 | 31.012 | 32.853 | 31.290 |
| bta-miR-453 | 30.585 | 30.809 | 31.856 | 31.440 | 31.716 | 31.398 | 30.882 |
| bta-miR-433 | 29.910 | 29.934 | 32.091 | 31.367 | 31.397 | 32.550 | 31.562 |
| bta-miR-454 | 31.885 | 31.849 | 32.865 | 32.307 | 35.056 | . | 30.788 |
| bta-miR-448 | 34.042 | 33.934 | . | 36.373 | 35.542 | 34.400 | 34.995 |
| bta-miR-455-3p | 33.178 | 32.560 | 29.775 | 32.551 | 33.429 | . | 32.856 |
| bta-miR-449a | 27.399 | 26.952 | 28.131 | 26.095 | 28.082 | 29.972 | 25.749 |
| bta-miR-455-5p | 31.773 | 30.482 | 28.449 | 30.652 | 32.907 | 34.712 | 30.332 |
| bta-miR-449b | 30.085 | 28.815 | 30.756 | 28.606 | 29.929 | 32.003 | 28.372 |
| bta-miR-483 | 30.649 | 30.718 | 34.032 | 31.847 | 31.710 | 34.982 | 32.678 |
| bta-miR-484 | 27.790 | 27.434 | 29.146 | 28.155 | 29.062 | 29.378 | 27.812 |
| bta-miR-496 | 33.228 | 32.854 | 33.663 | 33.996 | 34.884 | 34.694 | 33.483 |
| bta-miR-485 | 33.528 | 33.855 | 33.569 | 33.893 | 34.189 | 35.845 | 33.552 |
| bta-miR-497 | 28.736 | 28.551 | 26.855 | 29.716 | 30.907 | 31.337 | 28.409 |
| bta-miR-486 | 25.842 | 25.619 | 24.934 | 22.846 | 23.858 | 27.944 | 25.299 |
| bta-miR-499 | 29.735 | 33.100 | 32.296 | 30.508 | 31.456 | 33.053 | 30.709 |
| bta-miR-487a | 31.901 | 31.788 | 32.798 | 33.850 | 35.346 | 34.463 | 33.890 |
| bta-miR-500 | 29.564 | 29.797 | 29.166 | 28.577 | 29.605 | 30.353 | 29.896 |
| bta-miR-487b | 32.761 | 32.849 | 30.247 | 33.477 | 35.008 | 34.017 | 33.321 |
| bta-miR-502a | 31.036 | 31.453 | 29.086 | 29.531 | 31.885 | 30.839 | 29.838 |
| bta-miR-488 | 31.613 | 31.837 | 35.374 | 33.718 | 34.724 | 33.530 | 32.881 |
| bta-miR-502b | 31.142 | 31.730 | 31.893 | 31.885 | 32.263 | 34.248 | 32.017 |
| bta-miR-489 | 36.947 | 34.852 | 35.385 | 32.938 | 33.650 | 35.230 | 35.056 |
| bta-miR-503-3p | 26.369 | 27.387 | 29.980 | 27.833 | 28.049 | 29.629 | 28.293 |
| bta-miR-490 | 29.299 | 29.524 | 31.876 | 30.749 | 31.484 | 31.627 | 30.746 |
| bta-miR-503-5p | 30.368 | 31.182 | 31.149 | 32.047 | 32.104 | 32.888 | 30.954 |
| bta-miR-491 | 31.043 | 30.820 | 31.662 | 31.838 | 32.553 | 32.300 | 30.906 |
| bta-miR-504 | 30.096 | 30.569 | 34.531 | 31.654 | 32.347 | 32.922 | 31.021 |
| bta-miR-493 | 26.822 | 27.109 | 29.035 | 24.743 | 26.601 | 28.761 | 28.191 |
| bta-miR-505 | 27.509 | 27.186 | 28.683 | 27.051 | 27.722 | 28.774 | 27.792 |
| bta-miR-494 | 13.883 | 14.574 | 17.551 | 13.988 | 16.210 | 16.903 | 15.350 |
| bta-miR-532 | 31.506 | 31.934 | 30.282 | 31.791 | 32.416 | 33.933 | 31.902 |
| bta-miR-495 | 34.705 | 33.166 | 30.744 | 35.423 | 34.906 | 36.037 | 33.539 |
| bta-miR-539 | 33.990 | 33.989 | . | 34.380 | 36.908 | 36.357 | . |
| bta-miR-541 | 28.528 | 29.129 | 32.597 | 29.798 | 29.761 | 30.235 | 30.160 |
| bta-miR-582 | 34.898 | 33.422 | 33.016 | 32.876 | 32.343 | 33.958 | 32.303 |
| bta-miR-542-5p | 32.972 | 32.529 | 31.879 | 31.849 | 33.170 | 34.760 | 31.896 |
| bta-miR-584 | 26.312 | 27.748 | 28.794 | 26.560 | 27.784 | 28.159 | 28.049 |
| bta-miR-543 | 31.927 | 32.904 | 32.190 | 34.331 | 33.381 | 33.118 | 32.722 |
| bta-miR-592 | 30.855 | 30.827 | 30.851 | 29.694 | 33.556 | 32.084 | 29.801 |
| bta-miR-544a | 34.920 | . | 31.877 | . | . | . | 35.239 |
| bta-miR-599 | . | 35.346 | 30.897 | . | . | . | . |
| bta-miR-544b | 34.283 | 32.856 | . | . | 33.903 | 35.266 | . |
| bta-miR-615 | 6.464 | 6.786 | 6.256 | 6.482 | 6.763 | 6.352 | 6.526 |
| bta-miR-545-3p | 32.505 | 32.728 | 32.801 | 32.896 | 35.612 | 32.874 | 33.851 |
| bta-miR-628 | . | 36.134 | . | 34.592 | . | . | . |
| bta-miR-545-5p | 33.606 | 33.068 | 32.109 | 32.688 | 33.098 | . | 34.109 |
| bta-miR-631 | 26.231 | 25.820 | 29.733 | 25.999 | 28.310 | 28.636 | 28.336 |
| bta-miR-551a | 34.384 | 34.409 | . | . | 36.143 | 35.971 | 34.569 |
| bta-miR-652 | 28.696 | 28.770 | 28.996 | 29.253 | 30.210 | 30.654 | 29.016 |
| bta-miR-551b | 32.299 | 32.789 | . | 32.785 | 34.739 | 36.061 | 34.242 |
| bta-miR-653 | . | . | 32.020 | . | . | . | . |
| bta-miR-562 | 33.992 | 32.893 | 34.456 | 34.829 | 35.926 | 34.651 | 33.827 |
| bta-miR-654 | 20.484 | 21.107 | 21.121 | 20.598 | 21.215 | 20.607 | 20.755 |
| bta-miR-568 | 32.922 | 32.895 | 32.002 | 32.889 | 33.077 | 32.850 | 32.764 |
| bta-miR-655 | . | . | 33.181 | . | . | . | 35.990 |
| bta-miR-574 | 23.349 | 23.330 | 27.094 | 24.416 | 24.731 | 26.136 | 24.121 |
| bta-miR-656 | 35.444 | 34.960 | 31.943 | . | 36.878 | . | 35.058 |
| bta-miR-658 | 30.008 | 30.513 | 29.273 | 27.408 | 30.804 | 30.877 | 30.530 |
| bta-miR-758 | 31.901 | 32.691 | 33.681 | 34.909 | 33.901 | 33.320 | 34.002 |
| bta-miR-660 | 30.922 | 30.741 | 29.937 | 30.592 | 32.442 | 32.684 | 30.785 |
| bta-miR-759 | . | . | . | . | . | . | . |
| bta-miR-664a | 27.006 | 27.746 | 31.898 | 28.459 | 29.182 | 29.777 | 28.019 |
| bta-miR-760-3p | 28.350 | 29.219 | 32.323 | 29.903 | 30.708 | 30.003 | 29.531 |
| bta-miR-664b | 24.783 | 24.881 | 24.111 | 24.772 | 25.480 | 24.794 | 24.749 |
| bta-miR-760-5p | 26.579 | 27.027 | 31.659 | 28.191 | 28.591 | 28.745 | 27.951 |
| bta-miR-665 | 25.715 | 25.003 | 28.224 | 25.234 | 25.743 | 28.677 | 25.744 |
| bta-miR-761 | 30.731 | 31.799 | 33.955 | 33.368 | 33.904 | 31.696 | 32.442 |
| bta-miR-669 | 25.513 | 25.797 | 29.829 | 27.055 | 27.848 | 28.281 | 26.702 |
| bta-miR-763 | 27.851 | 28.693 | 34.642 | 30.588 | 30.375 | 30.574 | 29.766 |
| bta-miR-670 | 35.053 | . | 29.720 | 34.975 | 36.072 | 35.062 | . |
| bta-miR-764 | 30.995 | 31.405 | 34.166 | 31.958 | 32.962 | 32.867 | 32.533 |
| bta-miR-671 | 28.734 | 28.856 | 31.603 | 29.062 | 29.739 | 30.862 | 29.795 |
| bta-miR-767 | 31.842 | 32.008 | 36.435 | 33.879 | 33.638 | 33.842 | 33.874 |
| bta-miR-677 | 28.596 | 27.178 | 32.912 | 29.976 | 29.835 | 30.178 | 27.903 |
| bta-miR-769 | 30.979 | 30.805 | 31.846 | 30.739 | 31.798 | 32.828 | 30.929 |
| bta-miR-7 | 30.429 | 30.744 | 32.859 | 31.898 | 33.014 | 34.707 | 29.976 |
| bta-miR-873 | 30.542 | 31.853 | . | 34.959 | 33.433 | 33.078 | 31.892 |
| bta-miR-708 | 30.705 | 31.816 | 31.873 | 31.888 | 33.979 | 34.185 | 30.490 |
| bta-miR-874 | 26.810 | 27.447 | 29.721 | 26.486 | 27.717 | 28.761 | 28.402 |
| bta-miR-744 | 29.125 | 28.755 | 32.662 | 31.242 | 32.672 | 30.239 | 29.235 |
| bta-miR-875 | 34.308 | 35.030 | 29.760 | 33.923 | . | . | 35.695 |
| bta-miR-876 | 35.933 | 34.567 | 33.823 | 35.374 | . | 34.938 | 35.839 |
| bta-miR-98 | 29.692 | 29.348 | 30.446 | 29.665 | 31.835 | 32.090 | 29.113 |
| bta-miR-877 | 27.222 | 27.420 | 29.852 | 27.282 | 27.873 | 29.214 | 28.020 |
| bta-miR-99a-3p | 35.046 | 32.921 | 30.105 | 33.397 | 36.231 | 36.069 | 33.048 |
| bta-miR-885 | 29.439 | 29.390 | 31.621 | 29.992 | 29.838 | 31.025 | 29.945 |
| bta-miR-99a-5p | 30.651 | 28.798 | 25.289 | 29.248 | 29.002 | 35.367 | 28.558 |
| bta-miR-9-3p | . | . | 32.925 | . | . | . | . |
| bta-miR-9-5p | 28.493 | 31.464 | 32.935 | 30.020 | 30.790 | 32.799 | 30.658 |
| bta-miR-1179 | . | 35.916 | 32.962 | 35.263 | 34.920 | . | . |
| bta-miR-92a | 24.587 | 24.278 | 26.412 | 25.303 | 25.755 | 26.732 | 24.660 |
| bta-miR-1185 | . | 34.912 | 32.895 | . | 36.012 | . | 35.680 |
| bta-miR-92b | 24.330 | 23.719 | 25.810 | 23.323 | 24.952 | 26.763 | 24.269 |
| bta-miR-1193 | 30.972 | 31.864 | 33.486 | 31.876 | 33.154 | 32.751 | 32.825 |
| bta-miR-93 | 26.283 | 26.186 | 26.752 | 26.236 | 27.839 | 28.770 | 26.536 |
| bta-miR-1197 | . | . | . | . | . | . | . |
| bta-miR-935 | 17.804 | 18.278 | 18.328 | 18.508 | 18.373 | 17.779 | 17.796 |
| bta-miR-122 | 31.547 | 31.884 | . | 35.602 | 35.046 | 32.910 | 34.487 |
| bta-miR-940 | 22.625 | 22.451 | 22.229 | 23.118 | 24.247 | 24.804 | 22.980 |
| bta-miR-1224 | 21.221 | 22.576 | 20.169 | 18.720 | 20.722 | 23.324 | 23.375 |
| bta-miR-95 | 30.818 | 30.830 | 29.923 | 30.195 | 31.387 | 33.345 | 30.312 |
| bta-miR-1225-3p | 24.601 | 23.771 | 27.224 | 24.655 | 25.685 | 26.806 | 24.743 |
| bta-miR-96 | 27.736 | 27.241 | 28.650 | 27.318 | 30.154 | 31.476 | 27.027 |
| bta-miR-1246 | 20.816 | 19.132 | 20.279 | 20.331 | 19.307 | 23.812 | 20.201 |
| bta-miR-1247-3p | 26.837 | 27.243 | 31.511 | 27.569 | 28.315 | 29.298 | 27.685 |
| bta-miR-1296 | 25.804 | 27.127 | 31.796 | 28.255 | 28.877 | 29.341 | 28.295 |
| bta-miR-1247-5p | 26.541 | 26.140 | 26.373 | 26.009 | 27.485 | 29.514 | 26.778 |
| bta-miR-1298 | 34.312 | 32.692 | . | . | . | . | 33.790 |
| bta-miR-1248 | 27.816 | 27.754 | 31.924 | 29.682 | 30.746 | 30.850 | 28.364 |
| bta-miR-1301 | 30.970 | 30.834 | 33.821 | 31.022 | 32.810 | 31.665 | 32.672 |
| bta-miR-1249 | 28.376 | 28.319 | 32.250 | 30.825 | 29.473 | 31.015 | 28.817 |
| bta-miR-1306 | 29.289 | 28.774 | 31.973 | 31.508 | 31.546 | 31.615 | 29.581 |
| bta-miR-1260b | 23.578 | 23.471 | 22.445 | 21.985 | 22.438 | 24.994 | 22.817 |
| bta-miR-1307 | 21.068 | 21.722 | 25.863 | 21.540 | 24.078 | 22.657 | 22.508 |
| bta-miR-1271 | 32.901 | 32.972 | 32.151 | 34.483 | 34.682 | . | 32.176 |
| bta-miR-1343-3p | 24.987 | 25.714 | 29.097 | 25.313 | 25.721 | 28.154 | 26.470 |
| bta-miR-1277 | . | . | . | . | . | . | . |
| bta-miR-1343-5p | 20.599 | 19.818 | 21.080 | 18.605 | 21.340 | 23.778 | 20.177 |
| bta-miR-1281 | 25.724 | 26.592 | 30.889 | 27.374 | 27.736 | 28.317 | 27.007 |
| bta-miR-1388-3p | 29.324 | 29.240 | 30.810 | 29.420 | 29.083 | 31.437 | 29.931 |
| bta-miR-1282 | 32.874 | 31.827 | 34.942 | 35.037 | 32.869 | 34.987 | 33.058 |
| bta-miR-1284 | 31.072 | 30.892 | 36.763 | 32.841 | 32.593 | 33.058 | 31.722 |
| bta-miR-1287 | 30.750 | 31.810 | 36.468 | 32.891 | 32.826 | 33.719 | 32.873 |
| bta-miR-1291 | 29.160 | 29.274 | 31.876 | 30.662 | 31.905 | 31.717 | 30.345 |
|  | . | . | . | . | . | . | . |

| **Supplementary Table S4:** List of miRNA identified (Cycle Threshold) in UF-EVs from Low and High NEB dairy cows collected at 60 DPC. | | | | | | | |
| --- | --- | --- | --- | --- | --- | --- | --- |
|  |  |  |  |  |  |  |  |
| **MicroRNAs** | **Group - 60 DPC** | | | | | |  |
|  | **Low NEB** | | | **High NEB** | | |  |
|  | **EVS LU 185** | **EVS LU 212** | **EVS LU 254** | **EVS LU 213** | **EVS LU 171** | **EVS LU 233** |  |
| bta-let-7a-3p | 30.866 | 32.260 | 33.802 | 31.823 | 31.503 | 32.891 |  |
| bta-miR-103 | 26.267 | 25.970 | 28.470 | 26.762 | 27.833 | 28.948 |  |
| bta-let-7a-5p | 23.759 | 24.220 | 26.083 | 24.259 | 23.836 | 26.157 |  |
| bta-miR-105a | 31.899 | 31.859 | 34.253 | 32.180 | 32.853 | 32.411 |  |
| bta-let-7b | 22.128 | 22.206 | 24.259 | 22.325 | 23.240 | 24.722 |  |
| bta-miR-105b | 34.218 | 33.802 | . | 35.538 | 35.203 | 35.013 |  |
| bta-let-7c | 22.452 | 22.788 | 24.547 | 23.396 | 23.189 | 24.834 |  |
| bta-miR-106a | 25.168 | 25.876 | 27.685 | 26.078 | 26.794 | 26.831 |  |
| bta-let-7d | 25.671 | 25.757 | 27.718 | 26.794 | 25.432 | 27.158 |  |
| bta-miR-106b | 26.502 | 27.221 | 28.782 | 27.051 | 27.191 | 27.756 |  |
| bta-let-7e | 23.375 | 23.472 | 25.581 | 23.876 | 23.610 | 25.754 |  |
| bta-miR-107 | 29.882 | 29.608 | 33.663 | 30.614 | 31.952 | 33.226 |  |
| bta-let-7f | 25.595 | 26.175 | 27.643 | 26.252 | 25.818 | 27.514 |  |
| bta-miR-10a | 27.839 | 28.163 | 29.007 | 28.789 | 29.015 | 29.792 |  |
| bta-let-7g | 26.388 | 26.862 | 28.589 | 26.500 | 25.233 | 28.444 |  |
| bta-miR-10b | 28.153 | 28.659 | 30.129 | 28.990 | 29.062 | 30.429 |  |
| bta-let-7i | 27.568 | 26.779 | 28.820 | 28.313 | 28.536 | 29.776 |  |
| bta-miR-122 | 32.486 | 32.345 | 35.005 | 32.305 | 35.429 | 32.864 |  |
| bta-miR-1 | 29.622 | 34.187 | 35.116 | . | 35.143 | . |  |
| bta-miR-124a | 30.694 | 30.685 | 31.849 | 30.310 | 31.138 | 30.888 |  |
| bta-miR-100 | 30.608 | 29.507 | 30.973 | 30.748 | 30.105 | 32.115 |  |
| bta-miR-124b | 30.512 | 30.564 | 32.234 | 30.399 | 30.823 | 31.603 |  |
| bta-miR-101 | 27.749 | 28.881 | 29.806 | 28.392 | 27.330 | 30.450 |  |
| bta-miR-125a | 26.839 | 26.736 | 28.806 | 27.190 | 28.409 | 29.030 |  |
| bta-miR-125b | 25.580 | 25.260 | 27.150 | 25.834 | 26.533 | 27.993 |  |
| bta-miR-133b | 33.645 | 32.726 | 36.866 | 32.842 | 35.043 | 32.830 |  |
| bta-miR-126-3p | 31.040 | 30.582 | 30.759 | 31.698 | 30.747 | 33.903 |  |
| bta-miR-133c | 36.065 | 35.704 | . | 36.019 | . | . |  |
| bta-miR-126-5p | 32.661 | 32.388 | 32.663 | 32.754 | 32.841 | 34.997 |  |
| bta-miR-134 | 29.820 | 28.980 | 31.869 | 29.155 | 31.635 | 30.532 |  |
| bta-miR-127 | 27.552 | 28.484 | 27.348 | 26.099 | 26.237 | 26.038 |  |
| bta-miR-135a | 27.790 | 28.627 | 30.271 | 29.056 | 31.012 | 30.629 |  |
| bta-miR-128 | 29.568 | 29.714 | 31.313 | 30.510 | 31.804 | 31.513 |  |
| bta-miR-135b | 28.960 | 29.799 | 30.835 | 29.821 | 31.722 | 30.888 |  |
| bta-miR-129 | 29.833 | 29.607 | 31.553 | 29.115 | 29.014 | 29.880 |  |
| bta-miR-136 | 34.183 | 32.996 | 34.527 | 34.767 | 34.968 | . |  |
| bta-miR-129-3p | 31.255 | 33.914 | 35.862 | 30.225 | 27.802 | 33.207 |  |
| bta-miR-137 | 36.567 | 35.183 | . | . | . | . |  |
| bta-miR-129-5p | 29.819 | 29.880 | 30.851 | 29.011 | 29.009 | 29.901 |  |
| bta-miR-138 | 28.767 | 28.567 | 31.758 | 29.224 | 30.828 | 30.773 |  |
| bta-miR-130a | 29.764 | 31.393 | 31.829 | 31.512 | 30.719 | 32.030 |  |
| bta-miR-139 | 30.204 | 29.526 | 31.941 | 30.187 | 30.872 | 31.599 |  |
| bta-miR-130b | 28.897 | 27.770 | 30.605 | 29.711 | 29.384 | 31.882 |  |
| bta-miR-140 | 28.678 | 29.425 | 31.630 | 30.015 | 29.936 | 32.096 |  |
| bta-miR-132 | 31.788 | 31.341 | 33.017 | 31.429 | 31.967 | 31.970 |  |
| bta-miR-141 | 25.280 | 25.918 | 27.535 | 25.666 | 26.261 | 27.382 |  |
| bta-miR-133a | 28.419 | 27.980 | 30.833 | 27.950 | 30.212 | 30.002 |  |
| bta-miR-142-3p | 33.559 | 33.758 | 34.991 | 33.548 | 34.000 | . |  |
| bta-miR-142-5p | 31.780 | 32.117 | 31.187 | 31.918 | 32.355 | 32.671 |  |
| bta-miR-151-3p | 28.789 | 28.123 | 30.707 | 28.954 | 30.131 | 30.473 |  |
| bta-miR-143 | 29.990 | 27.119 | 31.338 | 30.503 | 30.787 | 32.409 |  |
| bta-miR-151-5p | 28.434 | 28.750 | 29.856 | 28.685 | 28.619 | 30.385 |  |
| bta-miR-144 | . | . | . | 32.360 | . | . |  |
| bta-miR-152 | 32.524 | 32.676 | 33.685 | 32.806 | 33.966 | 32.949 |  |
| bta-miR-145 | 27.468 | 26.625 | 28.648 | 28.636 | 28.327 | 30.420 |  |
| bta-miR-153 | 31.908 | 32.752 | . | 35.162 | 33.175 | . |  |
| bta-miR-146a | 33.934 | 36.338 | 33.982 | 35.754 | 33.980 | . |  |
| bta-miR-154a | 34.566 | 33.401 | 35.283 | 34.204 | 34.928 | 36.812 |  |
| bta-miR-146b | 32.945 | 35.165 | 34.316 | 34.439 | . | . |  |
| bta-miR-154b | 33.909 | 32.203 | 34.668 | 34.883 | 34.907 | 35.432 |  |
| bta-miR-147 | 30.284 | 29.546 | 31.417 | 30.026 | 31.693 | 32.404 |  |
| bta-miR-154c | 35.372 | 33.835 | 33.888 | 33.473 | 36.340 | 35.749 |  |
| bta-miR-148a | 26.614 | 26.810 | 28.743 | 26.879 | 26.102 | 28.829 |  |
| bta-miR-155 | 27.487 | 26.624 | 28.321 | 27.666 | 29.819 | 30.781 |  |
| bta-miR-148b | 27.201 | 26.765 | 28.757 | 27.077 | 26.497 | 28.935 |  |
| bta-miR-15a | 26.834 | 27.035 | 28.920 | 27.766 | 27.781 | 29.849 |  |
| bta-miR-149-3p | 26.364 | 24.224 | 26.040 | 24.150 | 24.352 | 25.199 |  |
| bta-miR-15b | 28.065 | 27.752 | 29.838 | 29.170 | 30.231 | 30.690 |  |
| bta-miR-149-5p | 26.867 | 26.782 | 28.346 | 27.032 | 27.256 | 27.251 |  |
| bta-miR-16a | 26.286 | 26.346 | 27.990 | 26.906 | 26.878 | 29.072 |  |
| bta-miR-150 | 32.270 | 30.257 | 33.980 | 31.886 | 33.660 | 35.027 |  |
| bta-miR-16b | 26.987 | 27.133 | 28.799 | 27.998 | 28.364 | 29.842 |  |
| bta-miR-17-3p | 30.236 | 29.524 | 31.926 | 30.734 | 31.899 | 31.842 |  |
| bta-miR-188 | 31.315 | 31.705 | 31.585 | 30.424 | 30.189 | 30.712 |  |
| bta-miR-17-5p | 28.905 | 29.630 | 31.685 | 29.681 | 30.718 | 30.959 |  |
| bta-miR-18a | 28.738 | 28.864 | 30.554 | 29.241 | 29.674 | 29.903 |  |
| bta-miR-181a | 27.929 | 28.741 | 30.294 | 28.758 | 30.192 | 30.455 |  |
| bta-miR-18b | 30.905 | 30.819 | 32.742 | 31.800 | 32.692 | 32.882 |  |
| bta-miR-181b | 27.791 | 28.145 | 29.684 | 28.744 | 30.660 | 29.826 |  |
| bta-miR-190a | 31.658 | 31.150 | 34.598 | 31.343 | 31.575 | 34.350 |  |
| bta-miR-181c | 29.236 | 29.747 | 31.850 | 30.443 | 31.730 | 31.018 |  |
| bta-miR-190b | 30.382 | 30.711 | 30.773 | 30.756 | 31.036 | 31.198 |  |
| bta-miR-181d | 27.831 | 28.365 | 29.785 | 28.618 | 31.625 | 30.056 |  |
| bta-miR-191 | 27.627 | 27.751 | 29.459 | 27.764 | 28.242 | 29.336 |  |
| bta-miR-182 | 27.518 | 27.808 | 30.664 | 28.897 | 29.783 | 29.464 |  |
| bta-miR-192 | 31.678 | 31.815 | 32.829 | 31.165 | 32.394 | 32.036 |  |
| bta-miR-183 | 29.665 | 29.077 | 31.744 | 31.039 | 32.740 | 31.905 |  |
| bta-miR-193a | 33.769 | 33.932 | 35.463 | 33.353 | 35.724 | 34.389 |  |
| bta-miR-184 | 34.576 | 31.560 | 30.278 | 34.228 | 36.017 | 34.941 |  |
| bta-miR-193a-3p | 29.534 | 29.177 | 30.574 | 29.829 | 30.315 | 32.769 |  |
| bta-miR-185 | 28.922 | 28.815 | 30.809 | 29.495 | 31.178 | 30.800 |  |
| bta-miR-193a-5p | 29.722 | 28.766 | 30.271 | 28.969 | 30.512 | 30.019 |  |
| bta-miR-186 | 28.140 | 29.456 | 30.730 | 28.839 | 28.543 | 29.827 |  |
| bta-miR-193b | 31.855 | 31.848 | 33.917 | 31.346 | 31.885 | 31.871 |  |
| bta-miR-187 | 29.874 | 29.796 | 30.772 | 28.696 | 29.807 | 28.559 |  |
| bta-miR-194 | 29.976 | 30.269 | 31.830 | 30.698 | 30.889 | 31.904 |  |
| bta-miR-195 | 28.972 | 28.672 | 31.723 | 30.088 | 30.017 | 32.705 |  |
| bta-miR-200c | 24.741 | 25.118 | 26.536 | 24.533 | 24.791 | 26.280 |  |
| bta-miR-196a | 29.813 | 30.025 | 31.127 | 30.241 | 31.874 | 32.921 |  |
| bta-miR-202 | 32.801 | 33.921 | . | . | 33.278 | . |  |
| bta-miR-196b | 29.788 | 29.456 | 31.326 | 30.801 | 31.616 | 32.632 |  |
| bta-miR-204 | 29.278 | 28.820 | 31.550 | 29.595 | 29.186 | 30.250 |  |
| bta-miR-197 | 27.815 | 27.747 | 28.757 | 26.959 | 28.777 | 28.301 |  |
| bta-miR-205 | 28.471 | 28.449 | 29.140 | 28.614 | 29.924 | 30.150 |  |
| bta-miR-199a-3p | 28.575 | 27.276 | 28.301 | 29.176 | 28.691 | 31.948 |  |
| bta-miR-206 | 31.839 | 32.185 | 32.713 | 30.775 | 31.855 | 31.425 |  |
| bta-miR-199a-5p | 28.723 | 28.445 | 28.850 | 29.552 | 28.805 | 31.504 |  |
| bta-miR-208a | . | . | 35.125 | 36.294 | 34.791 | 35.387 |  |
| bta-miR-199b | 30.148 | 29.396 | 29.612 | 30.045 | 29.595 | 32.894 |  |
| bta-miR-208b | 36.448 | 33.944 | 36.998 | 35.013 | . | . |  |
| bta-miR-199c | 27.800 | 26.458 | 27.859 | 28.827 | 27.990 | 31.186 |  |
| bta-miR-20a | 25.863 | 26.750 | 27.939 | 26.406 | 27.740 | 27.780 |  |
| bta-miR-19a | 24.485 | 25.520 | 27.199 | 25.338 | 26.211 | 27.516 |  |
| bta-miR-20b | 26.236 | 27.155 | 28.520 | 26.790 | 28.260 | 28.438 |  |
| bta-miR-19b | 24.470 | 25.520 | 26.970 | 25.293 | 25.938 | 27.705 |  |
| bta-miR-21-3p | 30.732 | 30.691 | 31.188 | 29.575 | 31.700 | 30.354 |  |
| bta-miR-200a | 24.934 | 26.324 | 27.040 | 25.094 | 24.885 | 26.667 |  |
| bta-miR-21-5p | 29.839 | 30.664 | 31.530 | 30.406 | 30.578 | 31.565 |  |
| bta-miR-200b | 23.208 | 23.957 | 25.160 | 23.304 | 22.773 | 25.099 |  |
| bta-miR-210 | 26.749 | 26.393 | 26.095 | 26.268 | 27.420 | 27.492 |  |
| bta-miR-211 | 28.214 | 28.379 | 29.058 | 28.109 | 27.754 | 26.997 |  |
| bta-miR-22-5p | 31.219 | 31.127 | 32.679 | 31.590 | 31.858 | 34.454 |  |
| bta-miR-212 | 34.812 | 35.245 | . | 34.164 | 35.912 | 34.945 |  |
| bta-miR-221 | 29.267 | 28.613 | 29.793 | 28.253 | 26.679 | 30.779 |  |
| bta-miR-214 | 24.518 | 24.711 | 24.115 | 23.557 | 25.054 | 24.765 |  |
| bta-miR-222 | 26.613 | 26.037 | 28.458 | 26.967 | 26.786 | 29.515 |  |
| bta-miR-215 | 32.879 | 32.216 | 33.646 | 34.098 | 35.220 | 33.926 |  |
| bta-miR-223 | 30.480 | 28.701 | 31.483 | 28.101 | 30.806 | 31.582 |  |
| bta-miR-216a | 30.602 | 31.252 | 33.893 | 30.812 | 31.709 | 31.902 |  |
| bta-miR-224 | 28.394 | 27.932 | 29.745 | 29.370 | 30.700 | 30.064 |  |
| bta-miR-216b | 33.061 | 34.426 | 36.371 | 34.259 | . | 34.219 |  |
| bta-miR-23a | 24.477 | 24.816 | 26.256 | 24.515 | 23.781 | 26.612 |  |
| bta-miR-217 | 33.210 | 31.846 | 31.811 | 31.829 | 28.905 | 32.639 |  |
| bta-miR-23b-3p | 25.841 | 25.814 | 27.774 | 26.485 | 26.121 | 27.210 |  |
| bta-miR-218 | 31.784 | 30.535 | 32.923 | 32.998 | 32.660 | 33.355 |  |
| bta-miR-23b-5p | 31.515 | 31.890 | 34.977 | 32.956 | 33.743 | 33.447 |  |
| bta-miR-219 | 29.774 | 29.484 | 31.693 | 29.132 | 30.057 | 29.764 |  |
| bta-miR-24 | 32.707 | 33.864 | . | 34.649 | 34.190 | 34.997 |  |
| bta-miR-219-3p | 30.375 | 30.614 | 31.890 | 29.766 | 31.338 | 31.046 |  |
| bta-miR-24-3p | 24.614 | 24.677 | 26.685 | 24.614 | 25.369 | 27.492 |  |
| bta-miR-219-5p | 34.704 | 34.368 | 36.082 | 35.045 | 35.037 | . |  |
| bta-miR-25 | 25.728 | 25.752 | 27.725 | 26.441 | 27.764 | 27.832 |  |
| bta-miR-22-3p | 3.932 | 4.128 | . | 3.487 | 4.476 | . |  |
| bta-miR-26a | 24.422 | 24.905 | 26.426 | 24.766 | 24.661 | 26.694 |  |
| bta-miR-26b | 26.650 | 27.250 | 27.668 | 26.924 | 26.390 | 28.444 |  |
| bta-miR-29d-3p | 28.259 | 28.678 | 28.966 | 28.097 | 27.123 | 29.376 |  |
| bta-miR-26c | . | 35.281 | 36.524 | 35.768 | . | 35.203 |  |
| bta-miR-29d-5p | 30.769 | 31.448 | 32.795 | 31.551 | 30.844 | 33.153 |  |
| bta-miR-27a-3p | 24.993 | 25.801 | 26.923 | 25.135 | 25.009 | 27.087 |  |
| bta-miR-29e | 29.552 | 29.824 | 30.752 | 29.715 | 28.804 | 31.836 |  |
| bta-miR-27a-5p | 31.738 | 31.831 | 33.634 | 29.798 | 29.602 | 31.224 |  |
| bta-miR-301a | 32.318 | 32.269 | 32.940 | 32.284 | 34.200 | 33.907 |  |
| bta-miR-27b | 25.629 | 26.586 | 27.929 | 25.916 | 25.065 | 27.922 |  |
| bta-miR-301b | 32.681 | 33.352 | 34.210 | 31.845 | 33.890 | 35.037 |  |
| bta-miR-28 | 30.493 | 30.672 | 32.854 | 31.655 | 31.533 | 32.938 |  |
| bta-miR-302a | 36.367 | 34.163 | 34.049 | 34.032 | 32.859 | . |  |
| bta-miR-296-3p | 28.586 | 28.346 | 26.571 | 27.815 | 24.655 | 28.619 |  |
| bta-miR-302b | 29.833 | 36.826 | . | 32.182 | 32.114 | . |  |
| bta-miR-296-5p | 29.824 | 29.711 | 31.258 | 29.987 | 31.613 | 30.579 |  |
| bta-miR-302c | 32.903 | 33.950 | 33.929 | 34.110 | 35.053 | 34.361 |  |
| bta-miR-299 | 35.492 | 35.169 | . | 34.804 | 33.653 | . |  |
| bta-miR-302d | . | 34.471 | . | 34.543 | 32.776 | . |  |
| bta-miR-29a | 25.001 | 24.885 | 26.854 | 25.241 | 24.333 | 27.704 |  |
| bta-miR-3064 | 31.808 | 31.817 | 33.257 | 32.920 | . | 32.874 |  |
| bta-miR-29b | 26.465 | 26.732 | 27.764 | 26.368 | 25.276 | 28.672 |  |
| bta-miR-30a-5p | 27.171 | 27.495 | 29.460 | 27.929 | 27.895 | 28.844 |  |
| bta-miR-29c | 24.830 | 25.264 | 26.693 | 24.988 | 23.937 | 27.327 |  |
| bta-miR-30b-3p | 32.912 | 34.632 | 35.781 | 32.720 | 34.943 | 34.929 |  |
| bta-miR-30b-5p | 27.731 | 28.725 | 29.538 | 28.132 | 27.661 | 29.489 |  |
| bta-miR-328 | 29.519 | 29.224 | 30.115 | 29.087 | 29.938 | 30.102 |  |
| bta-miR-30c | 26.793 | 27.594 | 29.472 | 27.696 | 27.609 | 29.160 |  |
| bta-miR-329a | . | 36.632 | . | . | . | . |  |
| bta-miR-30d | 27.260 | 27.648 | 29.726 | 27.841 | 28.606 | 29.283 |  |
| bta-miR-329b | 33.922 | 33.506 | . | 35.285 | . | 34.267 |  |
| bta-miR-30e-5p | 27.080 | 27.591 | 29.734 | 27.910 | 28.641 | 29.711 |  |
| bta-miR-330 | 29.734 | 29.654 | 30.755 | 29.414 | 30.793 | 30.429 |  |
| bta-miR-30f | 27.447 | 27.869 | 29.742 | 28.394 | 28.753 | 29.715 |  |
| bta-miR-331-3p | 28.086 | 28.430 | 31.023 | 28.782 | 30.184 | 30.436 |  |
| bta-miR-31 | 24.752 | 25.155 | 27.503 | 24.929 | 25.763 | 27.469 |  |
| bta-miR-331-5p | 30.359 | 31.095 | 31.268 | 30.547 | 31.399 | 30.824 |  |
| bta-miR-32 | 31.186 | 31.412 | 32.423 | 31.395 | 31.836 | 32.701 |  |
| bta-miR-335 | 25.915 | 26.829 | 26.411 | 26.833 | 25.878 | 26.411 |  |
| bta-miR-320a | 25.165 | 25.494 | 26.602 | 25.389 | 27.304 | 26.524 |  |
| bta-miR-338 | 34.948 | 34.758 | 35.445 | 34.535 | 34.915 | 34.366 |  |
| bta-miR-320b | 28.489 | 29.326 | 29.763 | 28.283 | 28.608 | 28.807 |  |
| bta-miR-339a | 27.071 | 27.650 | 29.360 | 27.328 | 27.886 | 29.072 |  |
| bta-miR-323 | 31.611 | 31.864 | 32.820 | 30.744 | 32.787 | 31.727 |  |
| bta-miR-339b | 25.271 | 25.991 | 27.746 | 25.788 | 26.271 | 27.693 |  |
| bta-miR-324 | 29.157 | 29.367 | 31.093 | 29.471 | 29.858 | 31.362 |  |
| bta-miR-33a | 28.088 | 29.721 | 29.731 | 28.537 | 28.302 | 29.823 |  |
| bta-miR-326 | 28.035 | 28.056 | 29.536 | 27.772 | 29.779 | 28.844 |  |
| bta-miR-33b | 29.523 | 30.321 | 30.918 | 29.828 | 29.573 | 30.805 |  |
| bta-miR-340 | 35.827 | 32.785 | 36.015 | 35.491 | . | . |  |
| bta-miR-365-3p | 29.346 | 28.825 | 30.627 | 28.951 | 29.680 | 31.387 |  |
| bta-miR-342 | 28.499 | 28.405 | 29.603 | 28.784 | 29.416 | 29.960 |  |
| bta-miR-365-5p | 31.057 | 30.448 | 32.544 | 28.351 | 30.017 | 30.282 |  |
| bta-miR-345-3p | 29.638 | 31.137 | 31.837 | 29.663 | 29.597 | 30.466 |  |
| bta-miR-367 | 35.063 | . | . | 36.977 | . | . |  |
| bta-miR-345-5p | 27.069 | 27.249 | 28.757 | 27.530 | 26.735 | 27.762 |  |
| bta-miR-369-3p | 35.365 | 35.610 | 36.060 | . | . | . |  |
| bta-miR-346 | 26.708 | 25.420 | 28.789 | 25.974 | 27.409 | 27.409 |  |
| bta-miR-369-5p | 35.170 | . | . | . | . | . |  |
| bta-miR-34a | 26.242 | 25.798 | 28.205 | 26.527 | 27.136 | 28.868 |  |
| bta-miR-370 | 29.824 | 29.110 | 31.476 | 28.936 | 30.715 | 29.759 |  |
| bta-miR-34b | 26.793 | 25.756 | 27.910 | 26.833 | 27.130 | 28.427 |  |
| bta-miR-371 | 32.120 | 32.347 | 31.503 | 32.723 | 32.259 | 33.178 |  |
| bta-miR-34c | 26.800 | 25.674 | 27.837 | 26.635 | 26.968 | 27.924 |  |
| bta-miR-374a | 27.762 | 28.634 | 29.728 | 28.692 | 27.791 | 29.973 |  |
| bta-miR-361 | 26.651 | 26.768 | 28.676 | 26.070 | 26.457 | 28.422 |  |
| bta-miR-374b | 28.996 | 29.106 | 30.544 | 29.534 | 29.836 | 31.816 |  |
| bta-miR-362-3p | 32.163 | 32.904 | 34.011 | 33.528 | 31.800 | 35.227 |  |
| bta-miR-375 | 26.558 | 24.777 | 26.489 | 26.664 | 26.427 | 27.234 |  |
| bta-miR-362-5p | 31.527 | 31.422 | 32.602 | 31.168 | 29.731 | 31.078 |  |
| bta-miR-376a | 33.091 | 36.504 | 36.139 | 35.031 | 35.854 | 34.984 |  |
| bta-miR-363 | 33.940 | 34.049 | 33.648 | 34.550 | 33.753 | 35.325 |  |
| bta-miR-376b | 35.005 | 33.654 | 34.146 | 34.835 | 34.454 | . |  |
| bta-miR-376c | 35.993 | 33.874 | 35.050 | 35.867 | . | . |  |
| bta-miR-382 | 33.381 | 32.998 | 34.247 | 33.417 | 34.933 | 33.944 |  |
| bta-miR-376d | 33.393 | 33.929 | 35.413 | 34.213 | 33.959 | . |  |
| bta-miR-383 | 31.410 | 31.362 | 33.976 | 29.829 | 31.643 | 31.522 |  |
| bta-miR-376e | 35.011 | 33.184 | 34.006 | 35.626 | 34.086 | . |  |
| bta-miR-409a | 34.628 | 34.686 | . | 36.798 | . | 36.104 |  |
| bta-miR-377 | 31.827 | 32.264 | 33.014 | 32.846 | 32.899 | 32.263 |  |
| bta-miR-409b | 33.930 | 34.960 | 35.460 | 34.200 | 36.028 | . |  |
| bta-miR-378 | 27.759 | 27.031 | 29.774 | 27.653 | 27.959 | 28.921 |  |
| bta-miR-410 | 31.416 | 31.797 | 31.802 | 30.508 | 32.163 | 31.053 |  |
| bta-miR-378b | 27.748 | 27.185 | 29.596 | 27.775 | 28.405 | 29.042 |  |
| bta-miR-411a | 32.811 | 33.030 | 33.214 | 33.360 | 32.522 | 33.992 |  |
| bta-miR-378c | 32.802 | 32.390 | 35.205 | 33.134 | 33.783 | 34.431 |  |
| bta-miR-411b | 33.258 | 33.652 | 35.032 | 33.640 | . | . |  |
| bta-miR-378d | 31.392 | 30.722 | 32.918 | 30.331 | 31.466 | 31.765 |  |
| bta-miR-411c-3p | 33.213 | 33.477 | 34.262 | 33.094 | 35.214 | 33.986 |  |
| bta-miR-379 | 32.125 | 31.719 | 32.368 | 31.851 | 33.527 | 35.938 |  |
| bta-miR-411c-5p | 34.730 | 34.923 | . | 35.324 | . | 36.192 |  |
| bta-miR-380-3p | 32.027 | 31.822 | 33.998 | 32.498 | 32.755 | 31.993 |  |
| bta-miR-412 | 32.461 | 32.615 | 33.973 | 33.093 | 33.547 | 34.711 |  |
| bta-miR-380-5p | 32.785 | 34.997 | 34.394 | 32.906 | 33.625 | 33.073 |  |
| bta-miR-421 | 26.544 | 28.224 | 25.429 | 27.128 | 25.447 | 25.099 |  |
| bta-miR-381 | 31.109 | 30.999 | 34.439 | 30.971 | 32.390 | 32.009 |  |
| bta-miR-423-3p | 26.813 | 26.654 | 28.768 | 26.746 | 28.815 | 28.584 |  |
| bta-miR-423-5p | 26.515 | 26.013 | 28.182 | 26.724 | 27.018 | 28.175 |  |
| bta-miR-449c | 28.441 | 28.182 | 29.966 | 27.953 | 31.677 | 31.504 |  |
| bta-miR-424-3p | 31.840 | 31.902 | 32.137 | 30.724 | 30.732 | 32.827 |  |
| bta-miR-449d | 28.467 | 28.026 | 30.399 | 27.400 | 28.729 | 28.378 |  |
| bta-miR-424-5p | 28.164 | 29.659 | 30.628 | 27.091 | 25.555 | 30.794 |  |
| bta-miR-450a | 29.347 | 30.831 | 32.742 | 29.345 | 28.304 | 31.623 |  |
| bta-miR-425-3p | 23.700 | 24.216 | 23.711 | 21.253 | 21.085 | 20.545 |  |
| bta-miR-450b | 30.497 | 31.284 | 32.638 | 31.659 | 30.718 | 32.461 |  |
| bta-miR-425-5p | 28.812 | 28.774 | 30.577 | 29.136 | 28.750 | 30.542 |  |
| bta-miR-451 | 29.802 | 28.362 | 27.284 | 30.155 | 28.363 | 32.679 |  |
| bta-miR-429 | 27.406 | 28.131 | 29.401 | 27.327 | 26.997 | 29.436 |  |
| bta-miR-452 | 30.837 | 31.913 | 33.554 | 31.602 | 32.392 | 31.806 |  |
| bta-miR-431 | 28.668 | 28.819 | 30.759 | 28.037 | 29.774 | 29.418 |  |
| bta-miR-4523 | 30.314 | 30.293 | 31.713 | 29.312 | 30.153 | 29.781 |  |
| bta-miR-432 | 30.753 | 31.022 | 32.831 | 30.594 | 30.889 | 31.607 |  |
| bta-miR-453 | 31.817 | 31.009 | 31.789 | 31.086 | 31.041 | 31.158 |  |
| bta-miR-433 | 31.474 | 30.072 | 32.778 | 29.786 | 31.609 | 30.821 |  |
| bta-miR-454 | 31.708 | 32.025 | 33.490 | 33.208 | 33.928 | 33.583 |  |
| bta-miR-448 | 34.121 | 34.838 | . | 34.985 | 35.450 | 35.010 |  |
| bta-miR-455-3p | 33.737 | 32.450 | 35.904 | 35.573 | 34.025 | 35.828 |  |
| bta-miR-449a | 25.628 | 26.213 | 27.165 | 25.401 | 28.761 | 30.073 |  |
| bta-miR-455-5p | 30.777 | 30.232 | 32.234 | 30.812 | 31.104 | 32.843 |  |
| bta-miR-449b | 27.791 | 27.738 | 28.948 | 27.325 | 30.779 | 30.782 |  |
| bta-miR-483 | 31.706 | 31.827 | 32.892 | 31.363 | 32.878 | 32.001 |  |
| bta-miR-484 | 27.899 | 27.705 | 28.777 | 27.895 | 28.995 | 29.265 |  |
| bta-miR-496 | 32.701 | 32.644 | 34.602 | 32.690 | 34.166 | 34.907 |  |
| bta-miR-485 | 32.604 | 32.918 | 33.285 | 32.879 | 34.016 | 33.028 |  |
| bta-miR-497 | 28.951 | 28.225 | 31.141 | 29.779 | 30.800 | 32.800 |  |
| bta-miR-486 | . | 26.449 | 23.707 | 24.522 | 22.120 | 23.315 |  |
| bta-miR-499 | 31.556 | 31.869 | 34.933 | 29.437 | 27.444 | 33.725 |  |
| bta-miR-487a | 32.911 | 32.949 | 35.842 | 33.004 | 36.110 | 33.102 |  |
| bta-miR-500 | 29.058 | 29.358 | 29.842 | 28.951 | 29.541 | 29.706 |  |
| bta-miR-487b | 33.894 | 32.932 | 36.207 | 32.969 | 33.988 | 34.194 |  |
| bta-miR-502a | 28.734 | 29.820 | 29.462 | 29.410 | 29.556 | 29.172 |  |
| bta-miR-488 | 32.314 | 31.864 | 34.507 | 31.807 | 33.701 | 34.151 |  |
| bta-miR-502b | 32.702 | 32.020 | 33.983 | 32.053 | 31.802 | 33.473 |  |
| bta-miR-489 | 33.955 | 34.950 | 35.002 | 33.572 | 32.750 | 33.888 |  |
| bta-miR-503-3p | 28.131 | 28.006 | 28.954 | 27.274 | 28.362 | 27.309 |  |
| bta-miR-490 | 29.765 | 29.732 | 32.263 | 29.611 | 31.357 | 30.575 |  |
| bta-miR-503-5p | 30.640 | 31.622 | 32.636 | 31.527 | 31.238 | 32.182 |  |
| bta-miR-491 | 29.894 | 29.849 | 31.854 | 30.435 | 30.726 | 32.509 |  |
| bta-miR-504 | 31.012 | 30.862 | 33.878 | 30.518 | 31.495 | 32.425 |  |
| bta-miR-493 | 27.513 | 27.728 | 27.794 | 26.091 | 25.625 | 25.364 |  |
| bta-miR-505 | 27.366 | 27.538 | 28.485 | 26.786 | 27.392 | 27.741 |  |
| bta-miR-494 | 14.555 | 15.119 | 16.228 | 14.299 | 12.792 | 17.329 |  |
| bta-miR-532 | 32.244 | 32.850 | 32.847 | 31.768 | 30.918 | 31.836 |  |
| bta-miR-495 | 32.995 | 33.436 | 33.922 | 34.912 | 33.146 | 35.294 |  |
| bta-miR-539 | 34.305 | 35.469 | 36.475 | 35.410 | 36.844 | 34.547 |  |
| bta-miR-541 | 29.803 | 29.799 | 30.742 | 28.728 | 29.810 | 29.848 |  |
| bta-miR-582 | 34.269 | 31.865 | 33.743 | . | 33.580 | . |  |
| bta-miR-542-5p | 32.083 | 32.861 | 33.714 | 30.862 | 31.826 | 32.757 |  |
| bta-miR-584 | 27.812 | 27.737 | 27.658 | 26.351 | 26.602 | 27.002 |  |
| bta-miR-543 | 31.816 | 32.131 | 32.477 | 31.845 | 35.872 | 33.721 |  |
| bta-miR-592 | 30.301 | 30.860 | 32.678 | 30.961 | 32.602 | 33.989 |  |
| bta-miR-544a | 34.754 | 34.499 | . | 34.554 | . | . |  |
| bta-miR-599 | 35.899 | . | . | . | . | 35.336 |  |
| bta-miR-544b | 35.521 | 33.907 | 35.334 | 35.146 | . | . |  |
| bta-miR-615 | 6.552 | 6.562 | 6.419 | 6.550 | 6.641 | 6.621 |  |
| bta-miR-545-3p | 32.348 | 32.996 | 34.726 | 35.691 | 34.115 | . |  |
| bta-miR-628 | 34.248 | 36.037 | . | 35.855 | 35.366 | 44.443 |  |
| bta-miR-545-5p | 33.191 | 33.169 | 33.943 | 34.879 | 33.045 | 34.545 |  |
| bta-miR-631 | 27.752 | 27.689 | 27.893 | 26.469 | 26.958 | 28.014 |  |
| bta-miR-551a | 33.924 | 35.572 | . | 33.299 | 36.292 | 35.045 |  |
| bta-miR-652 | 28.832 | 28.838 | 30.364 | 28.791 | 29.462 | 31.616 |  |
| bta-miR-551b | 31.431 | 33.993 | . | 34.565 | 34.131 | 36.117 |  |
| bta-miR-653 | . | 36.403 | . | . | . | . |  |
| bta-miR-562 | 33.352 | 34.573 | 34.399 | 33.485 | 34.362 | 35.772 |  |
| bta-miR-654 | 20.838 | 21.053 | 21.210 | 20.786 | 21.115 | 20.962 |  |
| bta-miR-568 | 32.386 | 32.322 | 32.365 | 32.230 | 33.449 | 34.143 |  |
| bta-miR-655 | . | 36.790 | . | . | . | . |  |
| bta-miR-574 | 24.330 | 24.058 | 26.346 | 23.857 | 24.864 | 25.294 |  |
| bta-miR-656 | 35.783 | 34.434 | . | . | . | . |  |
| bta-miR-658 | 31.461 | 30.981 | 30.382 | 29.416 | 27.960 | 29.707 |  |
| bta-miR-758 | 32.110 | 33.593 | 33.274 | 32.058 | 33.755 | 33.005 |  |
| bta-miR-660 | 30.823 | 30.827 | 32.910 | 30.894 | 30.600 | 32.804 |  |
| bta-miR-759 | 31.845 | . | . | . | . | . |  |
| bta-miR-664a | 28.353 | 28.024 | 30.521 | 27.758 | 27.706 | 29.454 |  |
| bta-miR-760-3p | 29.025 | 28.595 | 30.826 | 28.603 | 29.887 | 29.735 |  |
| bta-miR-664b | 25.282 | 25.535 | 25.531 | 25.068 | 24.977 | 24.839 |  |
| bta-miR-760-5p | 28.242 | 27.768 | 28.830 | 26.735 | 27.905 | 28.427 |  |
| bta-miR-665 | 26.900 | 26.345 | 26.213 | 25.730 | 23.634 | 25.403 |  |
| bta-miR-761 | 31.846 | 31.183 | 33.941 | 32.068 | 33.975 | 32.922 |  |
| bta-miR-669 | 26.802 | 26.260 | 29.144 | 26.146 | 27.354 | 27.865 |  |
| bta-miR-763 | 28.896 | 28.786 | 30.656 | 28.758 | 30.730 | 30.034 |  |
| bta-miR-670 | 36.780 | 35.636 | . | 36.299 | 35.024 | 34.593 |  |
| bta-miR-764 | 32.167 | 31.092 | 31.864 | 31.498 | 31.525 | 31.775 |  |
| bta-miR-671 | 29.832 | 29.335 | 30.714 | 28.936 | 28.815 | 29.829 |  |
| bta-miR-767 | 32.306 | 32.226 | 34.463 | 31.482 | 34.928 | 34.311 |  |
| bta-miR-677 | 28.795 | 28.355 | 30.781 | 28.826 | 29.875 | 30.464 |  |
| bta-miR-769 | 31.539 | 30.925 | 32.681 | 30.807 | 31.872 | 31.871 |  |
| bta-miR-7 | 30.745 | 30.619 | 32.339 | 31.478 | 32.166 | 32.641 |  |
| bta-miR-873 | 31.866 | 31.757 | 34.695 | 31.793 | 33.644 | 32.334 |  |
| bta-miR-708 | 32.716 | 31.809 | . | 32.513 | 32.971 | 34.991 |  |
| bta-miR-874 | 28.167 | 27.728 | 30.034 | 25.911 | 26.782 | 28.070 |  |
| bta-miR-744 | 29.352 | 29.058 | 31.812 | 28.820 | 31.863 | 31.772 |  |
| bta-miR-875 | 33.897 | 34.042 | 35.968 | 33.109 | 34.933 | 35.309 |  |
| bta-miR-876 | 34.119 | 34.558 | 36.468 | 33.957 | . | . |  |
| bta-miR-98 | 29.931 | 30.573 | 31.948 | 30.561 | 30.050 | 31.859 |  |
| bta-miR-877 | 28.097 | 27.749 | 28.786 | 27.274 | 27.766 | 26.926 |  |
| bta-miR-99a-3p | 34.129 | 33.796 | 35.395 | 34.734 | 33.929 | 34.926 |  |
| bta-miR-885 | 30.532 | 29.658 | 30.864 | 29.434 | 30.668 | 29.724 |  |
| bta-miR-99a-5p | 30.729 | 28.846 | 30.785 | 31.377 | 30.204 | 34.956 |  |
| bta-miR-9-3p | . | 34.372 | . | . | 34.585 | . |  |
| bta-miR-9-5p | 27.777 | 31.240 | 34.005 | 29.467 | 29.170 | 30.505 |  |
| bta-miR-1179 | 36.736 | 35.686 | . | 35.968 | . | 36.564 |  |
| bta-miR-92a | 24.325 | 24.093 | 26.146 | 25.154 | 26.832 | 26.373 |  |
| bta-miR-1185 | . | . | 35.627 | 35.955 | . | . |  |
| bta-miR-92b | 25.135 | 23.987 | 23.766 | 23.877 | 23.594 | 25.751 |  |
| bta-miR-1193 | 32.303 | 31.956 | 30.916 | 30.407 | 30.812 | 30.837 |  |
| bta-miR-93 | 26.107 | 26.283 | 28.262 | 26.492 | 28.136 | 28.474 |  |
| bta-miR-1197 | 31.439 | . | . | . | 32.479 | . |  |
| bta-miR-935 | 17.724 | 18.030 | 18.384 | 18.340 | 18.522 | 19.213 |  |
| bta-miR-122 | 32.066 | 32.207 | 34.166 | 31.830 | 35.102 | 34.723 |  |
| bta-miR-940 | 23.366 | 22.008 | 23.796 | 23.279 | 21.814 | 24.166 |  |
| bta-miR-1224 | 22.777 | 22.531 | 20.957 | 20.179 | 19.103 | 19.809 |  |
| bta-miR-95 | 30.175 | 31.238 | 32.862 | 30.779 | 30.194 | 32.189 |  |
| bta-miR-1225-3p | 25.694 | 24.373 | 24.384 | 24.713 | 24.292 | 26.157 |  |
| bta-miR-96 | 27.377 | 27.627 | 29.460 | 28.476 | 29.118 | 30.200 |  |
| bta-miR-1246 | 22.799 | 20.714 | 23.109 | 21.509 | 19.543 | 23.811 |  |
| bta-miR-1247-3p | 27.793 | 27.452 | 29.009 | 26.992 | 27.543 | 27.740 |  |
| bta-miR-1296 | 27.706 | 27.316 | 29.839 | 26.988 | 29.704 | 28.714 |  |
| bta-miR-1247-5p | 27.778 | 26.994 | 27.204 | 26.761 | 25.705 | 26.099 |  |
| bta-miR-1298 | 33.411 | 32.917 | 36.761 | 33.582 | . | 36.240 |  |
| bta-miR-1248 | 28.758 | 27.987 | 30.769 | 28.749 | 29.525 | 29.805 |  |
| bta-miR-1301 | 31.840 | 31.198 | 34.008 | 30.650 | 31.955 | 32.937 |  |
| bta-miR-1249 | 28.984 | 28.467 | 30.725 | 28.684 | 31.805 | 30.291 |  |
| bta-miR-1306 | 29.775 | 29.070 | 31.609 | 30.272 | 32.500 | 31.622 |  |
| bta-miR-1260b | 23.899 | 22.795 | 23.823 | 23.745 | 22.840 | 23.582 |  |
| bta-miR-1307 | 21.773 | 21.429 | 23.658 | 20.676 | 19.831 | 22.366 |  |
| bta-miR-1271 | 33.072 | 33.644 | . | 33.250 | 33.745 | 36.540 |  |
| bta-miR-1343-3p | 26.546 | 26.430 | 26.767 | 25.816 | 26.284 | 25.676 |  |
| bta-miR-1277 | . | 30.532 | . | . | . | . |  |
| bta-miR-1343-5p | 21.728 | 20.421 | 20.044 | 20.147 | 17.148 | 21.060 |  |
| bta-miR-1281 | 27.033 | 26.614 | 28.864 | 26.385 | 28.277 | 27.403 |  |
| bta-miR-1388-3p | 29.940 | 29.713 | 30.827 | 29.155 | 29.452 | 29.116 |  |
| bta-miR-1282 | 32.889 | 31.877 | 36.506 | 31.771 | 35.851 | 33.934 |  |
| bta-miR-1284 | 32.121 | 31.061 | 32.360 | 30.521 | 33.896 | 32.790 |  |
| bta-miR-1287 | 31.866 | 31.615 | 34.197 | 31.587 | 32.957 | 32.880 |  |
| bta-miR-1291 | 29.831 | 29.215 | 32.365 | 29.794 | 30.825 | 31.288 |  |
|  |  |  |  |  |  |  |  |

| **Supplementary Table S5:** List of miRNAs identified (Cycle Threshold) in epithelial uterine naïve cells treated with UF-EVs collected at 30 DPC from dairy cows experiencing Low or High NEB. | | | | | | | | |
| --- | --- | --- | --- | --- | --- | --- | --- | --- |
|  |  |  |  |  |  |  |  |  |
| **miRNAs** | **Group - 30 DPC** | | | | | |  |  |
|  | **Low NEB** | | | **High NEB** | | |  |  |
|  | **N334 EVs** | **N337 EVs** | **N341 EVs** | **N334 EVs** | **N341 EVs** | **N337 EVs** |  |  |
| bta-let-7a-3p | 29.175 | 30.101 | 30.459 | 29.201 | 30.586 | 29.790 |  |  |
| bta-miR-103 | 25.354 | 26.738 | 26.506 | 25.220 | 26.303 | 26.314 |  |  |
| bta-let-7a-5p | 20.746 | 21.288 | 21.344 | 20.886 | 22.246 | 21.496 |  |  |
| bta-miR-105a | 34.026 | 32.053 | . | 34.116 | . | . |  |  |
| bta-let-7b | 21.286 | 21.979 | 21.973 | 21.222 | 22.614 | 22.216 |  |  |
| bta-miR-105b | 32.647 | 33.802 | . | 33.957 | 34.582 | 35.490 |  |  |
| bta-let-7c | 20.639 | 20.963 | 21.338 | 20.662 | 21.869 | 21.447 |  |  |
| bta-miR-106a | 23.741 | 25.238 | 25.008 | 23.729 | 25.241 | 24.754 |  |  |
| bta-let-7d | 21.532 | 22.372 | 23.027 | 21.926 | 23.558 | 22.294 |  |  |
| bta-miR-106b | 25.853 | 28.256 | 27.830 | 25.931 | 27.025 | 27.723 |  |  |
| bta-let-7e | 20.802 | 21.264 | 21.254 | 20.800 | 22.090 | 21.604 |  |  |
| bta-miR-107 | 29.495 | 30.809 | 30.493 | 29.573 | 30.116 | 30.508 |  |  |
| bta-let-7f | 22.386 | 22.587 | 22.796 | 22.413 | 23.892 | 22.975 |  |  |
| bta-miR-10a | 25.797 | 26.429 | 25.737 | 25.775 | 26.962 | 26.637 |  |  |
| bta-let-7g | 24.672 | 25.437 | 25.197 | 24.816 | 26.146 | 25.485 |  |  |
| bta-miR-10b | 26.018 | 27.112 | 26.140 | 26.288 | 27.616 | 27.121 |  |  |
| bta-let-7i | 23.155 | 23.811 | 23.338 | 23.238 | 24.194 | 24.142 |  |  |
| bta-miR-122 | 35.923 | 34.277 | 34.965 | 35.106 | . | 34.256 |  |  |
| bta-miR-1 | . | . | . | . | . | . |  |  |
| bta-miR-124a | 32.437 | 31.653 | 34.393 | 32.699 | . | 35.283 |  |  |
| bta-miR-100 | 27.186 | 28.397 | 27.807 | 27.544 | 28.577 | 28.348 |  |  |
| bta-miR-124b | 32.699 | 30.829 | . | 33.905 | 35.581 | 35.660 |  |  |
| bta-miR-101 | 28.135 | 31.662 | 31.997 | 28.255 | 29.102 | 30.490 |  |  |
| bta-miR-125a | 23.753 | 24.845 | 24.280 | 23.757 | 24.799 | 24.788 |  |  |
| bta-miR-125b | 22.334 | 23.392 | 22.694 | 22.241 | 23.531 | 23.421 |  |  |
| bta-miR-133b | 33.268 | 31.686 | 33.855 | 35.386 | 35.665 | 33.186 |  |  |
| bta-miR-126-3p | 29.120 | 31.388 | 31.868 | 29.583 | 32.210 | 30.865 |  |  |
| bta-miR-133c | . | 34.630 | . | 35.223 | . | . |  |  |
| bta-miR-126-5p | 31.610 | 32.574 | 33.887 | 31.646 | 34.503 | 33.100 |  |  |
| bta-miR-134 | 35.629 | 32.815 | . | 34.755 | . | . |  |  |
| bta-miR-127 | 31.239 | 30.006 | 31.857 | 30.982 | 32.585 | 32.061 |  |  |
| bta-miR-135a | 27.587 | 28.722 | 28.013 | 27.576 | 29.780 | 28.287 |  |  |
| bta-miR-128 | 26.140 | 27.121 | 27.385 | 26.195 | 28.119 | 27.343 |  |  |
| bta-miR-135b | 28.331 | 29.488 | 30.488 | 28.821 | 30.503 | 28.830 |  |  |
| bta-miR-129 | 31.895 | 30.368 | 32.881 | 32.814 | 33.520 | 34.968 |  |  |
| bta-miR-136 | 36.095 | 35.307 | . | 36.319 | . | . |  |  |
| bta-miR-129-3p | 36.713 | 33.125 | . | 34.929 | 33.520 | 34.430 |  |  |
| bta-miR-137 | . | 36.139 | . | . | . | . |  |  |
| bta-miR-129-5p | 31.803 | 29.784 | 33.976 | 32.685 | 32.844 | 33.182 |  |  |
| bta-miR-138 | 31.594 | 29.538 | 35.194 | 32.628 | 33.958 | 35.845 |  |  |
| bta-miR-130a | 27.135 | 30.291 | 30.978 | 27.246 | 27.840 | 29.540 |  |  |
| bta-miR-139 | 33.253 | 31.326 | 33.107 | 32.854 | 36.020 | 33.109 |  |  |
| bta-miR-130b | 24.332 | 24.972 | 25.958 | 24.698 | 26.753 | 25.694 |  |  |
| bta-miR-140 | 28.590 | 30.261 | 30.486 | 28.574 | 29.429 | 30.088 |  |  |
| bta-miR-132 | 33.246 | 31.170 | 32.830 | 32.651 | 34.421 | 31.897 |  |  |
| bta-miR-141 | 24.018 | 28.351 | 28.657 | 23.982 | 25.147 | 26.505 |  |  |
| bta-miR-133a | 30.750 | 29.272 | 31.323 | 32.029 | 32.087 | 33.829 |  |  |
| bta-miR-142-3p | . | . | . | . | . | . |  |  |
| bta-miR-142-5p | 34.926 | 33.273 | 36.432 | 36.823 | 35.021 | 35.614 |  |  |
| bta-miR-151-3p | 27.444 | 27.862 | 28.033 | 27.647 | 29.244 | 28.768 |  |  |
| bta-miR-143 | 32.290 | 34.150 | 34.560 | 31.875 | 33.052 | 33.053 |  |  |
| bta-miR-151-5p | 25.369 | 25.840 | 25.949 | 25.528 | 26.946 | 26.136 |  |  |
| bta-miR-144 | . | . | . | . | . | . |  |  |
| bta-miR-152 | 34.866 | 32.772 | 33.657 | 35.670 | 34.713 | 34.116 |  |  |
| bta-miR-145 | 31.899 | 31.710 | 31.609 | 32.719 | 31.775 | 32.148 |  |  |
| bta-miR-153 | . | . | . | . | . | . |  |  |
| bta-miR-146a | 35.005 | 34.963 | 33.717 | 34.645 | . | 35.255 |  |  |
| bta-miR-154a | 33.114 | 33.972 | . | 34.148 | . | 35.188 |  |  |
| bta-miR-146b | 32.854 | 33.921 | . | 32.930 | . | 35.796 |  |  |
| bta-miR-154b | 35.863 | . | 35.333 | 35.654 | . | . |  |  |
| bta-miR-147 | 31.835 | 31.832 | 32.251 | 31.408 | 31.825 | 33.112 |  |  |
| bta-miR-154c | 34.802 | 36.515 | 35.753 | 35.271 | . | . |  |  |
| bta-miR-148a | 27.414 | 28.315 | 28.124 | 27.504 | 29.298 | 28.491 |  |  |
| bta-miR-155 | 24.892 | 26.215 | 25.637 | 24.818 | 27.288 | 25.781 |  |  |
| bta-miR-148b | 27.073 | 28.410 | 27.816 | 27.270 | 28.812 | 28.022 |  |  |
| bta-miR-15a | 25.704 | 28.141 | . | 25.795 | 27.037 | 27.806 |  |  |
| bta-miR-149-3p | 29.313 | 26.655 | 31.038 | 29.886 | 31.831 | 30.808 |  |  |
| bta-miR-15b | 25.522 | 25.538 | 25.403 | 25.662 | 26.175 | 25.561 |  |  |
| bta-miR-149-5p | 26.694 | 26.109 | 27.326 | 27.045 | 27.217 | 26.216 |  |  |
| bta-miR-16a | 23.757 | 24.968 | 24.590 | 23.808 | 25.249 | 24.914 |  |  |
| bta-miR-150 | 31.602 | 32.856 | 32.518 | 32.559 | 32.317 | 33.146 |  |  |
| bta-miR-16b | 24.798 | 25.874 | 25.815 | 24.835 | 25.970 | 25.958 |  |  |
| bta-miR-17-3p | 29.293 | 31.860 | 31.991 | 29.400 | 29.652 | 30.762 |  |  |
| bta-miR-188 | 32.537 | 32.161 | 33.243 | 33.444 | 33.114 | 35.104 |  |  |
| bta-miR-17-5p | 27.832 | 29.057 | 28.604 | 27.888 | 29.322 | 28.793 |  |  |
| bta-miR-18a | 26.486 | 28.300 | 27.758 | 26.286 | 27.925 | 27.745 |  |  |
| bta-miR-181a | 25.864 | 27.531 | 27.898 | 25.915 | 27.744 | 27.755 |  |  |
| bta-miR-18b | 28.132 | 29.657 | 28.520 | 27.782 | 28.823 | 28.808 |  |  |
| bta-miR-181b | 25.567 | 26.827 | 27.233 | 25.535 | 27.387 | 27.067 |  |  |
| bta-miR-190a | 32.911 | 35.137 | 35.762 | 33.479 | 35.051 | . |  |  |
| bta-miR-181c | 26.915 | 28.920 | 28.694 | 26.883 | 28.741 | 28.254 |  |  |
| bta-miR-190b | 31.572 | 35.444 | 36.634 | 30.837 | 35.784 | . |  |  |
| bta-miR-181d | 26.348 | 27.537 | 27.709 | 26.374 | 28.356 | 27.671 |  |  |
| bta-miR-191 | 26.440 | 27.003 | 26.978 | 26.527 | 28.142 | 27.333 |  |  |
| bta-miR-182 | 25.027 | 25.966 | 25.803 | 25.292 | 26.840 | 25.893 |  |  |
| bta-miR-192 | 30.758 | 30.765 | 31.228 | 30.460 | 31.860 | 31.442 |  |  |
| bta-miR-183 | 27.427 | 28.080 | 27.621 | 27.146 | 28.779 | 28.252 |  |  |
| bta-miR-193a | 34.515 | 33.906 | 35.500 | 35.421 | . | 36.137 |  |  |
| bta-miR-184 | 22.034 | 23.135 | 22.842 | 21.981 | 23.836 | 23.535 |  |  |
| bta-miR-193a-3p | 29.599 | 30.389 | 32.809 | 29.930 | 30.494 | 32.425 |  |  |
| bta-miR-185 | 27.812 | 29.248 | 29.193 | 27.882 | 29.432 | 29.379 |  |  |
| bta-miR-193a-5p | 27.048 | 27.210 | 27.488 | 26.946 | 28.074 | 27.557 |  |  |
| bta-miR-186 | 26.346 | 27.990 | 27.590 | 26.071 | 27.878 | 27.774 |  |  |
| bta-miR-193b | 30.109 | 31.727 | 31.330 | 30.366 | 31.072 | 31.899 |  |  |
| bta-miR-187 | 30.570 | 29.051 | 30.793 | 31.639 | 31.416 | 31.098 |  |  |
| bta-miR-194 | 28.749 | 29.768 | 30.004 | 28.805 | 30.739 | 29.841 |  |  |
| bta-miR-195 | 29.009 | 30.060 | 29.472 | 29.424 | 30.843 | 29.811 |  |  |
| bta-miR-200c | 22.698 | 23.143 | 22.913 | 22.685 | 24.244 | 23.227 |  |  |
| bta-miR-196a | 28.832 | 30.008 | 29.865 | 28.964 | 30.929 | 29.871 |  |  |
| bta-miR-202 | 36.389 | 35.808 | . | 36.679 | . | . |  |  |
| bta-miR-196b | 28.947 | 29.788 | 29.817 | 28.857 | 31.346 | 30.240 |  |  |
| bta-miR-204 | 30.911 | 31.662 | 31.876 | 31.794 | 34.794 | 31.978 |  |  |
| bta-miR-197 | 27.772 | 27.475 | 28.492 | 27.698 | 28.628 | 28.643 |  |  |
| bta-miR-205 | 22.803 | 23.910 | 23.306 | 22.836 | 23.907 | 23.898 |  |  |
| bta-miR-199a-3p | 32.980 | 32.108 | 33.893 | . | 35.262 | 31.868 |  |  |
| bta-miR-206 | 33.746 | 32.829 | 36.914 | 34.880 | . | . |  |  |
| bta-miR-199a-5p | 32.839 | 34.071 | 34.474 | 35.979 | . | 34.126 |  |  |
| bta-miR-208a | 35.950 | 34.780 | . | 34.854 | 36.793 | . |  |  |
| bta-miR-199b | 33.606 | 35.971 | 34.525 | . | . | 36.684 |  |  |
| bta-miR-208b | 35.179 | . | 35.209 | 35.847 | . | . |  |  |
| bta-miR-199c | 32.391 | 31.631 | 33.256 | 34.637 | 36.939 | 32.174 |  |  |
| bta-miR-20a | 23.836 | 25.322 | 24.951 | 23.868 | 25.430 | 24.782 |  |  |
| bta-miR-19a | 24.673 | 28.724 | 28.371 | 24.155 | 25.413 | 26.324 |  |  |
| bta-miR-20b | 24.784 | 26.243 | 26.173 | 24.753 | 26.326 | 25.794 |  |  |
| bta-miR-19b | 24.406 | 28.690 | 28.061 | 24.342 | 25.218 | 26.430 |  |  |
| bta-miR-21-3p | 31.206 | 31.399 | 34.604 | 31.833 | 32.238 | 33.897 |  |  |
| bta-miR-200a | 23.474 | 27.418 | 28.797 | 23.330 | 24.620 | 26.251 |  |  |
| bta-miR-21-5p | 26.020 | 27.148 | 25.575 | 26.176 | 27.318 | 27.150 |  |  |
| bta-miR-200b | 21.392 | 21.573 | 21.663 | 21.236 | 22.651 | 21.705 |  |  |
| bta-miR-210 | 27.826 | 28.144 | 29.732 | 28.126 | 29.374 | 30.522 |  |  |
| bta-miR-211 | 30.204 | 29.707 | 31.425 | 29.879 | 32.083 | 31.639 |  |  |
| bta-miR-22-5p | 27.498 | 28.574 | 28.223 | 27.200 | 29.030 | 28.462 |  |  |
| bta-miR-212 | 35.097 | 35.191 | . | 35.184 | . | . |  |  |
| bta-miR-221 | 22.744 | 23.780 | 23.945 | 22.770 | 24.548 | 24.085 |  |  |
| bta-miR-214 | 30.773 | 27.748 | 31.969 | 31.201 | 33.962 | 31.853 |  |  |
| bta-miR-222 | 22.543 | 23.391 | 23.820 | 22.839 | 24.293 | 23.886 |  |  |
| bta-miR-215 | 30.899 | 31.712 | 30.883 | 30.659 | 32.029 | 31.522 |  |  |
| bta-miR-223 | 35.419 | 34.001 | 34.912 | 35.427 | 36.749 | 34.529 |  |  |
| bta-miR-216a | 30.646 | 29.403 | 31.868 | 31.051 | 32.879 | 32.247 |  |  |
| bta-miR-224 | 26.604 | 27.177 | 26.314 | 26.410 | 27.689 | 27.328 |  |  |
| bta-miR-216b | 32.929 | 31.941 | 32.924 | 32.851 | 33.867 | 32.863 |  |  |
| bta-miR-23a | 20.720 | 21.014 | 20.827 | 20.786 | 22.009 | 21.229 |  |  |
| bta-miR-217 | . | 36.513 | 35.476 | . | . | . |  |  |
| bta-miR-23b-3p | 23.697 | 24.126 | 23.324 | 23.763 | 24.175 | 24.309 |  |  |
| bta-miR-218 | . | 35.590 | . | . | 36.959 | . |  |  |
| bta-miR-23b-5p | 29.765 | 30.126 | 30.703 | 29.825 | 30.763 | 30.972 |  |  |
| bta-miR-219 | 30.746 | 29.712 | 31.809 | 30.882 | 32.411 | 32.189 |  |  |
| bta-miR-24 | 31.495 | 32.841 | 35.117 | 31.772 | 31.882 | 34.087 |  |  |
| bta-miR-219-3p | 29.643 | 29.675 | 30.647 | 29.117 | 31.742 | 30.675 |  |  |
| bta-miR-24-3p | 21.387 | 22.829 | 22.751 | 21.409 | 23.075 | 22.766 |  |  |
| bta-miR-219-5p | 36.640 | . | . | . | . | 36.303 |  |  |
| bta-miR-25 | 24.919 | 25.468 | 25.153 | 24.917 | 26.210 | 25.653 |  |  |
| bta-miR-22-3p | . | . | 2.346 | 2.866 | . | 3.302 |  |  |
| bta-miR-26a | 23.171 | 24.153 | 23.700 | 23.286 | 24.773 | 24.190 |  |  |
| bta-miR-26b | 25.083 | 25.389 | 24.726 | 25.130 | 25.764 | 25.617 |  |  |
| bta-miR-29d-3p | 26.230 | 27.920 | 28.188 | 26.388 | 27.801 | 27.835 |  |  |
| bta-miR-26c | 35.364 | . | . | 35.805 | . | . |  |  |
| bta-miR-29d-5p | 30.260 | 32.061 | 32.262 | 30.199 | 31.539 | 31.830 |  |  |
| bta-miR-27a-3p | 20.807 | 22.700 | 22.583 | 21.036 | 22.776 | 22.601 |  |  |
| bta-miR-29e | 26.906 | 33.704 | 32.339 | 26.863 | 28.128 | 29.853 |  |  |
| bta-miR-27a-5p | 29.689 | 29.878 | 31.364 | 29.893 | 31.662 | 30.310 |  |  |
| bta-miR-301a | 31.581 | 35.025 | . | 30.971 | 31.696 | 34.395 |  |  |
| bta-miR-27b | 22.687 | 24.197 | 23.607 | 22.816 | 24.181 | 24.178 |  |  |
| bta-miR-301b | 31.761 | 34.504 | 36.073 | 31.100 | 32.101 | 33.076 |  |  |
| bta-miR-28 | 27.218 | 28.471 | 28.565 | 27.482 | 28.766 | 28.557 |  |  |
| bta-miR-302a | . | . | 36.753 | . | . | . |  |  |
| bta-miR-296-3p | 30.347 | 29.946 | 31.439 | 30.784 | 32.059 | 31.795 |  |  |
| bta-miR-302b | . | . | . | . | . | . |  |  |
| bta-miR-296-5p | 28.133 | 28.974 | 29.770 | 27.888 | 29.307 | 29.451 |  |  |
| bta-miR-302c | 34.626 | 33.903 | . | . | . | . |  |  |
| bta-miR-299 | . | 35.124 | 33.326 | 33.720 | . | . |  |  |
| bta-miR-302d | 36.060 | . | . | . | . | 34.841 |  |  |
| bta-miR-29a | 21.929 | 23.394 | 23.112 | 21.904 | 23.747 | 23.413 |  |  |
| bta-miR-3064 | 33.130 | 32.714 | 35.465 | 34.382 | 34.236 | 34.198 |  |  |
| bta-miR-29b | 23.676 | 29.264 | 31.366 | 23.621 | 24.731 | 27.712 |  |  |
| bta-miR-30a-5p | 25.514 | 26.926 | 26.991 | 25.437 | 27.154 | 26.840 |  |  |
| bta-miR-29c | 21.859 | 23.220 | 23.043 | 21.976 | 23.778 | 23.156 |  |  |
| bta-miR-30b-3p | 34.511 | 34.924 | 33.961 | 33.766 | . | 36.138 |  |  |
| bta-miR-30b-5p | 26.753 | 27.730 | 27.904 | 26.683 | 27.487 | 27.793 |  |  |
| bta-miR-328 | 30.174 | 29.792 | 31.589 | 30.168 | 31.744 | 31.829 |  |  |
| bta-miR-30c | 25.788 | 26.723 | 27.073 | 25.780 | 26.561 | 26.607 |  |  |
| bta-miR-329a | 34.566 | 36.114 | 36.777 | . | . | . |  |  |
| bta-miR-30d | 25.614 | 26.925 | 26.996 | 25.527 | 27.031 | 26.800 |  |  |
| bta-miR-329b | . | 33.918 | 35.494 | 36.250 | 35.931 | 35.004 |  |  |
| bta-miR-30e-5p | 25.582 | 27.060 | 26.954 | 25.406 | 26.926 | 27.035 |  |  |
| bta-miR-330 | 30.532 | 29.982 | 32.935 | 30.818 | 32.706 | 32.284 |  |  |
| bta-miR-30f | 25.875 | 26.739 | 27.649 | 25.771 | 26.650 | 26.813 |  |  |
| bta-miR-331-3p | 27.701 | 28.790 | 29.019 | 27.713 | 28.899 | 29.955 |  |  |
| bta-miR-31 | 23.806 | 25.391 | 25.662 | 24.292 | 25.943 | 25.506 |  |  |
| bta-miR-331-5p | 30.248 | 31.330 | 31.297 | 30.002 | 32.036 | 32.815 |  |  |
| bta-miR-32 | 32.360 | 35.045 | . | 31.854 | 32.576 | 34.974 |  |  |
| bta-miR-335 | 26.828 | 25.727 | 25.714 | 26.756 | 26.099 | 26.048 |  |  |
| bta-miR-320a | 25.286 | 25.837 | 25.813 | 25.143 | 27.018 | 26.407 |  |  |
| bta-miR-338 | 34.955 | . | 35.026 | 34.034 | . | 34.871 |  |  |
| bta-miR-320b | 32.876 | 30.616 | . | 33.054 | 34.240 | 33.964 |  |  |
| bta-miR-339a | 28.752 | 28.905 | 29.844 | 28.465 | 28.858 | 30.372 |  |  |
| bta-miR-323 | 32.926 | 33.353 | 34.498 | 32.436 | 35.376 | 34.721 |  |  |
| bta-miR-339b | 26.889 | 28.080 | 28.373 | 26.727 | 27.348 | 29.031 |  |  |
| bta-miR-324 | 29.818 | 30.334 | 30.911 | 29.988 | 30.739 | 31.825 |  |  |
| bta-miR-33a | 30.497 | 34.969 | . | 30.120 | 30.677 | 36.006 |  |  |
| bta-miR-326 | 30.727 | 29.314 | 34.986 | 32.014 | 32.542 | 32.654 |  |  |
| bta-miR-33b | 30.875 | 32.325 | 33.078 | 31.010 | 31.921 | 32.857 |  |  |
| bta-miR-340 | 30.035 | 31.017 | 30.505 | 29.972 | 31.810 | 31.485 |  |  |
| bta-miR-365-3p | 25.103 | 26.220 | 25.755 | 25.165 | 26.355 | 26.255 |  |  |
| bta-miR-342 | 25.704 | 25.968 | 25.371 | 25.576 | 25.366 | 25.713 |  |  |
| bta-miR-365-5p | 31.744 | 30.245 | 31.896 | 31.069 | 33.892 | 32.158 |  |  |
| bta-miR-345-3p | 31.623 | 30.936 | 33.506 | 31.841 | 32.714 | 32.607 |  |  |
| bta-miR-367 | . | . | . | . | . | . |  |  |
| bta-miR-345-5p | 29.735 | 29.977 | 31.213 | 29.206 | 29.867 | 31.666 |  |  |
| bta-miR-369-3p | 33.626 | . | . | 34.041 | 36.271 | . |  |  |
| bta-miR-346 | 29.326 | 26.793 | 29.980 | 30.788 | 31.897 | 30.836 |  |  |
| bta-miR-369-5p | . | . | . | . | . | . |  |  |
| bta-miR-34a | 24.175 | 26.145 | 26.796 | 23.827 | 25.186 | 25.746 |  |  |
| bta-miR-370 | 32.556 | 30.058 | 34.390 | 33.436 | . | 34.548 |  |  |
| bta-miR-34b | 31.781 | 34.288 | 35.039 | 31.743 | 32.878 | 33.938 |  |  |
| bta-miR-371 | 33.631 | 32.194 | 33.658 | 32.768 | 33.968 | 34.348 |  |  |
| bta-miR-34c | 31.652 | 33.843 | 34.994 | 31.727 | 33.825 | 33.976 |  |  |
| bta-miR-374a | 25.988 | 27.094 | 26.937 | 25.999 | 28.468 | 26.747 |  |  |
| bta-miR-361 | 26.548 | 26.942 | 26.776 | 26.188 | 28.451 | 27.009 |  |  |
| bta-miR-374b | 27.128 | 27.770 | 27.754 | 27.417 | 29.253 | 27.878 |  |  |
| bta-miR-362-3p | 29.992 | 31.760 | 31.164 | 29.823 | 30.861 | 30.458 |  |  |
| bta-miR-375 | 27.240 | 27.373 | 27.291 | 27.384 | 29.607 | 27.418 |  |  |
| bta-miR-362-5p | 29.593 | 30.541 | 31.899 | 29.075 | 30.838 | 29.804 |  |  |
| bta-miR-376a | 33.745 | 36.644 | 35.037 | 34.820 | 34.858 | 35.229 |  |  |
| bta-miR-363 | 31.162 | 31.255 | 33.900 | 31.104 | 32.675 | 32.785 |  |  |
| bta-miR-376b | . | 36.717 | . | 36.407 | . | . |  |  |
| bta-miR-376c | 34.892 | . | . | 35.643 | 35.053 | . |  |  |
| bta-miR-382 | 32.712 | 33.493 | 34.800 | 33.557 | 35.421 | 36.437 |  |  |
| bta-miR-376d | 32.938 | 34.361 | 34.377 | 33.915 | . | . |  |  |
| bta-miR-383 | 32.783 | 31.641 | 34.261 | 35.075 | . | 34.588 |  |  |
| bta-miR-376e | 33.912 | . | 35.008 | 36.080 | 36.718 | . |  |  |
| bta-miR-409a | 35.258 | 35.737 | 35.449 | . | 35.453 | . |  |  |
| bta-miR-377 | 32.358 | 34.834 | . | 32.220 | 34.963 | 35.658 |  |  |
| bta-miR-409b | . | 36.058 | 34.374 | 35.012 | . | 34.960 |  |  |
| bta-miR-378 | 24.977 | 26.055 | 25.893 | 24.938 | 25.909 | 25.755 |  |  |
| bta-miR-410 | 33.800 | 33.189 | . | 34.316 | . | 34.967 |  |  |
| bta-miR-378b | 24.894 | 25.777 | 26.000 | 25.020 | 25.838 | 25.838 |  |  |
| bta-miR-411a | 32.671 | . | 34.387 | 32.050 | 34.074 | . |  |  |
| bta-miR-378c | 30.789 | 31.786 | 29.823 | 30.815 | 30.132 | 31.771 |  |  |
| bta-miR-411b | . | . | 35.883 | 34.900 | . | . |  |  |
| bta-miR-378d | 31.868 | 32.371 | 32.903 | 31.885 | 32.938 | 32.816 |  |  |
| bta-miR-411c-3p | 32.927 | 32.577 | . | 33.239 | . | . |  |  |
| bta-miR-379 | 31.827 | 32.947 | 33.382 | 33.083 | 32.140 | 36.135 |  |  |
| bta-miR-411c-5p | . | . | . | . | . | . |  |  |
| bta-miR-380-3p | 31.743 | 32.187 | 33.662 | 32.199 | 32.352 | 33.990 |  |  |
| bta-miR-412 | 30.725 | 30.912 | 33.992 | 28.962 | 33.613 | 32.612 |  |  |
| bta-miR-380-5p | 34.765 | 34.982 | 35.673 | 34.533 | 35.810 | 36.195 |  |  |
| bta-miR-421 | 28.235 | 29.629 | 26.915 | 27.639 | 26.243 | 28.808 |  |  |
| bta-miR-381 | 32.493 | 31.926 | 36.621 | 33.136 | 34.862 | 36.885 |  |  |
| bta-miR-423-3p | 26.684 | 27.739 | 27.638 | 26.788 | 28.253 | 27.936 |  |  |
| bta-miR-423-5p | 25.450 | 26.068 | 27.150 | 25.445 | 27.221 | 26.660 |  |  |
| bta-miR-449c | 31.878 | 32.279 | . | 31.008 | 32.690 | 34.022 |  |  |
| bta-miR-424-3p | 32.883 | 31.803 | 32.673 | 32.447 | 32.760 | 33.430 |  |  |
| bta-miR-449d | 30.037 | 27.823 | 30.626 | 29.931 | 30.732 | 29.761 |  |  |
| bta-miR-424-5p | 28.742 | 30.620 | 29.851 | 28.759 | 31.419 | 30.788 |  |  |
| bta-miR-450a | 28.781 | 29.822 | 28.659 | 27.437 | 30.769 | 29.704 |  |  |
| bta-miR-425-3p | 26.950 | 25.289 | 28.253 | 27.700 | 27.705 | 27.783 |  |  |
| bta-miR-450b | 31.068 | 33.621 | 29.781 | 30.243 | 32.121 | 31.899 |  |  |
| bta-miR-425-5p | 26.749 | 27.619 | 27.553 | 27.040 | 28.824 | 27.831 |  |  |
| bta-miR-451 | 31.616 | 32.964 | . | 36.240 | . | 35.310 |  |  |
| bta-miR-429 | 25.537 | 26.561 | 25.701 | 25.556 | 27.112 | 26.508 |  |  |
| bta-miR-452 | 32.944 | 33.069 | 31.233 | 32.911 | 32.442 | 33.425 |  |  |
| bta-miR-431 | 31.840 | 29.783 | 32.711 | 31.898 | 32.111 | 32.483 |  |  |
| bta-miR-4523 | 32.886 | 30.752 | 34.503 | 33.645 | 34.480 | 33.808 |  |  |
| bta-miR-432 | 32.791 | 31.692 | 34.972 | 33.623 | 34.346 | 36.607 |  |  |
| bta-miR-453 | 33.727 | 31.982 | 34.137 | 33.178 | 33.813 | 33.519 |  |  |
| bta-miR-433 | 31.671 | 30.689 | 33.082 | 32.130 | 33.973 | 32.857 |  |  |
| bta-miR-454 | 29.102 | 29.599 | 28.797 | 29.224 | 30.016 | 29.831 |  |  |
| bta-miR-448 | 36.486 | . | . | . | . | . |  |  |
| bta-miR-455-3p | 32.155 | 32.794 | 30.895 | 31.474 | 32.269 | 32.284 |  |  |
| bta-miR-449a | 26.636 | 28.455 | 30.176 | 26.559 | 28.899 | 28.832 |  |  |
| bta-miR-455-5p | 31.299 | 31.851 | 32.790 | 30.639 | 32.115 | 31.704 |  |  |
| bta-miR-449b | 34.143 | 33.001 | 34.294 | 33.577 | 34.981 | 34.172 |  |  |
| bta-miR-483 | 32.912 | 31.909 | 35.509 | 33.882 | 33.146 | 34.951 |  |  |
| bta-miR-484 | 26.982 | 27.500 | 27.463 | 26.773 | 27.702 | 27.678 |  |  |
| bta-miR-496 | 34.990 | 33.274 | 35.862 | 35.014 | 34.841 | 33.956 |  |  |
| bta-miR-485 | 33.516 | 35.968 | . | 33.778 | . | 36.390 |  |  |
| bta-miR-497 | 29.078 | 29.803 | 31.472 | 29.592 | 30.959 | 30.286 |  |  |
| bta-miR-486 | 31.642 | 29.176 | 32.271 | 30.728 | 32.014 | 31.814 |  |  |
| bta-miR-499 | 31.249 | 31.851 | 31.104 | 30.825 | . | 31.651 |  |  |
| bta-miR-487a | 33.791 | 32.743 | 36.794 | 33.984 | 36.512 | 35.655 |  |  |
| bta-miR-500 | 28.825 | 29.784 | 29.882 | 28.729 | 31.153 | 29.791 |  |  |
| bta-miR-487b | 32.777 | 34.120 | 35.170 | 33.427 | . | 36.911 |  |  |
| bta-miR-502a | 29.482 | 33.422 | 35.040 | 28.808 | 33.728 | 32.708 |  |  |
| bta-miR-488 | 34.103 | 33.327 | . | . | . | 33.968 |  |  |
| bta-miR-502b | 31.147 | 31.891 | 32.360 | 31.756 | 34.089 | 32.171 |  |  |
| bta-miR-489 | 36.855 | . | . | 35.630 | . | . |  |  |
| bta-miR-503-3p | 29.836 | 28.678 | 32.409 | 30.139 | 31.358 | 31.412 |  |  |
| bta-miR-490 | 32.257 | 30.742 | 33.278 | 32.655 | 34.620 | 33.899 |  |  |
| bta-miR-503-5p | 30.308 | 32.427 | 33.074 | 30.924 | 31.963 | 32.821 |  |  |
| bta-miR-491 | 30.135 | 31.696 | 33.647 | 30.154 | 31.802 | 32.479 |  |  |
| bta-miR-504 | 33.783 | 32.167 | 33.199 | 31.908 | . | 33.214 |  |  |
| bta-miR-493 | 30.840 | 28.944 | 33.687 | 31.828 | 32.355 | 33.343 |  |  |
| bta-miR-505 | 29.089 | 28.576 | 29.679 | 28.736 | 30.557 | 29.751 |  |  |
| bta-miR-494 | 26.361 | 23.177 | 27.729 | 27.013 | 28.344 | 28.108 |  |  |
| bta-miR-532 | 29.976 | 31.272 | 31.603 | 29.872 | 31.660 | 31.320 |  |  |
| bta-miR-495 | 33.729 | . | 33.967 | 33.735 | 34.612 | 35.510 |  |  |
| bta-miR-539 | 34.654 | 36.943 | . | . | . | 35.688 |  |  |
| bta-miR-541 | 31.701 | 30.038 | 32.875 | 32.644 | 33.784 | 32.775 |  |  |
| bta-miR-582 | 30.033 | 29.653 | 32.698 | 29.885 | 31.823 | 30.442 |  |  |
| bta-miR-542-5p | 32.870 | 33.930 | . | 32.752 | . | . |  |  |
| bta-miR-584 | 30.806 | 30.339 | 32.913 | 31.160 | 33.136 | 31.923 |  |  |
| bta-miR-543 | 34.730 | 33.341 | 34.079 | 33.950 | 34.277 | 34.965 |  |  |
| bta-miR-592 | . | 34.981 | 34.761 | 34.999 | 35.881 | 34.193 |  |  |
| bta-miR-544a | 34.888 | 34.694 | . | 33.913 | . | 35.037 |  |  |
| bta-miR-599 | . | . | . | . | . | . |  |  |
| bta-miR-544b | . | . | . | . | . | . |  |  |
| bta-miR-615 | 5.848 | 5.820 | 7.382 | 5.816 | 6.705 | 5.811 |  |  |
| bta-miR-545-3p | 31.986 | 33.922 | 34.211 | 32.780 | 32.886 | 34.642 |  |  |
| bta-miR-628 | 33.960 | 34.939 | . | . | . | 34.708 |  |  |
| bta-miR-545-5p | 31.110 | . | 34.001 | 31.808 | 34.320 | 34.507 |  |  |
| bta-miR-631 | 32.589 | 29.247 | 32.718 | 32.538 | 33.267 | 33.461 |  |  |
| bta-miR-551a | 34.581 | 36.047 | 36.372 | . | . | 35.577 |  |  |
| bta-miR-652 | 26.783 | 27.539 | 27.087 | 26.558 | 27.816 | 27.499 |  |  |
| bta-miR-551b | . | 36.700 | . | 36.354 | . | 35.684 |  |  |
| bta-miR-653 | 35.628 | . | . | . | . | . |  |  |
| bta-miR-562 | 34.051 | 34.353 | 35.795 | . | 34.880 | 35.928 |  |  |
| bta-miR-654 | 20.643 | 20.442 | 21.357 | 20.947 | 21.349 | 20.309 |  |  |
| bta-miR-568 | 32.627 | 31.832 | . | 32.723 | 33.961 | 32.556 |  |  |
| bta-miR-655 | . | . | . | 35.457 | . | . |  |  |
| bta-miR-574 | 24.488 | 23.743 | 25.758 | 25.545 | 27.110 | 26.483 |  |  |
| bta-miR-656 | . | 34.267 | . | 36.807 | . | 35.663 |  |  |
| bta-miR-658 | 34.401 | 33.094 | 35.381 | 34.512 | . | . |  |  |
| bta-miR-758 | 33.064 | 31.763 | 35.037 | 35.038 | 32.926 | 36.061 |  |  |
| bta-miR-660 | 28.332 | 28.833 | 28.788 | 28.223 | 30.216 | 28.616 |  |  |
| bta-miR-759 | . | . | . | . | . | . |  |  |
| bta-miR-664a | 31.619 | 29.021 | 32.625 | 32.381 | 33.866 | 32.973 |  |  |
| bta-miR-760-3p | 31.357 | 30.189 | 31.766 | 31.648 | 32.854 | 32.704 |  |  |
| bta-miR-664b | 24.803 | 24.643 | 24.840 | 25.210 | 25.037 | 24.808 |  |  |
| bta-miR-760-5p | 25.829 | 25.782 | 28.662 | 26.807 | 27.840 | 28.153 |  |  |
| bta-miR-665 | 28.893 | 26.559 | 29.465 | 29.146 | 29.918 | 29.780 |  |  |
| bta-miR-761 | 33.234 | 32.732 | 33.709 | 33.923 | 34.449 | 35.439 |  |  |
| bta-miR-669 | 26.775 | 25.832 | 28.933 | 27.764 | 29.821 | 28.486 |  |  |
| bta-miR-763 | 30.902 | 29.747 | 32.793 | 31.882 | 33.223 | 33.306 |  |  |
| bta-miR-670 | 35.467 | 35.118 | . | 35.884 | . | . |  |  |
| bta-miR-764 | 34.197 | 33.760 | . | . | . | . |  |  |
| bta-miR-671 | 32.738 | 30.931 | 32.936 | 33.017 | 34.304 | 33.841 |  |  |
| bta-miR-767 | 33.307 | 33.778 | 33.632 | 35.089 | 33.958 | 34.543 |  |  |
| bta-miR-677 | 26.111 | 27.323 | 29.382 | 26.982 | 28.249 | 29.046 |  |  |
| bta-miR-769 | 32.065 | 32.223 | 33.994 | 32.290 | 32.885 | 34.373 |  |  |
| bta-miR-7 | 28.482 | 28.568 | 27.943 | 28.684 | 29.806 | 28.666 |  |  |
| bta-miR-873 | 35.316 | 32.815 | 35.965 | 34.415 | 35.024 | 36.964 |  |  |
| bta-miR-708 | 28.618 | 30.281 | 30.894 | 28.337 | 31.188 | 30.025 |  |  |
| bta-miR-874 | 31.041 | 29.224 | 33.633 | 31.696 | 35.280 | 33.061 |  |  |
| bta-miR-744 | 29.777 | 30.033 | 30.577 | 29.824 | 30.592 | 31.033 |  |  |
| bta-miR-875 | . | . | . | 35.573 | . | 35.033 |  |  |
| bta-miR-876 | 35.876 | 33.960 | . | 35.887 | 36.833 | . |  |  |
| bta-miR-98 | 27.135 | 27.773 | 27.034 | 27.460 | 28.647 | 28.062 |  |  |
| bta-miR-877 | 28.737 | 28.450 | 29.774 | 29.331 | 30.491 | 29.733 |  |  |
| bta-miR-99a-3p | 33.478 | . | . | 33.833 | 34.227 | 36.494 |  |  |
| bta-miR-885 | 30.927 | 29.679 | 30.623 | 31.003 | 30.529 | 30.454 |  |  |
| bta-miR-99a-5p | 27.633 | 28.936 | 27.995 | 27.960 | 29.291 | 28.840 |  |  |
| bta-miR-9-3p | . | . | . | . | . | . |  |  |
|  |  |  |  |  |  |  |  |  |
| bta-miR-9-5p | 28.372 | 27.627 | 27.557 | 28.530 | 28.900 | 27.560 |  |  |
| bta-miR-1179 | 36.324 | . | 36.359 | 36.414 | . | . |  |  |
| bta-miR-92a | 23.601 | 23.906 | 23.804 | 23.687 | 24.922 | 24.203 |  |  |
| bta-miR-1185 | . | . | . | . | . | 36.081 |  |  |
| bta-miR-92b | 25.800 | 25.817 | 26.894 | 25.910 | 25.813 | 26.539 |  |  |
| bta-miR-1193 | . | 35.161 | . | . | . | . |  |  |
| bta-miR-93 | 24.824 | 26.043 | 25.824 | 24.806 | 26.227 | 25.798 |  |  |
| bta-miR-1197 | . | . | . | . | 36.853 | . |  |  |
| bta-miR-935 | 17.809 | 17.829 | 18.330 | 18.010 | 17.944 | 17.816 |  |  |
| bta-miR-122 | 34.257 | 32.892 | 35.626 | 34.420 | 35.219 | 34.343 |  |  |
| bta-miR-940 | 22.684 | 23.575 | 24.002 | 22.639 | 23.248 | 23.325 |  |  |
| bta-miR-1224 | 26.300 | 24.061 | 27.770 | 26.928 | 27.956 | 27.637 |  |  |
| bta-miR-95 | 31.983 | 32.893 | 33.979 | 32.833 | 32.839 | 32.777 |  |  |
| bta-miR-1225-3p | 24.951 | 24.387 | 26.196 | 25.773 | 26.256 | 26.617 |  |  |
| bta-miR-96 | 25.830 | 28.449 | 28.787 | 26.312 | 27.766 | 28.070 |  |  |
| bta-miR-1246 | 20.736 | 20.793 | 21.674 | 21.812 | 22.786 | 22.761 |  |  |
| bta-miR-1247-3p | 30.931 | 29.042 | 31.687 | 30.747 | 31.840 | 31.711 |  |  |
| bta-miR-1296 | 29.673 | 28.891 | 32.083 | 30.312 | 32.927 | 31.868 |  |  |
| bta-miR-1247-5p | 27.917 | 27.335 | 29.363 | 28.215 | 28.794 | 29.537 |  |  |
| bta-miR-1298 | . | 35.147 | . | . | . | . |  |  |
| bta-miR-1248 | 31.919 | 30.336 | 33.791 | 32.860 | 34.905 | 33.840 |  |  |
| bta-miR-1301 | 35.051 | 32.064 | . | 33.977 | . | 35.296 |  |  |
| bta-miR-1249 | 28.389 | 28.692 | 27.844 | 28.066 | 27.727 | 28.386 |  |  |
| bta-miR-1306 | 29.138 | 29.305 | 28.654 | 29.065 | 28.681 | 29.120 |  |  |
| bta-miR-1260b | 22.265 | 22.626 | 23.077 | 22.343 | 22.246 | 22.821 |  |  |
| bta-miR-1307 | 26.979 | 24.369 | 28.673 | 27.679 | 29.280 | 29.225 |  |  |
| bta-miR-1271 | 30.954 | 32.661 | 33.678 | 30.736 | 33.234 | 32.614 |  |  |
| bta-miR-1343-3p | 28.830 | 27.797 | 29.190 | 28.791 | 29.768 | 29.967 |  |  |
| bta-miR-1277 | . | . | . | . | . | . |  |  |
| bta-miR-1343-5p | 25.871 | 23.816 | 27.630 | 26.807 | 28.117 | 28.431 |  |  |
| bta-miR-1281 | 28.173 | 27.630 | 30.108 | 29.242 | 29.947 | 29.997 |  |  |
| bta-miR-1388-3p | 30.748 | 29.529 | 31.853 | 30.727 | 31.814 | 30.974 |  |  |
| bta-miR-1282 | 33.764 | 33.845 | . | 36.098 | 35.408 | 34.359 |  |  |
| bta-miR-1284 | 33.850 | 31.853 | 35.935 | 33.959 | 34.981 | 35.918 |  |  |
| bta-miR-1287 | 33.906 | 31.923 | 33.609 | 34.999 | 36.074 | 33.996 |  |  |
| bta-miR-1291 | 33.822 | 31.885 | . | 34.287 | 35.858 | 34.722 |  |  |

| **Supplementary Table S6:** List of miRNAs identified (Cycle Threshold) in epithelial uterine naïve cells treated with UF-EVs collected at 60 DPC from dairy cows experiencing Low or High NEB. | | | | | | | |
| --- | --- | --- | --- | --- | --- | --- | --- |
|  |  |  |  |  |  |  |  |
| **miRNAs** | **Group - 60 DPC** | | | | | |  |
|  | **Low NEB** | | | **High NEB** | | |  |
|  | **N337 EVs** | **N341 EVs** | **N334 EVs** | **N337 EVs** | **N334 EVs** | **N341 EVs** |  |
| bta-let-7a-3p | . | 29.401 | 29.128 | 30.055 | 30.240 | 29.203 |  |
| bta-miR-103 | 32.867 | 25.072 | 25.457 | 27.278 | 26.787 | 24.908 |  |
| bta-let-7a-5p | 25.080 | 20.754 | 21.152 | 21.370 | 22.617 | 21.127 |  |
| bta-miR-105a | 33.048 | 34.474 | 35.754 | 33.793 | . | . |  |
| bta-let-7b | 25.761 | 21.536 | 21.389 | 22.297 | 23.525 | 21.843 |  |
| bta-miR-105b | 35.850 | 33.988 | 33.359 | 36.564 | 35.689 | 34.083 |  |
| bta-let-7c | 24.793 | 20.785 | 20.695 | 21.509 | 22.582 | 21.637 |  |
| bta-miR-106a | 33.825 | 23.702 | 23.793 | 25.801 | 24.768 | 23.455 |  |
| bta-let-7d | 26.017 | 21.791 | 22.393 | 22.640 | 24.475 | 22.607 |  |
| bta-miR-106b | 32.913 | 25.680 | 25.921 | 29.427 | 27.413 | 25.750 |  |
| bta-let-7e | 24.788 | 20.834 | 20.801 | 21.562 | 22.676 | 20.824 |  |
| bta-miR-107 | 33.992 | 28.808 | 29.587 | 30.722 | 30.715 | 28.762 |  |
| bta-let-7f | 26.625 | 22.422 | 22.562 | 22.834 | 24.213 | 22.658 |  |
| bta-miR-10a | 32.489 | 25.710 | 25.743 | 26.668 | 26.829 | 25.488 |  |
| bta-let-7g | 30.863 | 24.584 | 24.775 | 25.742 | 26.418 | 24.537 |  |
| bta-miR-10b | 32.539 | 26.128 | 26.183 | 27.217 | 27.387 | 26.025 |  |
| bta-let-7i | 29.990 | 22.699 | 23.327 | 24.126 | 25.772 | 22.709 |  |
| bta-miR-122 | 34.619 | 33.431 | 33.892 | 35.269 | 35.911 | . |  |
| bta-miR-1 | 35.845 | . | . | 34.659 | . | . |  |
| bta-miR-124a | 34.948 | 35.065 | 33.931 | 33.947 | . | 36.995 |  |
| bta-miR-100 | 35.826 | 27.452 | 27.509 | 28.709 | 28.669 | 27.143 |  |
| bta-miR-124b | 36.476 | . | 33.945 | 33.853 | 36.091 | . |  |
| bta-miR-101 | 35.493 | 27.993 | 28.043 | 32.938 | 29.121 | 28.231 |  |
| bta-miR-125a | 29.812 | 23.727 | 23.786 | 25.031 | 24.907 | 23.798 |  |
| bta-miR-125b | 28.746 | 22.310 | 22.315 | 23.798 | 23.667 | 22.291 |  |
| bta-miR-133b | 33.351 | 34.027 | 36.404 | 32.872 | 35.463 | . |  |
| bta-miR-126-3p | . | 30.833 | 29.382 | 31.876 | 31.139 | 31.701 |  |
| bta-miR-133c | 35.754 | . | . | 35.033 | . | . |  |
| bta-miR-126-5p | . | 33.491 | 31.820 | 32.995 | 32.679 | 32.169 |  |
| bta-miR-134 | 32.872 | 36.437 | 34.382 | 35.847 | . | . |  |
| bta-miR-127 | 32.506 | 31.031 | 31.627 | 32.066 | 33.064 | 31.301 |  |
| bta-miR-135a | 33.948 | 28.056 | 27.588 | 28.129 | 29.272 | 27.999 |  |
| bta-miR-128 | 32.169 | 26.409 | 26.151 | 27.215 | 28.209 | 26.728 |  |
| bta-miR-135b | 34.298 | 28.712 | 28.774 | 28.886 | 30.316 | 29.009 |  |
| bta-miR-129 | 32.644 | 32.598 | 32.834 | 31.792 | 36.023 | 32.508 |  |
| bta-miR-136 | . | 35.705 | . | 36.465 | . | . |  |
| bta-miR-129-3p | 35.977 | 33.889 | 34.646 | 33.962 | 35.610 | 34.356 |  |
| bta-miR-137 | . | . | . | . | . | . |  |
| bta-miR-129-5p | 32.573 | 32.055 | 32.846 | 32.816 | 35.826 | 33.458 |  |
| bta-miR-138 | 29.593 | 32.928 | 35.307 | 30.139 | . | 32.891 |  |
| bta-miR-130a | 32.302 | 27.168 | 27.433 | 30.597 | 30.531 | 27.705 |  |
| bta-miR-139 | 33.579 | 32.677 | 32.886 | 31.782 | 34.657 | 31.821 |  |
| bta-miR-130b | 29.672 | 24.797 | 25.121 | 25.084 | 28.251 | 25.526 |  |
| bta-miR-140 | 35.855 | 28.693 | 28.672 | 31.330 | 29.683 | 28.551 |  |
| bta-miR-132 | 34.918 | 33.022 | 32.947 | 32.125 | 35.028 | 33.786 |  |
| bta-miR-141 | . | 24.174 | 24.065 | 29.758 | 26.695 | 24.412 |  |
| bta-miR-133a | 31.109 | 31.903 | 32.827 | 31.157 | 33.924 | 31.767 |  |
| bta-miR-142-3p | . | . | . | . | . | . |  |
| bta-miR-142-5p | 35.424 | 35.155 | 36.363 | 35.351 | 35.085 | 35.240 |  |
| bta-miR-151-3p | 30.154 | 27.757 | 27.729 | 28.218 | 29.213 | 27.890 |  |
| bta-miR-143 | 36.351 | 31.809 | 32.909 | 35.003 | 33.961 | 31.875 |  |
| bta-miR-151-5p | 31.535 | 25.428 | 25.678 | 26.467 | 26.743 | 25.827 |  |
| bta-miR-144 | . | . | . | . | . | . |  |
| bta-miR-152 | 35.088 | 33.519 | 33.886 | 35.018 | 34.948 | 32.969 |  |
| bta-miR-145 | 35.020 | 30.603 | 33.912 | 33.376 | 36.707 | 31.293 |  |
| bta-miR-153 | . | 34.875 | . | . | . | . |  |
| bta-miR-146a | . | . | 32.975 | 34.440 | 33.914 | 34.658 |  |
| bta-miR-154a | 33.759 | 33.885 | 33.600 | 33.927 | . | . |  |
| bta-miR-146b | 36.009 | 33.994 | 33.159 | 32.940 | 34.989 | . |  |
| bta-miR-154b | . | 35.710 | 34.227 | . | 34.762 | 35.633 |  |
| bta-miR-147 | 31.827 | 30.599 | 31.048 | 32.772 | 34.348 | 32.095 |  |
| bta-miR-154c | 36.014 | . | 36.718 | 34.847 | 36.610 | 35.664 |  |
| bta-miR-148a | 32.898 | 27.640 | 27.350 | 28.731 | 29.008 | 27.930 |  |
| bta-miR-155 | 29.039 | 25.273 | 24.814 | 25.741 | 26.750 | 25.332 |  |
| bta-miR-148b | 33.119 | 27.656 | 27.107 | 28.743 | 29.046 | 27.595 |  |
| bta-miR-15a | . | 25.860 | 25.879 | 28.726 | 28.789 | 26.346 |  |
| bta-miR-149-3p | 29.752 | 30.357 | 30.108 | 29.495 | 34.302 | 31.181 |  |
| bta-miR-15b | 30.303 | 24.850 | 25.746 | 25.782 | 26.708 | 24.891 |  |
| bta-miR-149-5p | 24.958 | 27.306 | 26.980 | 24.892 | 28.130 | 27.469 |  |
| bta-miR-16a | 31.985 | 23.768 | 23.904 | 25.360 | 25.797 | 23.833 |  |
| bta-miR-150 | . | 33.798 | 33.521 | 33.905 | 34.356 | 36.629 |  |
| bta-miR-16b | 32.904 | 24.363 | 24.761 | 26.206 | 26.559 | 24.544 |  |
| bta-miR-17-3p | 34.945 | 29.737 | 29.188 | 31.980 | 31.441 | 29.624 |  |
| bta-miR-188 | 35.026 | 32.643 | 33.373 | 33.237 | 35.335 | 33.074 |  |
| bta-miR-17-5p | 36.304 | 27.728 | 27.907 | 29.728 | 29.016 | 27.639 |  |
| bta-miR-18a | 34.284 | 26.504 | 26.421 | 28.734 | 27.409 | 26.259 |  |
| bta-miR-181a | 33.558 | 26.363 | 26.267 | 28.567 | 28.999 | 27.033 |  |
| bta-miR-18b | 35.017 | 27.851 | 27.895 | 29.950 | 28.761 | 27.373 |  |
| bta-miR-181b | 33.069 | 25.907 | 25.548 | 27.725 | 28.581 | 26.406 |  |
| bta-miR-190a | . | 33.372 | 33.564 | . | 35.595 | 34.309 |  |
| bta-miR-181c | 35.869 | 27.480 | 27.294 | 29.603 | 30.032 | 28.137 |  |
| bta-miR-190b | . | 31.430 | 30.442 | 36.079 | 30.361 | 29.780 |  |
| bta-miR-181d | 32.945 | 26.970 | 26.556 | 27.717 | 29.513 | 27.574 |  |
| bta-miR-191 | 30.652 | 26.614 | 26.551 | 27.415 | 27.706 | 26.597 |  |
| bta-miR-182 | 31.250 | 25.685 | 25.391 | 26.365 | 26.498 | 25.567 |  |
| bta-miR-192 | 34.698 | 30.670 | 31.078 | 31.846 | 31.895 | 30.742 |  |
| bta-miR-183 | 32.243 | 27.399 | 27.419 | 28.220 | 28.927 | 27.550 |  |
| bta-miR-193a | 34.329 | 34.301 | 35.295 | . | . | 36.617 |  |
| bta-miR-184 | 27.156 | 22.491 | 22.115 | 23.320 | 23.638 | 22.372 |  |
| bta-miR-193a-3p | 33.942 | 29.747 | 30.386 | 32.437 | 32.458 | 30.629 |  |
| bta-miR-185 | 33.887 | 28.144 | 27.897 | 29.832 | 29.614 | 28.105 |  |
| bta-miR-193a-5p | 31.055 | 27.117 | 27.036 | 27.738 | 28.172 | 27.193 |  |
| bta-miR-186 | 33.317 | 26.458 | 26.092 | 28.523 | 28.072 | 26.531 |  |
| bta-miR-193b | 35.685 | 30.632 | 30.970 | 31.814 | 32.887 | 30.985 |  |
| bta-miR-187 | 30.770 | 30.540 | 31.838 | 30.504 | 33.917 | 31.980 |  |
| bta-miR-194 | 36.187 | 29.073 | 28.961 | 29.874 | 30.748 | 29.654 |  |
| bta-miR-195 | . | 28.863 | 29.589 | 30.303 | 30.217 | 28.978 |  |
| bta-miR-200c | 28.668 | 22.765 | 22.768 | 23.339 | 23.988 | 22.762 |  |
| bta-miR-196a | 35.485 | 29.369 | 29.389 | 29.873 | 30.838 | 29.766 |  |
| bta-miR-202 | . | . | . | . | . | . |  |
| bta-miR-196b | 35.061 | 29.661 | 29.762 | 30.202 | 31.623 | 29.817 |  |
| bta-miR-204 | 33.096 | 32.004 | 30.791 | 31.499 | 31.693 | 32.172 |  |
| bta-miR-197 | 30.540 | 27.983 | 27.729 | 28.645 | 28.635 | 27.931 |  |
| bta-miR-205 | 29.787 | 22.601 | 23.021 | 24.312 | 24.802 | 22.653 |  |
| bta-miR-199a-3p | . | . | . | 34.690 | . | 35.256 |  |
| bta-miR-206 | 35.347 | 33.558 | 35.336 | 33.957 | . | . |  |
| bta-miR-199a-5p | 36.625 | . | . | 34.996 | . | . |  |
| bta-miR-208a | 35.638 | 36.213 | . | 35.481 | . | . |  |
| bta-miR-199b | . | . | . | . | . | 35.514 |  |
| bta-miR-208b | . | 35.034 | . | 35.346 | 36.491 | 35.704 |  |
| bta-miR-199c | . | 35.558 | 36.450 | 33.387 | . | 34.214 |  |
| bta-miR-20a | 31.929 | 23.627 | 24.029 | 25.776 | 24.879 | 23.637 |  |
| bta-miR-19a | 34.404 | 24.379 | 23.960 | 29.918 | 24.809 | 23.707 |  |
| bta-miR-20b | 32.891 | 24.705 | 24.825 | 26.838 | 25.727 | 24.675 |  |
| bta-miR-19b | 34.787 | 24.344 | 23.976 | 29.676 | 24.800 | 23.611 |  |
| bta-miR-21-3p | 32.909 | 31.813 | 32.350 | 33.160 | 35.422 | 33.621 |  |
| bta-miR-200a | 33.982 | 23.710 | 23.478 | 28.877 | 26.578 | 24.229 |  |
| bta-miR-21-5p | 30.577 | 26.028 | 26.073 | 27.122 | 27.906 | 25.789 |  |
| bta-miR-200b | 27.425 | 21.418 | 21.566 | 21.766 | 22.357 | 21.370 |  |
| bta-miR-210 | 32.665 | 28.307 | 28.546 | 30.161 | 30.460 | 28.733 |  |
| bta-miR-211 | 33.018 | 31.324 | 29.801 | 30.293 | 31.133 | 30.814 |  |
| bta-miR-22-5p | 34.042 | 27.761 | 27.524 | 28.960 | 29.805 | 27.997 |  |
| bta-miR-212 | 35.276 | 35.641 | . | 35.507 | . | 36.826 |  |
| bta-miR-221 | 30.151 | 22.900 | 22.952 | 24.557 | 26.042 | 23.669 |  |
| bta-miR-214 | 32.235 | 31.296 | 31.615 | 31.297 | 34.823 | 32.133 |  |
| bta-miR-222 | 29.113 | 22.882 | 22.885 | 23.993 | 25.626 | 23.585 |  |
| bta-miR-215 | . | 30.933 | 30.733 | 31.658 | 31.403 | 30.313 |  |
| bta-miR-223 | 33.932 | 34.114 | 36.111 | 35.666 | 36.255 | 34.784 |  |
| bta-miR-216a | 35.409 | 30.976 | 31.761 | 31.236 | 33.704 | 30.869 |  |
| bta-miR-224 | 34.826 | 26.065 | 26.307 | 27.037 | 27.809 | 25.806 |  |
| bta-miR-216b | 33.171 | 32.474 | 32.882 | 32.302 | 35.803 | 34.108 |  |
| bta-miR-23a | 27.649 | 20.756 | 20.806 | 21.471 | 22.269 | 20.772 |  |
| bta-miR-217 | . | . | . | . | . | . |  |
| bta-miR-23b-3p | 27.581 | 23.473 | 23.827 | 24.535 | 25.477 | 23.411 |  |
| bta-miR-218 | . | . | . | . | . | . |  |
| bta-miR-23b-5p | 32.101 | 29.993 | 29.727 | 30.539 | 33.289 | 30.056 |  |
| bta-miR-219 | 31.809 | 31.709 | 32.114 | 30.526 | 33.169 | 31.889 |  |
| bta-miR-24 | . | 32.467 | 32.081 | 34.514 | 34.230 | 32.224 |  |
| bta-miR-219-3p | 31.854 | 29.142 | 29.767 | 30.288 | 32.883 | 30.278 |  |
| bta-miR-24-3p | 28.639 | 21.384 | 21.310 | 23.193 | 23.104 | 21.646 |  |
| bta-miR-219-5p | . | . | . | . | 36.865 | 36.029 |  |
| bta-miR-25 | 30.719 | 24.792 | 24.914 | 25.522 | 26.233 | 24.756 |  |
| bta-miR-22-3p | . | . | . | 3.449 | 2.574 | 2.433 |  |
| bta-miR-26a | 30.511 | 23.246 | 23.290 | 24.479 | 24.706 | 23.406 |  |
| bta-miR-26b | 30.962 | 24.871 | 24.958 | 25.582 | 26.723 | 24.996 |  |
| bta-miR-29d-3p | 36.988 | 26.236 | 26.712 | 28.332 | 28.893 | 26.808 |  |
| bta-miR-26c | . | 35.526 | 36.636 | . | 34.873 | 35.033 |  |
| bta-miR-29d-5p | . | 30.693 | 30.381 | 32.860 | 31.952 | 30.248 |  |
| bta-miR-27a-3p | 27.986 | 21.419 | 20.967 | 23.361 | 23.308 | 21.566 |  |
| bta-miR-29e | 35.010 | 27.132 | 26.925 | 33.920 | 30.783 | 27.747 |  |
| bta-miR-27a-5p | 34.607 | 29.778 | 30.212 | 30.623 | 31.180 | 29.745 |  |
| bta-miR-301a | . | 31.038 | 31.261 | . | 34.334 | 30.802 |  |
| bta-miR-27b | 30.096 | 22.677 | 22.753 | 24.595 | 24.662 | 22.755 |  |
| bta-miR-301b | . | 31.222 | 31.583 | . | 34.333 | 30.859 |  |
| bta-miR-28 | 36.010 | 27.276 | 27.396 | 28.850 | 29.386 | 27.422 |  |
| bta-miR-302a | . | . | . | . | . | . |  |
| bta-miR-296-3p | 31.743 | 30.801 | 31.790 | 31.240 | 35.188 | 31.940 |  |
| bta-miR-302b | . | . | . | . | . | . |  |
| bta-miR-296-5p | 31.007 | 28.147 | 28.192 | 29.752 | 30.173 | 28.631 |  |
| bta-miR-302c | 34.877 | 35.776 | . | 33.926 | . | 36.070 |  |
| bta-miR-299 | 35.043 | 33.691 | 34.668 | 35.656 | . | 33.542 |  |
| bta-miR-302d | . | 34.549 | . | . | . | 35.707 |  |
| bta-miR-29a | 30.160 | 21.978 | 21.997 | 23.858 | 24.718 | 22.583 |  |
| bta-miR-3064 | 35.575 | 32.935 | 31.695 | 33.858 | . | 34.443 |  |
| bta-miR-29b | 26.635 | 24.103 | 23.817 | 29.956 | 27.670 | 24.795 |  |
| bta-miR-30a-5p | 32.194 | 25.452 | 25.242 | 27.666 | 27.034 | 25.514 |  |
| bta-miR-29c | 29.763 | 22.150 | 22.038 | 23.792 | 24.628 | 22.413 |  |
| bta-miR-30b-3p | . | 35.008 | 34.961 | . | . | 35.602 |  |
| bta-miR-30b-5p | 33.467 | 26.542 | 26.605 | 28.765 | 27.758 | 26.230 |  |
| bta-miR-328 | 33.978 | 30.292 | 30.826 | 31.611 | 33.364 | 30.804 |  |
| bta-miR-30c | 30.798 | 25.715 | 25.763 | 27.222 | 26.902 | 25.740 |  |
| bta-miR-329a | . | 34.291 | . | . | . | 35.044 |  |
| bta-miR-30d | 32.367 | 25.533 | 25.497 | 27.666 | 27.032 | 25.613 |  |
| bta-miR-329b | 35.030 | . | 35.263 | 34.270 | 36.192 | 35.022 |  |
| bta-miR-30e-5p | 32.906 | 25.608 | 25.469 | 27.945 | 26.988 | 25.455 |  |
| bta-miR-330 | 31.731 | 31.281 | 31.059 | 30.860 | 32.836 | 31.747 |  |
| bta-miR-30f | 32.939 | 25.735 | 25.809 | 27.768 | 26.858 | 25.780 |  |
| bta-miR-331-3p | 33.889 | 28.112 | 28.036 | 29.990 | 29.874 | 28.288 |  |
| bta-miR-31 | 30.469 | 24.252 | 24.356 | 25.825 | 26.873 | 24.838 |  |
| bta-miR-331-5p | 34.001 | 30.859 | 30.636 | 31.987 | 32.381 | 30.777 |  |
| bta-miR-32 | 36.036 | 31.870 | 31.359 | 35.597 | 32.856 | 31.547 |  |
| bta-miR-335 | 25.062 | 26.564 | 27.389 | 25.244 | 27.870 | 27.807 |  |
| bta-miR-320a | 30.711 | 25.742 | 25.369 | 26.428 | 27.832 | 25.776 |  |
| bta-miR-338 | . | 34.854 | 35.449 | . | 35.165 | 34.120 |  |
| bta-miR-320b | 33.776 | 35.390 | 34.211 | 33.171 | 35.798 | . |  |
| bta-miR-339a | 32.480 | 28.785 | 29.008 | 30.334 | 30.163 | 28.659 |  |
| bta-miR-323 | . | 31.839 | 32.094 | 33.924 | 35.138 | 32.662 |  |
| bta-miR-339b | 31.418 | 26.817 | 26.802 | 28.942 | 28.122 | 26.668 |  |
| bta-miR-324 | 35.048 | 30.239 | 29.836 | 32.558 | 32.876 | 30.222 |  |
| bta-miR-33a | . | 30.096 | 30.115 | 34.852 | 32.372 | 30.004 |  |
| bta-miR-326 | 30.730 | 31.034 | 32.710 | 30.693 | 34.658 | 32.527 |  |
| bta-miR-33b | 31.628 | 31.066 | 31.071 | 31.915 | 32.926 | 31.620 |  |
| bta-miR-340 | 33.543 | 31.321 | 30.887 | 31.384 | 31.354 | 30.732 |  |
| bta-miR-365-3p | 31.531 | 25.281 | 25.171 | 26.405 | 27.112 | 25.528 |  |
| bta-miR-342 | 32.789 | 24.997 | 25.733 | 26.304 | 27.343 | 25.003 |  |
| bta-miR-365-5p | 33.132 | 32.098 | 31.756 | 32.200 | 36.646 | 33.473 |  |
| bta-miR-345-3p | 34.561 | 32.767 | 32.085 | 32.714 | 34.641 | 32.935 |  |
| bta-miR-367 | . | . | . | . | . | . |  |
| bta-miR-345-5p | 32.665 | 29.791 | 29.329 | 30.738 | 31.048 | 29.247 |  |
| bta-miR-369-3p | . | 35.727 | 35.025 | 35.058 | 35.676 | . |  |
| bta-miR-346 | 27.850 | 30.583 | 31.235 | 28.108 | 33.200 | 31.671 |  |
| bta-miR-369-5p | . | . | 35.761 | . | . | 36.458 |  |
| bta-miR-34a | 30.696 | 24.201 | 23.861 | 26.871 | 26.086 | 24.281 |  |
| bta-miR-370 | 32.674 | 33.110 | 36.103 | 31.539 | . | . |  |
| bta-miR-34b | . | 32.928 | 32.028 | 34.410 | 34.833 | 31.860 |  |
| bta-miR-371 | 33.046 | 32.856 | 33.435 | 32.947 | 36.435 | 33.919 |  |
| bta-miR-34c | 36.085 | 31.536 | 31.733 | 33.929 | 33.987 | 31.839 |  |
| bta-miR-374a | 32.334 | 26.384 | 25.718 | 26.784 | 26.819 | 25.898 |  |
| bta-miR-361 | 30.220 | 26.473 | 26.330 | 27.818 | 27.494 | 26.306 |  |
| bta-miR-374b | 33.081 | 27.653 | 27.297 | 28.225 | 28.217 | 27.452 |  |
| bta-miR-362-3p | . | 30.162 | 29.829 | 32.543 | 31.448 | 30.749 |  |
| bta-miR-375 | 31.823 | 27.044 | 27.600 | 27.776 | 28.664 | 27.036 |  |
| bta-miR-362-5p | 35.462 | 29.750 | 29.648 | 31.031 | 30.875 | 29.647 |  |
| bta-miR-376a | . | 34.008 | 33.767 | . | 36.613 | 34.293 |  |
| bta-miR-363 | . | 31.810 | 31.912 | 33.697 | 35.010 | 32.854 |  |
| bta-miR-376b | . | . | 35.356 | . | . | 33.935 |  |
| bta-miR-376c | . | 35.218 | 34.073 | . | . | 33.917 |  |
| bta-miR-382 | 35.987 | 33.870 | 33.970 | 35.620 | . | 32.938 |  |
| bta-miR-376d | . | 33.579 | 33.581 | . | . | 34.093 |  |
| bta-miR-383 | 33.915 | 34.705 | 34.105 | 33.779 | . | . |  |
| bta-miR-376e | . | 33.226 | 32.898 | 36.076 | 35.581 | 34.954 |  |
| bta-miR-409a | 35.009 | 33.972 | 35.029 | . | . | . |  |
| bta-miR-377 | . | 32.733 | 32.002 | 36.329 | 33.025 | 32.176 |  |
| bta-miR-409b | . | . | 36.889 | . | . | 36.687 |  |
| bta-miR-378 | 30.874 | 24.783 | 24.870 | 26.688 | 25.816 | 24.527 |  |
| bta-miR-410 | 33.980 | 33.942 | 34.242 | 35.197 | . | 34.298 |  |
| bta-miR-378b | 31.694 | 24.699 | 25.084 | 26.679 | 25.947 | 24.583 |  |
| bta-miR-411a | . | 32.943 | 32.165 | . | 32.943 | 32.634 |  |
| bta-miR-378c | 34.920 | 30.329 | 30.404 | 32.889 | 31.508 | 29.582 |  |
| bta-miR-411b | . | 35.759 | 35.128 | . | . | . |  |
| bta-miR-378d | 35.001 | 31.787 | 31.857 | 33.099 | 33.830 | 31.879 |  |
| bta-miR-411c-3p | 36.642 | 34.044 | 32.866 | 35.121 | . | 32.432 |  |
| bta-miR-379 | . | 32.118 | 31.809 | 32.258 | 33.916 | 31.851 |  |
| bta-miR-411c-5p | 36.803 | . | . | . | 35.682 | . |  |
| bta-miR-380-3p | 33.487 | 32.490 | 32.216 | 32.802 | . | 32.937 |  |
| bta-miR-412 | 33.840 | 30.234 | 28.011 | 32.471 | 25.299 | 28.336 |  |
| bta-miR-380-5p | . | 36.688 | 33.776 | 34.942 | 35.247 | 34.144 |  |
| bta-miR-421 | 27.788 | 27.649 | 27.637 | 30.445 | 27.425 | 25.741 |  |
| bta-miR-381 | 33.508 | 33.627 | 33.637 | 32.893 | . | 34.145 |  |
| bta-miR-423-3p | 33.977 | 26.736 | 26.799 | 28.110 | 29.228 | 27.231 |  |
| bta-miR-423-5p | 31.590 | 25.628 | 25.502 | . | 29.431 | 26.578 |  |
| bta-miR-449c | 33.870 | 31.973 | 30.775 | 33.398 | 33.285 | 32.344 |  |
| bta-miR-424-3p | 34.518 | 31.642 | 32.854 | 34.463 | 34.330 | 32.528 |  |
| bta-miR-449d | 30.570 | 29.683 | 30.956 | 30.490 | 35.148 | 31.668 |  |
| bta-miR-424-5p | . | 29.130 | 28.658 | 31.728 | 30.848 | 29.465 |  |
| bta-miR-450a | . | 29.451 | 28.829 | 32.502 | 30.343 | 29.204 |  |
| bta-miR-425-3p | 27.658 | 27.383 | 27.769 | 27.282 | 29.098 | 27.858 |  |
| bta-miR-450b | 35.132 | 29.729 | 29.837 | . | 32.572 | 32.197 |  |
| bta-miR-425-5p | 33.808 | 27.075 | 26.952 | 27.906 | 28.641 | 27.055 |  |
| bta-miR-451 | . | 33.449 | 35.819 | 36.452 | . | 34.350 |  |
| bta-miR-429 | 32.133 | 25.428 | 25.743 | 26.793 | 27.307 | 25.635 |  |
| bta-miR-452 | . | 31.897 | 32.196 | 34.823 | 34.157 | 31.633 |  |
| bta-miR-431 | 31.900 | 31.852 | 31.884 | 31.748 | 32.857 | 32.874 |  |
| bta-miR-4523 | 31.835 | 32.550 | 33.099 | 33.720 | . | 33.014 |  |
| bta-miR-432 | 32.882 | 34.040 | 33.998 | 32.054 | 34.143 | 33.841 |  |
| bta-miR-453 | . | 32.939 | 33.948 | 33.943 | 35.015 | 34.746 |  |
| bta-miR-433 | 31.920 | 32.050 | 31.825 | 31.799 | 34.797 | 33.069 |  |
| bta-miR-454 | 34.298 | 28.795 | 29.134 | 30.542 | 30.960 | 28.988 |  |
| bta-miR-448 | . | . | 34.719 | 34.701 | 34.707 | 36.566 |  |
| bta-miR-455-3p | . | 30.915 | 31.776 | 32.864 | 32.545 | 30.587 |  |
| bta-miR-449a | 28.836 | 27.146 | 26.698 | 28.028 | 28.805 | 27.676 |  |
| bta-miR-455-5p | . | 30.107 | 30.420 | 32.783 | 32.435 | 29.937 |  |
| bta-miR-449b | . | 33.900 | 33.261 | 33.945 | . | 33.157 |  |
| bta-miR-483 | 36.803 | 33.307 | 34.398 | 36.286 | . | 36.099 |  |
| bta-miR-484 | 31.872 | 26.919 | 26.760 | 28.193 | 28.743 | 27.047 |  |
| bta-miR-496 | . | 34.500 | 33.937 | . | 36.528 | 35.579 |  |
| bta-miR-485 | . | 33.921 | 32.399 | . | 32.755 | 32.450 |  |
| bta-miR-497 | 33.212 | 29.552 | 29.460 | 29.749 | 32.706 | 30.507 |  |
| bta-miR-486 | 33.131 | 30.627 | 30.724 | 31.928 | 32.442 | 31.829 |  |
| bta-miR-499 | . | 31.004 | 31.309 | 33.103 | 33.911 | 31.039 |  |
| bta-miR-487a | 35.699 | 36.078 | 34.852 | 33.837 | 35.201 | 33.459 |  |
| bta-miR-500 | 33.945 | 29.578 | 28.779 | 30.205 | 30.552 | 29.083 |  |
| bta-miR-487b | 36.734 | 32.842 | 33.560 | 36.537 | 34.488 | 34.969 |  |
| bta-miR-502a | 35.513 | 29.901 | 28.266 | 34.392 | 29.106 | 28.449 |  |
| bta-miR-488 | 36.397 | 36.586 | . | 34.728 | . | . |  |
| bta-miR-502b | . | 32.900 | 31.794 | 32.327 | 32.784 | 31.878 |  |
| bta-miR-489 | . | 36.601 | 35.201 | . | . | 35.680 |  |
| bta-miR-503-3p | 31.125 | 30.799 | 30.482 | 30.534 | 32.674 | 31.224 |  |
| bta-miR-490 | 31.814 | 31.852 | 32.739 | 32.013 | 35.752 | 34.067 |  |
| bta-miR-503-5p | . | 31.249 | 30.440 | 34.626 | 32.185 | 32.261 |  |
| bta-miR-491 | 33.863 | 30.965 | 29.745 | 32.707 | 32.260 | 30.375 |  |
| bta-miR-504 | 34.442 | 32.603 | 34.434 | 32.871 | 33.967 | 33.863 |  |
| bta-miR-493 | 31.740 | 31.550 | 31.837 | 31.551 | 36.141 | 33.376 |  |
| bta-miR-505 | 32.328 | 29.164 | 28.848 | 30.149 | 30.732 | 29.036 |  |
| bta-miR-494 | 30.291 | 26.846 | 27.492 | 27.046 | 30.391 | 28.148 |  |
| bta-miR-532 | 34.857 | 30.574 | 29.967 | 31.861 | 31.199 | 30.573 |  |
| bta-miR-495 | 36.447 | 34.030 | 33.987 | . | 35.965 | 33.823 |  |
| bta-miR-539 | . | . | . | . | . | . |  |
| bta-miR-541 | 32.637 | 32.676 | 32.311 | 31.758 | 33.682 | 33.381 |  |
| bta-miR-582 | . | 30.318 | 29.285 | 30.785 | 30.731 | 29.832 |  |
| bta-miR-542-5p | 34.800 | 32.199 | 32.697 | . | 36.141 | 32.897 |  |
| bta-miR-584 | 32.128 | 31.628 | 30.805 | 31.326 | 34.410 | 31.807 |  |
| bta-miR-543 | . | 32.668 | 34.198 | . | 35.389 | 33.956 |  |
| bta-miR-592 | . | 36.001 | 33.196 | . | . | 33.853 |  |
| bta-miR-544a | 36.104 | 35.744 | 35.845 | . | 36.078 | 34.244 |  |
| bta-miR-599 | . | . | . | . | . | . |  |
| bta-miR-544b | . | . | 36.513 | 35.612 | . | . |  |
| bta-miR-615 | 5.857 | 5.755 | 5.855 | 5.969 | 5.899 | 6.356 |  |
| bta-miR-545-3p | . | 32.337 | 32.824 | . | 33.314 | 31.769 |  |
| bta-miR-628 | . | 34.916 | 33.186 | 34.604 | . | 34.383 |  |
| bta-miR-545-5p | 36.104 | 30.966 | 31.900 | . | 33.527 | 31.959 |  |
| bta-miR-631 | 31.842 | 32.781 | 33.301 | 32.540 | 35.874 | 32.975 |  |
| bta-miR-551a | . | 36.649 | 36.050 | 34.924 | . | . |  |
| bta-miR-652 | 32.624 | 26.840 | 26.796 | 27.894 | 28.773 | 27.209 |  |
| bta-miR-551b | . | . | . | . | . | . |  |
| bta-miR-653 | . | 35.593 | . | . | . | . |  |
| bta-miR-562 | 33.883 | 36.864 | 35.132 | 33.654 | 35.033 | . |  |
| bta-miR-654 | 20.010 | 20.448 | 20.814 | 19.962 | 21.368 | 21.067 |  |
| bta-miR-568 | 31.868 | 32.809 | 32.886 | 31.908 | 32.873 | 33.405 |  |
| bta-miR-655 | . | . | 34.931 | . | . | . |  |
| bta-miR-574 | 24.714 | 25.601 | 25.978 | 24.644 | 29.074 | 26.746 |  |
| bta-miR-656 | 36.269 | . | 35.393 | . | . | . |  |
| bta-miR-658 | . | 36.498 | 35.582 | 34.360 | 35.956 | 35.673 |  |
| bta-miR-758 | 33.597 | 35.107 | 35.484 | 33.988 | 36.453 | 35.520 |  |
| bta-miR-660 | . | 28.362 | 28.136 | 28.569 | 29.393 | 28.238 |  |
| bta-miR-759 | . | . | . | . | . | . |  |
| bta-miR-664a | 31.603 | 33.437 | 33.073 | 31.833 | . | 35.046 |  |
| bta-miR-760-3p | 32.175 | 31.691 | 31.909 | 31.367 | 33.665 | 32.467 |  |
| bta-miR-664b | 24.442 | 24.857 | 24.796 | 23.872 | 25.534 | 25.119 |  |
| bta-miR-760-5p | 27.143 | 26.566 | 26.663 | 26.767 | 31.818 | 28.137 |  |
| bta-miR-665 | 30.507 | 28.870 | 29.276 | 28.763 | 31.683 | 29.807 |  |
| bta-miR-761 | 33.894 | 33.909 | 34.945 | 33.999 | 34.976 | 33.869 |  |
| bta-miR-669 | 26.522 | 27.779 | 27.934 | 26.856 | 32.584 | 29.196 |  |
| bta-miR-763 | 30.794 | 32.690 | 32.509 | 31.662 | . | 33.933 |  |
| bta-miR-670 | 36.895 | . | 34.999 | 34.985 | . | 34.752 |  |
| bta-miR-764 | . | 36.151 | . | 35.678 | . | . |  |
| bta-miR-671 | 36.719 | 32.498 | 33.619 | 33.944 | . | 34.119 |  |
| bta-miR-767 | 36.062 | 34.641 | 33.975 | 34.211 | 36.095 | 33.665 |  |
| bta-miR-677 | 32.922 | 27.467 | 27.696 | 28.783 | 32.593 | 28.835 |  |
| bta-miR-769 | 33.576 | 31.882 | 31.937 | 32.987 | 34.175 | 32.227 |  |
| bta-miR-7 | 36.341 | 28.388 | 28.198 | 28.960 | 30.874 | 28.304 |  |
| bta-miR-873 | 34.858 | 35.588 | 36.243 | 34.933 | 35.859 | 34.084 |  |
| bta-miR-708 | . | 29.384 | 28.385 | 30.715 | 30.002 | 29.046 |  |
| bta-miR-874 | 32.445 | 32.376 | 31.585 | 31.803 | 33.945 | 32.990 |  |
| bta-miR-744 | 32.836 | 29.265 | 30.439 | 31.829 | 32.790 | 29.896 |  |
| bta-miR-875 | . | . | 33.912 | . | . | . |  |
| bta-miR-876 | 34.840 | 33.919 | 35.233 | 33.371 | . | . |  |
| bta-miR-98 | 32.192 | 27.136 | 27.327 | 27.943 | 29.155 | 27.110 |  |
| bta-miR-877 | 32.708 | 28.956 | 29.306 | 29.764 | 32.489 | 29.645 |  |
| bta-miR-99a-3p | 35.818 | 35.544 | 34.090 | . | . | 34.647 |  |
| bta-miR-885 | 32.180 | 29.999 | 31.592 | 30.645 | 33.611 | 29.999 |  |
| bta-miR-99a-5p | 36.300 | 27.734 | 27.834 | 29.045 | 29.056 | 27.827 |  |
| bta-miR-9-3p | . | . | . | . | . | . |  |
|  |  |  |  |  |  |  |  |
| bta-miR-9-5p | 34.356 | 26.934 | 28.497 | 27.696 | 29.427 | 26.756 |  |
| bta-miR-1179 | 36.464 | 35.929 | 36.843 | . | . | . |  |
| bta-miR-92a | 28.333 | 23.474 | 23.821 | 24.204 | 25.368 | 23.605 |  |
| bta-miR-1185 | . | . | . | . | . | 35.288 |  |
| bta-miR-92b | 27.770 | 25.588 | 25.745 | 26.651 | 27.246 | 25.808 |  |
| bta-miR-1193 | . | . | . | . | . | . |  |
| bta-miR-93 | 31.863 | 24.738 | 24.720 | 26.646 | 25.968 | 24.705 |  |
| bta-miR-1197 | . | . | . | . | . | . |  |
| bta-miR-935 | 17.696 | 17.750 | 17.887 | 17.765 | 18.335 | 18.358 |  |
| bta-miR-122 | 34.957 | 34.644 | 36.054 | 32.852 | 35.207 | 33.863 |  |
| bta-miR-940 | 28.502 | 22.538 | 22.457 | 23.110 | 24.192 | 22.771 |  |
| bta-miR-1224 | 31.536 | 27.275 | 27.493 | 27.047 | 29.081 | 27.457 |  |
| bta-miR-95 | . | 31.563 | 31.822 | 33.387 | 33.654 | 33.037 |  |
| bta-miR-1225-3p | 25.809 | 25.661 | 25.840 | 25.815 | 28.496 | 26.475 |  |
| bta-miR-96 | 33.864 | 26.487 | 26.554 | 29.911 | 30.165 | 27.771 |  |
| bta-miR-1246 | 27.154 | 22.117 | 22.760 | 21.395 | 23.809 | 22.014 |  |
| bta-miR-1247-3p | 31.587 | 30.808 | 30.547 | 30.755 | 33.095 | 31.099 |  |
| bta-miR-1296 | 31.100 | 30.045 | 31.539 | 30.689 | 33.487 | 31.849 |  |
| bta-miR-1247-5p | 32.928 | 27.569 | 27.698 | 29.260 | 29.535 | 27.408 |  |
| bta-miR-1298 | 36.284 | . | . | 35.982 | . | . |  |
| bta-miR-1248 | 34.245 | 32.857 | 32.855 | 32.602 | 35.203 | 33.378 |  |
| bta-miR-1301 | 33.891 | 34.586 | 36.681 | 36.286 | . | . |  |
| bta-miR-1249 | 33.493 | 27.757 | 28.565 | 29.597 | 29.510 | 27.837 |  |
| bta-miR-1306 | 32.388 | 28.611 | 29.199 | 29.773 | 29.919 | 28.747 |  |
| bta-miR-1260b | 25.697 | 22.204 | 22.121 | 23.136 | 23.808 | 22.422 |  |
| bta-miR-1307 | 33.069 | 27.796 | 28.212 | 28.751 | 30.985 | 28.744 |  |
| bta-miR-1271 | 35.642 | 30.829 | 31.143 | 33.805 | 32.927 | 30.973 |  |
| bta-miR-1343-3p | 32.314 | 28.804 | 29.163 | 29.838 | 31.174 | 29.577 |  |
| bta-miR-1277 | . | . | . | . | . | . |  |
| bta-miR-1343-5p | 31.880 | 26.835 | 26.717 | 26.736 | 29.841 | 27.919 |  |
| bta-miR-1281 | 28.591 | 28.801 | 28.949 | 28.538 | 30.934 | 29.834 |  |
| bta-miR-1388-3p | 33.876 | 30.681 | 30.793 | 30.981 | 32.119 | 31.286 |  |
| bta-miR-1282 | 34.920 | 35.124 | 35.901 | 36.209 | . | . |  |
| bta-miR-1284 | 33.480 | 33.717 | 35.916 | 33.729 | 35.277 | 32.917 |  |
| bta-miR-1287 | 32.774 | 33.928 | 33.932 | 32.626 | 36.517 | 35.793 |  |
| bta-miR-1291 | 34.983 | 34.026 | 33.973 | 33.224 | . | . |  |
